# From gene expression to gene regulatory networks in *Arabidopsis thaliana*

**DOI:** 10.1186/1752-0509-3-85

**Published:** 2009-09-03

**Authors:** Chris J Needham, Iain W Manfield, Andrew J Bulpitt, Philip M Gilmartin, David R Westhead

**Affiliations:** 1 School of Computing, University of Leeds, Leeds, LS2 9JT, UK; 2 Institute of Integrative and Comparative Biology, University of Leeds, Leeds, LS2 9JT, UK; 3 Institute of Molecular and Cellular Biology, University of Leeds, Leeds, LS2 9JT, UK; 4 Current address : School of Biological and Biomedical Sciences, Durham University, Durham, UK

## Abstract

**Background:**

The elucidation of networks from a compendium of gene expression data is one of the goals of systems biology and can be a valuable source of new hypotheses for experimental researchers. For *Arabidopsis*, there exist several thousand microarrays which form a valuable resource from which to learn.

**Results:**

A novel Bayesian network-based algorithm to infer gene regulatory networks from gene expression data is introduced and applied to learn parts of the transcriptomic network in *Arabidopsis thaliana *from a large number (thousands) of separate microarray experiments. Starting from an initial set of genes of interest, a network is grown by iterative addition to the model of the gene, from another defined set of genes, which gives the 'best' learned network structure. The gene set for iterative growth can be as large as the entire genome. A number of networks are inferred and analysed; these show (i) an agreement with the current literature on the circadian clock network, (ii) the ability to model other networks, and (iii) that the learned network hypotheses can suggest new roles for poorly characterized genes, through addition of relevant genes from an unconstrained list of over 15,000 possible genes. To demonstrate the latter point, the method is used to suggest that particular *GATA *transcription factors are regulators of photosynthetic genes. Additionally, the performance in recovering a known network from different amounts of synthetically generated data is evaluated.

**Conclusion:**

Our results show that plausible regulatory networks can be learned from such gene expression data alone. This work demonstrates that network hypotheses can be generated from existing gene expression data for use by experimental biologists.

## Background

Much of molecular biology aims to decipher the mechanisms organisms use to modulate their gene expression patterns. This has been greatly facilitated by genome sequencing and subsequent design of microarrays allowing determination of gene expression patterns with near full-genome coverage. While individual array experiments can be examined for differential expression of genes of interest, this may be misleading owing to irreproducibility, and only uses a small fraction of the data often available. Further analysis of large amounts of microarray data *en masse *to calculate correlation coefficients can be used to rank genes according to how closely their expression follows that of a query gene of interest. Strongly correlated genes are likely to be expressed in a similar manner and may indeed share a common function or regulatory mechanism; over-representation of GO terms and promoter motifs can support such a prediction [1]. Although it is not often possible to determine using co-expression analysis alone which transcription factors mediate this regulation, it is clear that large bodies of microarray data contain information which may allow reconstruction of regulatory networks. The Affymetrix ATH1 array service from the Nottingham Arabidopsis Stock Centre (NASC) represents an unusually valuable resource with RNA samples provided by many different researchers, labelled, hybridized and analysed by the NASC arrays team [2] to create a very large and diverse data set with consistent use of identical protocols within a single laboratory. The elucidation of networks from such a repository of data is one of the goals of systems biology and can be a valuable source of new hypotheses for experimental researchers.

*Arabidopsis thaliana*, the model plant, is a good example of the challenges of network reconstruction. The genome contains around 28,000 genes including around 2,000 transcription factor genes and many other genes encoding proteins with regulatory roles. Only a small proportion of these genes have been analysed by phenotypic characterization of mutants and fewer still have been subjected to microarray analysis to determine the groups of genes which are mis-regulated in these mutants. Even for well-characterized genes, new roles can be found; furthermore, for well-characterized networks, new members continue to be added. The cost and time to make and analyse mutants in *Arabidopsis *combined with the scarcity of phenotypic effects of mutagenesis means that computational tools suggesting candidate genes and roles are particularly useful.

Much research into inferring networks from data, particularly gene expression profiles has been undertaken  [3-6]). In a review by Bansal *et al*. [7] various approaches to reverse engineering networks, including Bayesian networks (BNs) and ordinary differential equation (ODE) models are discussed and applied to a range of datasets including experiments from steady state, time-series and perturbations. Time series data allows a dynamic process to be modelled. Both ODE and DBN (dynamic Bayesian network) models have been shown to be good at this task. The extra temporal information available from time series data allows the learning of causal relationships between variables (genes) from smaller datasets. Perturbation experiments where a particular gene's expression level is altered, through mutation, over expression or RNAi knockdown, provide valuable information for inferring directionality of relationships between genes. Sachs *et al*. [8] learn protein signalling networks from protein flow cytometry data using perturbations and expert knowledge. Incorporating information from other heterogeneous data sources has also shown to be valuable for network inference [9,10]. There are many other data sources which could be incorporated (sequence, ChIP-chip, knowledge from literature, etc), but in the case of *A. thaliana *other data and network related literature are limited.

Some approaches to identify gene regulatory networks in *Arabidopsis *are covered in recent reviews [11,12]. These vary from learning association networks; Gaussian graphical models/partial correlation networks, such as GeneNet [13]; using a differential equation model and employing a singular value decomposition technique to identify the most consistent network across multiple time series datasets [14]; and growing Bayesian networks from seed genes by identifying conditional (in)dependence relations between genes in order to identify the parents and children of the seed gene and to iteratively increase the radius of the network around the gene [15]. Details of some these methods and their application to our data is contained in the Discussion.

Here, gene regulatory networks are learned for *Arabidopsis *from NASC microarray data. Starting from a small set of initial genes of interest, a network is learned in the form of a static Bayesian network, and genes from an extended list are iteratively added to the network - with the gene that leads to a model that is statistically most likely to have generated the observed data being added at each iteration. To test the approach we apply it to the circadian clock network, which is probably the best-characterized regulatory network in *Arabidopsis*. It enables the plant to optimize gene expression patterns and consequently physiology for different times of day. The genes in this network have been extensively characterized by expression analysis, protein abundance and analysis of multiple mutant alleles (reviewed in [16,17]). In addition, modelling and experimental verification of properties of key regulators has been performed [18-20]. This body of work provides a benchmark against which modelling of larger numbers of genes can be assessed. The early models of plant clock regulation comprised reciprocal regulation of TOC1 by the partially redundant Myb transcription factors LHY and CCA1 while expression of these two genes was in turn repressed by TOC1. Current models of the clock have added additional interactions involving firstly TOC1 and GI and secondly CCA1/LHY and PRR7/PRR9. Additional components feed in light signals to modulate clock behaviour [16,17]. Despite the extensive analysis of the clock network, new roles are regularly identified for previously-characterized factors and unidentified factors have been proposed to fill deficiencies in ODE-based models [18-20]. Nevertheless, this represents the best available standard against which we will validate the networks we learn from gene expression data.

Progressing from the clock network, we then apply the method to less well characterized genes to suggest functional and regulatory roles. An important example is the photosynthetic apparatus, which is central to the biology of plants, and is strongly light- and clock-regulated, yet the factors mediating this regulation are incompletely characterized. The promoters of many genes encoding components of the photosynthetic apparatus contain conserved GATA sequence motifs recognized by members of the *GATA *family of zinc finger transcription factors (reviewed in [21]). The *Arabidopsis *genome contains genes for 29 *GATA *family members, but, to date, it has not been conclusively demonstrated which *GATA *gene (or genes) mediates the regulation of these photosynthetic genes [21,22]. Indeed the recent identification of roles for some *GATA *genes in seed germination and floral meristem development [23,24] highlights a need for bioinformatics tools able to generate testable hypotheses of gene function.

## Results

**Figure 1:**
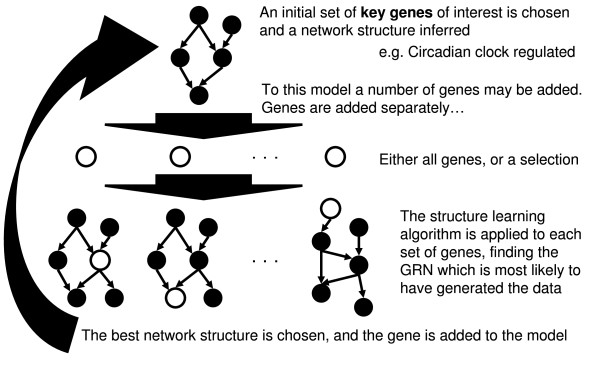
**Overview of incremental network learning algorithm**.

**Figure 2:**
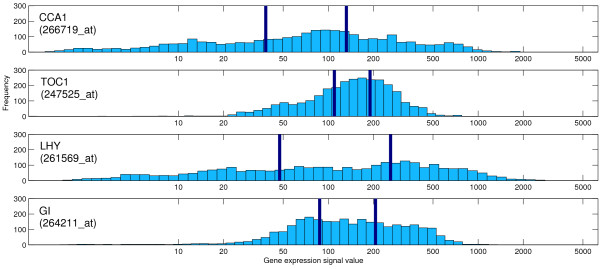
**Gene expression histograms**. Gene expression histograms for the four probes detecting the RNA transcripts of the four clock genes in Figure 3(a). The plot shows the gene expression signal value (on a log10 scale) versus frequency from the 2904 examples. The different class thresholds are highlighted in dark blue for each gene, splitting each gene's signal values in to three equally sized classes.

We demonstrate that from the large (2904 array hybridizations) NASC microarray gene expression data set, we can infer biologically sensible networks in the form of static Bayesian networks. Three examples are presented. Firstly, focusing on the circadian clock, secondly, looking at other networks and poorly characterized genes from a selection of 37 genes chosen from the literature, and thirdly, learning a network relating the poorly characterized *GATA *genes with the clock, and linking them into the photosynthetic network by iterative addition from an unselected set of 15,000+ genes, representing the entire genome for which data is available. In addition to this, the algorithm is benchmarked against a ground truth model. An overview of the network learning algorithm is given in Figure 1 (see Methods for full details). This novel approach to iteratively add genes to the network is enabled through a discretization mechanism for the gene expression data. Through quantising the gene expression values into three equally sized discrete classes on a per gene basis, the entropy of each quantized expression profile is equal, and model comparison can be performed without bias, allowing model selection of the 'best' scoring network with an additional gene from the set (example gene expression histograms and class thresholds for four genes are shown in Figure 2). In the network hypotheses presented in the figures, CPDAGs (Completed Partially Directed Acyclic Graphs) are shown, representing an equivalence class of the actual DAGs (Directed Acyclic Graph) learned by the Bayesian network.

### The *Arabidopsis *circadian clock

**Figure 3:**
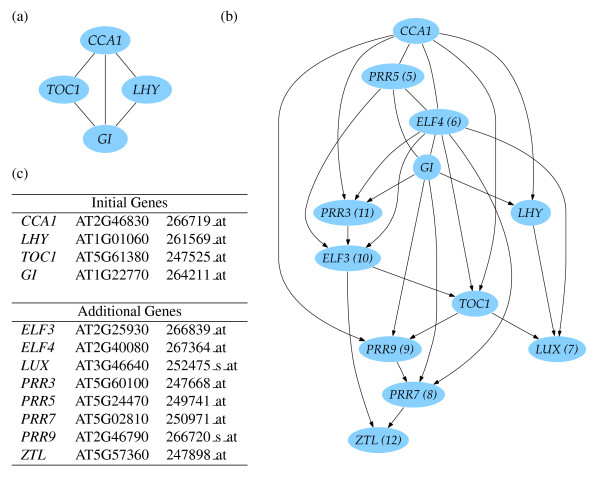
**Learned regulatory network for the *Arabidopsis *circadian clock**. (a) learned from the initial four genes (*LHY*, *CCA1*, *TOC1*, *GI*) only, (b) learned after addition of the other genes (the number in parentheses next to the gene name denotes the order it was added to the network, by the incremental network growing algorithm), and (c) the list of Gene symbol, AGI code, and probe set for *Arabidopsis *circadian clock genes.

To test the effectiveness of the algorithm in predicting regulatory networks, we selected genes encoding regulatory protein components of the circadian clock in *Arabidopsis *(Figure 3c). From an initial set of four genes encoding components of the clock, the structure of a Bayesian network was learned from the quantized gene expression data. At each iteration of the algorithm, one gene was added to the model - the gene which when added to the current genes resulted in the 'best' learned network (the one with the highest BIC score (Bayesian Information Criterion; see Methods)). Figure 3b shows the final output of the algorithm; the network learned for all the genes in the list in Figure 3c. It also shows the order that these clock genes were incorporated into the model.

The output from the learning algorithm is a set of genes connected to each other where the experimental data indicate a predictive relationship between their transcript abundances; arrows indicate the direction of this relationship. They can indicate similar (co-)expression patterns, negative correlation and may perhaps represent regulatory interactions. In the case where the edges are undirected, it indicates that there are several Markov equivalent networks which are equally good, and that these undirected edges are not compelled to indicate a particular direction to the interaction. This is illustrated with a learned network comprising a small set of key clock genes (Figure 3a). This network is a highly simplified model, as it is the result of learning a model that captures the joint probability distribution over just these four genes. *CCA1 *and *LHY *are connected to each other, reflecting their very similar expression patterns and as also reflected by strong correlation of expression [25]. In contrast, *CCA1 *and *TOC1 *are connected although their expression patterns are anti-correlated with peaks of transcript abundance at different times of day. The lack of an edge between *LHY *and *TOC1 *indicates that *LHY *adds no further information about *TOC1 *expression, consistent with models in the literature which treat *LHY *and *CCA1 *as equivalent. As extra genes are incorporated into the network, PRR5 and ELF4 are incorporated first, indicating that these are the most closely related genes, and Figure 3b shows that after all the clock genes in this limited set are added, these two remain highly connected central to the initial four key genes, with the link from CCA1 to GI replaced by links from CCA1 and GI to PRR5 and ELF4.

Multiple iterations to add all of the selected clock related genes, gives a network structure (Figure 3b) which can be compared with current views of the clock in the literature. In this larger network, *CCA1 *and *LHY *show different connections to other genes (such as *TOC1*) although they are semi-redundant [26] and in modelling studies are often assumed to behave identically. Recent publications suggest there may be differences in their behaviour as part of the clock's ability to maintain phase lengths around 24 hours at different temperatures [27]; we speculate that the different connections we see for *LHY *and *CCA1 *may reflect these properties. There is a connection between *GI *and *LHY*, consistent with current three-loop models of the circadian clock, although a *CCA1*-*GI *connection is absent. Owing to the redundancy arising from large multi-gene families in *Arabidopsis*, informatics tools generating hypotheses about how duplicated genes may differ in their roles are very useful. The *CCA1*-*LHY *connection is present in each iteration of network learning although connections between other genes are sometimes observed to change. Similarly, *PRR7 *and *PRR9 *are two similar genes, modelled as if behaving identically in other work [20]; the algorithm connects these genes throughout each iteration of network expansion, consistent with their similar expression patterns but with each gene showing different connections to other genes of the network.

The first clock feedback loop model in the literature consisted of *CCA1*/*LHY *and *TOC1*; consistent with this, we see a *CCA1*-*TOC1 *connection in most iterations (see Additional file 1: mini-website of all resulting networks), although *ELF4 *is inserted between them in some iterations. This latter interaction suggests a candidate for a proposed component of an interlocking three-loop model [28] and is supported by recent experimental evidence [29]. These results demonstrate that the algorithm is able to identify biologically sensible relationships between genes from a large microarray dataset not specifically tailored for this purpose.

### Learning regulatory networks - other networks and poorly characterised genes

We were also interested to analyse some poorly-characterized genes, where there is little information on function or mutant phenotypes but where there is some transcript abundance information allowing an assessment to be made of the accuracy of network predictions. For this analysis, we have selected five *GATA *transcription factor genes (*GATA2*, *GATA4*, *GATA12*, *GATA21 *and *GATA22*) which are subject to differing light and clock regulation [21].

Having shown that the network algorithm could generate biologically sensible relationships for clock genes, we also selected genes from other regulatory networks including cold/salt-responsive genes, light up-regulated and light down-regulated genes; (see Additional file 1: lists of the gene names, AGI codes and Affymetrix probe IDs used in each learning algorithm run are contained in the mini-website). We have again used a set of clock genes to initiate network learning as this was effective in the previous experiment. Many of the light-and stress-responsive genes are clock-regulated; indeed light is one of the key inputs for modulating the properties of the clock and response to cold is more important at night, thus many of these genes and networks would be expected to be interconnected. Four clock and five *GATA *genes were used to initiate network learning and genes added to the network iteratively from a list of 37. Genes used to initiate the learning are guided by what is biologically of interest. Often we find that a handful of genes are thought to be key to a study, and one wishes to find a network into which these genes fit well.

**Figure 4:**
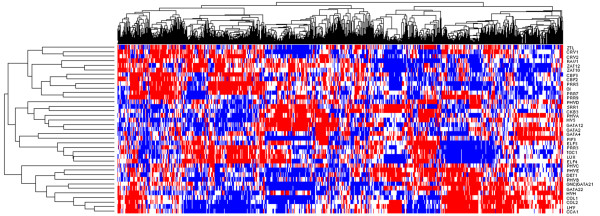
**Clustergram of quantized gene expression profiles for 37 genes of interest, over 2904 microarrays**. Both genes and experiments have been clustered. The three classes representing the low, medium and high classes are coloured blue, white and red respectively.

A cluster dendogram showing the discretized expression values of each gene into low (blue), medium (white) and high (red) categories across the 2904 array dataset is shown in Figure 4. This figure demonstrates the complexity of the relationships between the 37 genes. The dataset contains microarrays from a number of experiments, shown clustered in the columns; note that sets of these may represent a time-series or experiments on similar tissues, or perturbations of genes involved in a particular process. Note however that no explicit use of the information about the experiments is used in this work; each microarray is treated as independent.

**Figure 5:**
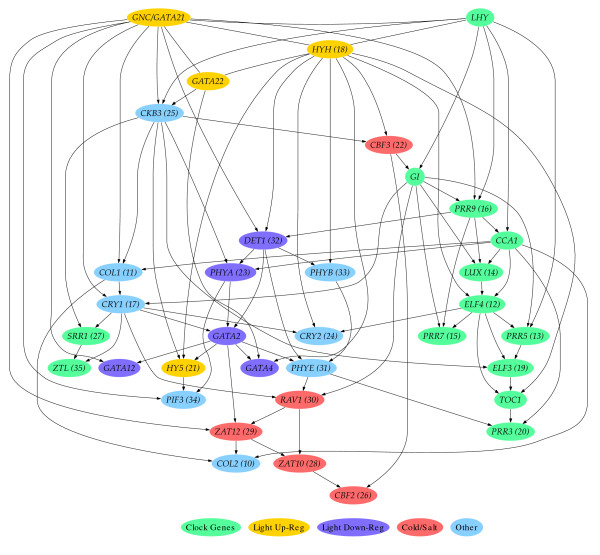
**Learned regulatory network for other networks and poorly-characterized genes**. The learned network structure starting from a set of nine genes (four clock and five *GATA *genes of interest), with additional genes added to the network from a selection of 37 genes. The number in parentheses next to the gene name denotes the order it was added to the network. Most of these genes were added to the network in early iterations, however, genes such as *SRR1 *and *ZTL *with *bona fide *roles in the clock were added late and only indirectly linked to other clock components. All these interactions are very similar throughout the later iterations, once most of these components have been added to the network.

The network structure for the final iteration is shown in Figure 5. Notably, all the clock genes are linked to at least one other clock gene and more extensively linked to each other than to other network components. Despite adding the additional clock genes from a list containing genes from a range of networks, the clock genes are linked in ways we would expect; both *CCA1 *and *LHY *are linked to *TOC1 *in a manner similar to the early models of the Central Oscillator; *TOC1 *is linked to *GI *and *ELF4 *as proposed for interconnected loop models; *CCA1 *is linked to *PRR7 *and *PRR9 *as proposed for the most recent three-loop models [20]; finally, other genes such as *PRR3*, *PRR5*, *ELF3*, *COL1 *and *COL2 *are also linked to the best-characterized clock components.

There is a trend to add genes in thematic blocks in more-or-less sequential iterations, as mentioned above for early addition of most clock genes to the network (see Additional file 1: mini-website of learned GRN at each iteration). The genes within each of these blocks tend also to be linked to each other and therefore they tend to be clustered in the network structure. It is likely that, once one gene has been added (for example with a link to a different sub-network), then addition of related genes adds to the network score. It appears therefore that the learning algorithm is able to correctly group genes according to their regulatory roles.

The *GATA *genes have connections to some well-characterized genes allowing an assessment to be made of the quality of predictions. *GNC*/*GATA21 *and *GATA22 *have connections to genes encoding key regulators of light-induced development, *HYH *and indirectly *HY5*. All of these genes are strongly expressed in green, photosynthetic tissue, and the undirected edges in the CPDAG indicate that there are a number of computational network models that are equivalent, indeed *GNC*/*GATA21*, *GATA22 *and *HYH *are generally co-expressed. In contrast, *GATA2*, *GATA4 *and *GATA12 *have connections to each other and from some well-characterized genes which are down-regulated by light such as *PHYA *and *DET1*. The algorithm has therefore grouped this sub-set of light and clock-regulated *GATA *genes, placing them in appropriate positions within the network.

A selection of genes with well-characterized roles in regulation of plant responses to cold and salt-stress [30] were added to the network in nearly-sequential iterations and with connections to each other. These genes show circadian-regulated changes in transcript abundance with peaks at different times of day. Two of these stress-response genes show connection to key clock component *GI *consistent with the ability of plants to respond to stress being regulated by the time of day the stress is applied [31].

The ability of the algorithm to identify well-characterized relationships from a number of different networks suggests that it should also be able to identify relationships between components of other regulatory networks. Moreover, the observation that the poorly-characterized *GATA *factor genes are correctly placed with respect to other better characterized genes, suggests that the algorithm has the power to predict relationships for uncharacterized genes.

### Learning regulatory networks from an unselected list of 15,000+ genes

Our interest in the possible roles of a number of *GATA *genes led us to perform network learning starting with the same nine genes as in the previous analysis; four clock genes and five *GATA *genes - *GATA2*, *GATA4*, *GATA12*, *GATA21 *and *GATA22*. This time, we allowed any gene from a list of all possible genes which passed the expression distribution filter to be added to the network (see Methods for full details).

**Figure 6:**
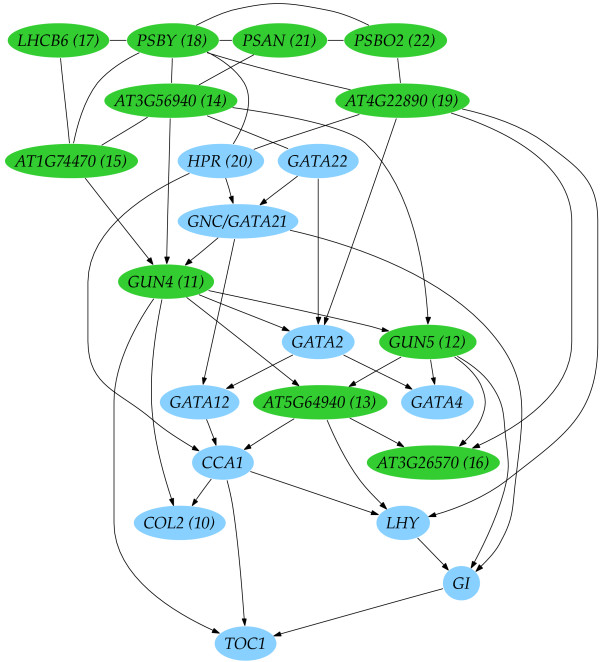
**Learned regulatory network from an unselected list of 15,000+ genes**. The learned network structure starting from a set of nine genes (four clock and five *GATA *genes of interest), with additional genes added to the network from an unselected set of 15,000+ genes - all the probes on the microarray after filtering out low entropy signals. The number in parentheses next to the gene name denotes the order it was added to the network. The nodes coloured green signify that the genes are localized in the chloroplast. It is also worth noting that the large number of undirected edges indicates that there are many equivalently predictive networks which model the data equally well particularly between these chloroplast localized genes.

**Figure 7:**
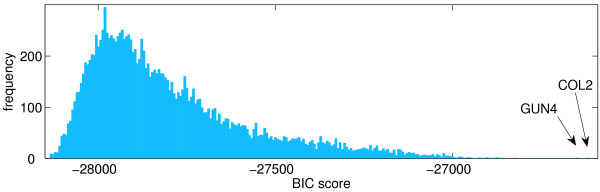
**Distribution of BIC Scores**. Histogram of BIC score for best network learned from when each gene is added to the initial set of nine genes to form a ten node network. *COL2 *is added to the network first, as it has the highest BIC score.

The algorithm was run to include 22 genes (Figure 6). *COL2 *was added first and linked to *CCA1 *(a well-characterized clock component) and this connection was also seen in the previous experiment from a list of 37 genes. *COL2 *has itself been proposed to have roles in clock regulation of gene expression [32]. In addition to clock roles for *COL2*, *GUN4 *(added second) was identified in screens for genes with defects in signalling between chloroplast and nucleus [33]. Of the other genes added to the network, most are chloroplast localized and many encode enzymes of chlorophyll biosynthesis or encode proteins of the photosynthetic apparatus, coloured green in Figure 6. There is therefore a clear theme to the roles of the proteins encoded by these genes. This gives confidence that biological relationships between these genes have been identified, although the algorithm is selecting from a very large number of genes with the possibility of identifying spurious relationships. Indeed, through analysis of the distribution of network likelihoods, we can gain an insight into the confidence of our addition of genes to the network. The algorithm calculates a BIC score for addition of each gene from the list of 15,163 remaining genes to the network and a distribution of BIC scores for each candidate gene in the first iteration is shown in Figure 7. The BIC score for the first gene added (COL2) is well separated from the distribution, making this a clear unique candidate for addition to the network. This property of the distribution of scores may provide insight into why the algorithm works well in this case. The gene with the second best score at this first iteration, *GUN4*, was added to the network at the next iteration (although since the scores are recalculated at each iteration this is not always the case).

These photosynthetic genes incorporated into the model are closely linked to *GATA21 *and *GATA22 *- two genes expressed most strongly in photosynthetic tissue and with peak transcript abundance before dawn [21]. They are therefore better candidates to be regulators of photosynthetic genes than the other *GATA *genes included in the analysis which are more strongly expressed in the dark and with evening phased clock regulation. The *GNC *(*GATA21*) mutant shows reduced chlorophyll levels, although without showing mis-regulation of the genes encoding the photosynthetic apparatus [22]. It is likely that mutation of both *GATA21 *and *GATA22 *is necessary to produce gene mis-regulation and this is experimentally testable. Indeed, after completion of these analyses, the phenotype of a *GATA21*/*GATA22 *double mutant has been reported, showing lower chlorophyll levels than each single mutant [34]. Thus from computational analysis of microarray data alone, we are able to suggest roles for these poorly characterized genes.

### Evaluation on realistic synthetic data

**Figure 8:**
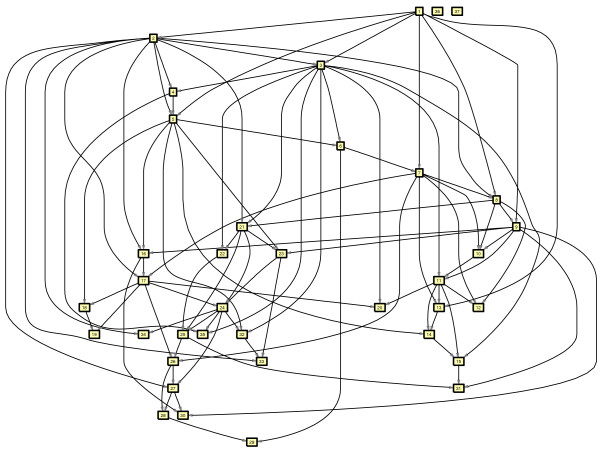
**The underlying ground-truth network structure for the synthetic evaluation example**.

**Figure 9:**
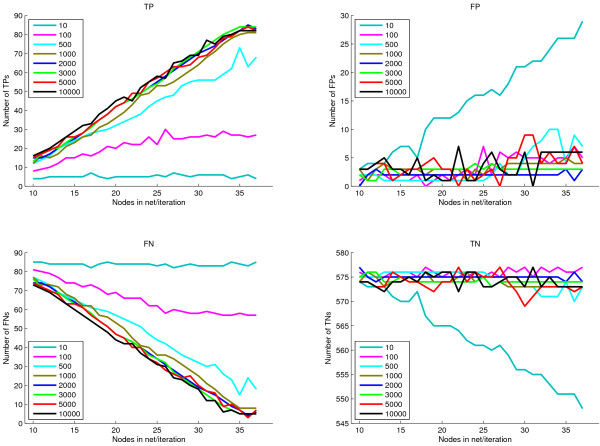
**Evaluation metrics for incrementally growing inferred networks**. Plots of TP/FP/FN/TN counts for networks inferred from different size datasets. All looks fairly monotonic and sensible. Good performance is obtained when learning from 1000+ samples. Poor performance on 100 or fewer samples.

**Figure 10:**
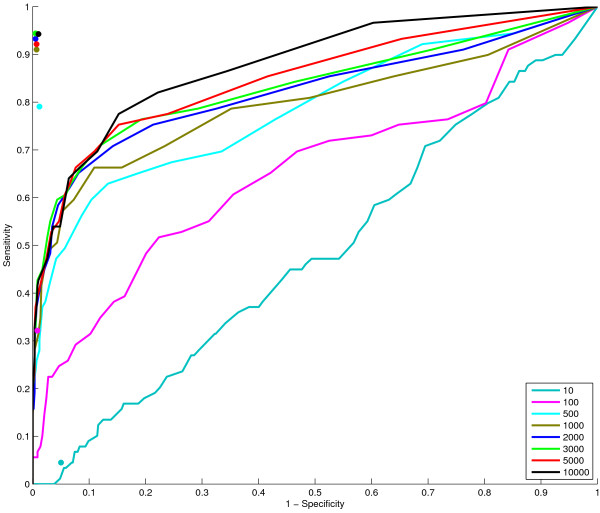
**Correlation ROC curves and Bayesian network results on synthetic data**. The lines show ROC curves for correlation networks inferred from the different size datasets. The asterisks denote the performance of the Bayesian network on all nodes inferred from the same datasets.

For evaluation, we choose a network with the same structure as a learned network. We focus on a network similar to the second example on real data presented earlier. We construct a network with the same structure on 35 nodes, and add in two extra unconnected nodes, giving a network on 37 nodes (Figure 8). From this 'ground truth' network a Bayesian network with CPTs drawn from a similar distribution to those learned from real data (see Methods for full details) is formed and synthetic datasets are generated by sampling the Bayesian network. Next, networks are inferred from this data as in the previous examples on *Arabidopsis *data. Excellent results are obtained when comparing the inferred networks with the underlying ground truth network. (This was not possible on the main examples in the paper due to the lack of a gold standard ground truth). Figure 9 shows the true positive (TP), false positive (FP), false negative (FN) and true negative (TN) counts for networks inferred from different size datasets taking into account edge direction on CPDAGs (as defined in the Methods section on Evaluation metrics). Here it can clearly be seen that network inference from 100 or fewer examples is poor, containing a small proportion of true positive edges. Once 1000 samples or more are used for inference, good results are obtained (extra samples give a slight further improvement). The graphs are generally quite smooth reflecting the fact that large changes in the inferred network are not occurring as additional nodes are incorporated into the network, as is also shown in the GRNs at each iteration (see Additional file 1: mini-website of learned networks at each iteration). For the networks with 35, 36, or 37 nodes, the two unconnected nodes do not get added into the network and similar networks are obtained in each of these iterations, giving 3 FP, 5 FN, 84 TP, & 574 TN when using the dataset with 3000 samples (similar number to the real microarrays used). This equates to a sensitivity of 0.944 and specificity of 0.995. This is considerably better than the results from inferring a correlation network. Figure 10 shows a ROC curve for correlation networks in which more edges are added as the correlation threshold is decreased from 1 to 0. Also plotted on this figure are the sensitivity and specificity results for the Bayesian network inference on the full set of genes. This illustrates clearly the superior performance at inferring the network. It must be noted also that the score for the Bayesian networks considers the directionality of the edges - if a compelled edge is oriented incorrectly then it counts as a false positive edge, whereas in the correlation network, no directionality is considered.

## Discussion

We have developed an algorithm which correctly identifies biological relationships between genes of different regulatory networks using a very large microarray dataset. In these networks, many components of the clock network are linked and often in ways consistent with current models of the clock based on experimental evidence. The biologically sensible linking of genes from other networks (e.g. photosynthesis and stress) suggests that the algorithm should be able to make good predictions for genes from networks we have not analysed. It is not possible to model genes not represented by probe-sets on the Affymetrix ATH1 array or which have been filtered out by the data quantization process, but nevertheless, probe-sets for regulatory genes controlling other processes have survived the quantization filter, including probe-sets for genes active in very specific or minor cell types such as shoot and floral meristems. The quality of results presented shows just how well networks can be learned from large data sets alone. These networks can function as hypotheses that might be tested by specifically designed expression experiments, for instance time series or genetic perturbations.

The learning of large networks is computationally hard: even with only a few nodes, there are too many possible networks to exhaustively search them. Thus methods for learning network structures incrementally have promise. The greedy search method usually finds the same underlying network structure when an additional gene is introduced, although a new network is learned at each iteration with no constraint of similarity to networks from previous iterations. This demonstrates the robustness of the network predictions for similar sets of genes, and suggests that a much more efficient structure learning scheme could be developed by seeding the greedy search with the best DAG from the previous stage (plus the extra node). The learned static Bayesian network consists of a 'best' network structure *S*^*h*^, and a point estimate of the model parameters , which are a set of conditional probability tables (CPTs). From these it is possible to identify the type of relationship between genes (and in turn, distinguish positive from negative regulatory interactions).

The large number of microarrays in the NASC dataset plays a key part in the performance of the network inference. This data set is unique in its large size, diversity of content, uniform production and analytical treatment. The largest single experiment in the dataset we have used comprises 66 array hybridizations, only representing <3% of the entire number of arrays. Furthermore, this experiment analyses whole and dissected flowers (sepals, petals, stamens, carpels and pollen) of two different ages, thus covering a wide range of developmental stages and therefore expression patterns [35]. Similar arguments apply for the six largest experiments (totalling 322 arrays) indicating that these large experiments contribute diversity of expression and will not therefore bias learning algorithm outputs. Some experiments cover circadian and diurnal time courses using material with very similar morphologies but significantly different expression patterns (e.g. [36]) and which are relevant in the context of our analyses. Similarly, many experiments use RNA derived from seedlings, for example, which are morphologically similar but with very different treatments. It is clear therefore that experiments using material from the same developmental stages cannot be seen as redundant. These arguments underpin our approach of analysing the dataset *en masse*.

Other recent research with a similar motivation is the identification of a B cell network [6] through the use of ARACNE [37] and network analysis of the mouse transcriptomic network [38]. Both of these works identify association networks which contain no directionality to the edges in the networks. ARACNE adds edges between genes based on pairwise mutual information above a threshold, and prunes away the weakest edges in each triple of connected genes; they show that this removes many False Positive (FP) edges (the scope for false positive edges in sparse networks is large because there are so many possible networks). Freeman *et al*. [38] present ways to visualize and cluster on networks to identify components of networks and identify biological relationships. Our work uses Bayesian networks to model the regulatory transcriptomic networks, which allow the identification of the directionality of (some of) the regulatory relationships. Bayesian networks have been applied to several datasets, for example, learning networks from 76 arrays of the *S. cerevisiae *cell-cycle [20], where the multinomial models use data discretized into three classes based on differential expression. Our analysis shows good results for learning a static Bayesian network at each iteration for a given set of genes, partly owing to the large and diverse microarray data set used. The quantization of the gene expression signals into three equal sized classes on a per gene basis, in order to ensure equal entropy of each gene, allows for incremental growing of the Bayesian networks in this special case. Bayesian networks are known to discover networks with good accuracy. In a recent evaluation, Werhli *et al*. [39] showed BNs to have good performance at recovering a network based on the Raf signalling pathway identified in [8] from synthetic data generated in a number of ways, and documented the differences in performance for learning from observational and interventional datasets. They evaluated relevance (correlation) networks (RNs), Gaussian graphical models (GGMs)/partial correlation networks and Bayesian networks, and showed that on small datasets (100 examples) Bayesian networks outperform GGMs and RNs for interventional data. In this work, we first compare our results to a widely accepted current network model in *Arabidopsis*, that of the circadian clock, to validate our approach, and secondly on synthetic data with similar properties generated from a known network. The compendium of microarrays used here contains a host of over-expressed and knockout mutant experiments, as well as a wide range of environmental influences, and as such we expect Bayesian network inference to perform well - particularly due to the size of the dataset. Correlations are valuable, and resources such as ACT [25] have been widely used by *Arabidopsis *researchers to identify co-expressed genes. GGMs tend to have been used on expression data from small specifically designed experiments where important factors have been perturbed, and there is not enough data to consider a Bayesian network. These show a marked change in expression, and the correlations between genes can be found, and partial correlations used to distinguish direct form indirect correlations. One advantage of Bayesian networks over correlation or partial correlations is that they do not just capture pairwise relationships between the genes. They allow combinatorial effects between genes to be found. They capture a set of relationships that allows prediction of the states of genes based on a (limited) number of other genes they depend upon, and structure learning allows the elucidation of dependencies between genes. These dependencies can be found, particularly where there is an increase in predictive performance, through a particular dependency structure. The score of different networks incorporates both a likelihood term and a complexity term such that for each gene its main relationships with other genes are captured. With association networks, a single threshold tends to need to be specified, resulting in some parts of the network being densely connected and other genes of interest being unconnected as all correlations with other genes are below that threshold. Our method, specifically aims to try to identify a network into which relevant genes are added, to identify dependencies between variables which best encode the joint probability distribution over all the genes included as opposed to identifying sets of pairwise relationships.

We investigated the performance of the GPC algorithm [15] on our data, and found that comparable good performance was observed when learning from the synthetic data, although it tends to need twice as much data to achieve similar accuracy, and comparison can only be done on the final network. It specifically learns a local Bayesian network, and appears to be used in [15] to add a neighbourhood of radius up to 2 around the single seed gene. The model appears to grow without refinement - once an edge is determined to be present or absent, it is not re-evaluated later in light of other dependencies in the new neighbourhood, thus we found that very different networks were learned from different starting genes with the GPC algorithm on real data, giving little confidence in the method once away from local dependencies (for which it is good). Inspecting final networks also showed that the clock genes were not highly connected, as we would expect - for example, one example result of our method illustrated in Figure 5, shows that the 13 clock genes highlighted in light green have 25 connections between them, and 17 connections to non-clock genes, whereas in a network grown using Pena's GPC algorithm, the 13 genes had only 9 connections between them, and over 60 to non-clock genes.

In recent years, computational modelling of the circadian clock has been performed to include properties such as protein levels, protein localization and light inputs as well as transcript levels [18-20,28]. The approaches reported here do not rival this intensive modelling. Instead we use the clock as one example where there is sufficient literature to allow assessment of the accuracy of our model predictions. Using transcriptomic datasets it is not possible to model post-transcriptional processes explicitly. However, where light-dependent transcription factor degradation occurs, for example, this will produce effects on downstream transcript levels which we can model, thus capturing post-transcriptional processes implicitly.

## Conclusion

The approaches reported here offer a generic approach to modelling an extensive set of genes with the complementary goal of generating experimentally-testable hypotheses about the networks to which poorly-characterized genes may contribute. Significantly, large bodies of microarray data are becoming available for crop species such as barley, wheat, potato and tomato where it may be difficult to perform the genetic perturbations necessary to generate the best data for modelling. Analysis of these data for crop species will require methods which have moved beyond synthetic data and (often unicellular) model organisms. In addition, researchers will want access to tools giving them information and predictions specifically for the genes they are interested in, avoiding idiosyncratic differences between organisms and the problems of identifying *bonafide *orthologs between model and crop organisms.

## Methods

### Data

The NASC array database [2] contained data for 2,904 arrays when this research began and this is what we have used in our analyses. These are derived from samples from a range of different plant organs and many different environmental conditions and treatments capturing a large proportion of the *Arabidopsis *gene expression repertoire. This contains experiments including time series and perturbations, however, each microarray is treated as independent, and no explicit use of the time series, mutations or perturbation experiments conducted is made. MAS5.0 summarization is provided by default by NASC arrays and was used for convenience in this study. Recently other normalization methods have emerged (see [40] for a comparison) that outperform MAS5.0. However, the fact that these data are subsequently coarsely discretized into three classes will remove most of the differences between these approaches, particularly given the fact that potential differences are more likely for consistently low expression genes which are excluded from our analysis by an expression filter, detailed in the next section.

### Data quantization and filtering

The gene expression signal values are quantized into three classes (denoted LOW/MED/HIGH) on a per gene basis, since: (i) genes are expressed in different quantities - when some genes are most expressed, they still have low signal values when compared to other more abundant genes; (ii) this allows the data to be split into three equal sized classes (equal probability mass). This ensures that all quantized genes have very similar entropy, which is important in the model selection phase later in the incremental network learning algorithm. Figure 2 shows example gene expression histograms and decision boundaries for four clock genes, and Figure 4 shows the quantized gene expression profiles of the genes analysed in the second section of Results. In other works, differential expression analysis of data from specifically designed experiments (condition against a control) have been used to create under-expressed, normal, and over-expressed classes for each gene [3], however, we have a large compendium of array data taken from many different conditions, that cannot be processed in this way, and to avoid selection bias later we quantise each gene into three equal sized classes. Pe'er *et al*. [41] fit a mixture of Gaussians to the expression signals for each gene using the assumption the gene is in one of a few discrete functional expression states, which relates to its activity, with each mixture component relating to a state or class. However, analysis of the expression profiles (e.g. Figure 2) does not lead to any natural splits in our expression profile data that indicate the presence of any natural clusters in the data linked to the active state of the gene, hence we have not tried to cluster the signals, and having different number of classes for different genes would introduce selection bias at the incremental growing of networks stage of our algorithm. Methods that employ a predictive discretization procedure as part of the model selection process (through jointly optimising a discretization policy and the model structure) have been devised [42], and show that the discretization policy does effect the resulting networks inferred, however we do not currently choose to try this, due to the added level of complexity involved, and our main desire to add in genes to a network from a large set of (potentially thousands) of possible genes, which would make this approach infeasible.

Three classes are chosen for a simple reason: it is the smallest number for a discrete number of classes which allows non-linear relationships to be captured. It is anticipated that little extra information would be captured from four or more classes, and the number of free parameters in the model would become too large. With three classes the categories LOW and HIGH are separated by a MED class, whereas with only two classes expression levels close to the decision boundary may be misclassified.

An expression distribution filter is also used to remove those genes (actually, probe sets) whose decision class boundaries are within 10 units (raw signal values; see Figure 2) of each other, since the lack of a significant difference between LOW and HIGH expression values for a gene may indicate a lack of true signal in the data. This ensures that the expression levels for belonging to the LOW class and HIGH class are different enough not just for the classification to be down to measurement noise. A signal value of less than 20 is often regarded as background noise, when the gene is actually off. The expression distribution filter is a low entropy filter on the continuous expression data - removing genes with little variability in their signal values; this inherently filters out genes of overall low expression. Generally if a gene always has a low expression level, then it will be easily predictable (always low/off), and it is of no use to any model. This filtering step reduces the set of genes from 22,815 to 15,172. In practice this removes low entropy genes for which around two-thirds of the signals values are less than 20 (off/background noise).

As an example of a gene excluded from analysis by the expression distribution filter, we wished to include *GATA9 *(a duplicated version of *GATA12*) as a *GATA *factor of interest, however in order to quantize the gene expression signal values into three equally sized classes, thresholds of 12.56 and 21.01 would be necessary. With a difference in thresholds of less than 10 units, this gene exhibits a consistently low signal and low variance and is excluded.

### Bayesian networks for learning GRNs

The gene regulatory network is modelled with a discrete static Bayesian network (for an introduction to Bayesian networks see [43]). Our aim here is to learn the model structure *S *for the Bayesian network. The model structure is defined by a directed acyclic graph (DAG) encoding the dependencies between the variables (genes). The method aims to learn the model which is most likely to have generated the quantized gene expression data, *D*. We could choose to calculate the marginal likelihood p(*D*|*S*^*h*^) for each model structure *S*^*h*^, but for reasons of efficiency, an approximate BIC score is calculated as , where  is the maximum likelihood estimate of the model parameters for a model with structure *S*^*h*^, *d *is the number of parameters in the model, and *N *is the size of the dataset. In practice, the BIC score tends to score DAGs with fewer edges relatively more highly than marginal likelihood. No prior on model structures is used. A greedy hill climbing search where edges are added, reversed or deleted at each iteration is used until an optimum is reached. Fifty restarts (from random initial DAGs) are performed in order to avoid local optima.

### Incremental growing of networks

Starting from a set of genes known to be involved in the biological process of interest the Bayesian network learning algorithm detailed above may be used to find a gene network that was most likely to have generated the observed gene expression patterns. On each iteration, each possible gene from an extended set of genes (either a selection made by an expert from the literature, or the full set of all possible genes) is added separately to the set of genes in the current model (see Figure 1 for an overview and below for a formal algorithmic description). For each of these sets, the 'best' network is learned from the training data, as described in the previous section. The scores are compared, and the network with the highest score is accepted as the model at this stage (see Figure 7 for an example plot of the distribution of these scores). This model comparison or model selection phase is a notoriously hard and unsolved problem in the machine learning community. However, through the quantization steps outlined above, the entropy of all the sets of genes under consideration at any iteration is the same, thus removing any bias from the model selection task. This gives an order in which to add genes to the model; ideally this provides information about which genes are more likely than other genes to be explained by the inferred network, and therefore related to the initial genes. This procedure can be described algorithmically as follows:

1. Determine the set G of all possible probes for inclusion into the network (representing all 15,000+ genes, or an extended selection identified as possibly of interest), G = {*g*_j_: j = 1,…,N_g_}, a set of initial genes of interest s_0_, and decide how big a network to grow, setting max_net_size = min(N_g_, some limit).

2. At each iteration i, let *s*_i_ denote the best gene set, and for each gene *g* ∈ G \ *s*_i-1_ form a candidate gene set *s*_i_’ = *s*_i-1_ ∪ *g* from which to learn a Bayesian network structure  that maximises the BIC score for the gene set *s*_i_’. If BIC(*S*_i_’) > best_score then update it and save the best set and structure to *s*_i_ = *s*_i_’, and *S*_i_ = *S*_i_’ respectively.

3. Repeat step 2, incrementing *i* whilst |s_*i*_| < max_net_size and resetting best_score to -∞ at the start of each iteration.

Generally, it is not possible to compare network structures on different data sets, since the likelihood term p(*D*|*S*^*h*^, ) is data dependent. This section aims to provide some detail to explain how the quantization steps undertaken allow model comparison between networks formed from different gene sets. The likelihood is decomposable as a product of the individual observations x ∈ *D*. So, p(D|S) = ∏p(x|S) for each observation x. In our case we have *N *= 2904 microarray observations, and x covers the current selection of genes. Each p(x|S) is also decomposable as a product of the conditional probabilities for each variable (gene) given its parents p(x|S) = ∏p(*x*_*i*_|*parents(x*_*i*_*)*). So the contribution from an unconnected gene *g *is ∏p(*g*) over all observations, which is (1/3)^*N*^. (*N *observations, with a probability of 1/3 of being correct in each case, since the classes are of equal size). This contributes *N *ln(1/3) to the log likelihood. Owing to the quantization, any unconnected gene contributes the same to the likelihood. To perform direct comparison of the structure *S*_A _learned from *s*_*i *_∪ *g*_A _and *S*_B _learned from *s*_*i *_∪ *g*_B _we would need to add gene *g*_B _unconnected to *S*_A _and *g*_A _unconnected to *S*_B_. However, due to quantization *g*_A _and *g*_B _when not connected to any other genes in the model would contribute the same to the likelihood (and to the penalization term of the BIC score). Thus, these terms cancel and we can effectively compare the scores BIC(*S*_A_) vs BIC(*S*_B_). The choice of quantization to ensure each gene has the same entropy has allowed model comparison for networks of the same size that differ by a single gene.

Iteratively learning networks exhaustively from a full set of 15,000+ genes is possible, but computationally time consuming. This scheme is embarrassingly parallel, and speed-up close to linear with the number of processors used is achieved. Future work may investigate exploiting cheap mutual information based measures to select a set of genes for possible inclusion into the expensive network learning stage.

The main contribution of this algorithm is in the incremental addition of genes from a large (potentially whole genome) set. Thus its purpose is to identify a network that incorporates the initial key genes of interest and to form a hypothesis of how they fit into a larger network - one that is statistically likely to have generated the observed data. This is done in a greedy manner to make the problem tractable. If we consider adding *k *genes from a set of *N *possible genes, then it requires creating less than *kN *sets from which to learn networks: first *N *sets each with a new gene are created, the best chosen, and a new *N*-1 sets created and the best chosen, so the actual number of sets is *k*(*N*-1/2(*k*+1)). An exhaustive search requires all _*N*_C_*k *_possible sets to be created and evaluated. For the interesting scenarios, *k *<<*N *and *N*!/(*N*-*k*)!*k*! ≈ *N*^*k*^. So the speed up obtained (without optimization) is of the order *kN *vs *N*^*k*^. With *N *= 10000 and *k *= 10, this gives a speed up of 10^35^, making the problem tractable. In order to demonstrate the trade off between speed and accuracy using this approach, we consider a small scale example as an illustration, starting from the second example in Results: from the 9 key genes, an exhaustive search of all possible combinations of three genes from the remaining 28 are used to learn networks of 12 genes (this gives 3276 sets), and a score is obtained for each. On this small scale example, the greedy search involves learning networks on 81 sets of genes. Genes are added in by our greedy search in the order: COL2, COL1, PRR5, (then LUX, and PRR7). All the top scoring networks in the exhaustive search contain both genes COL1 and COL2, and with a third gene from {PRR5, CRY1, PRR7, LUX} all with similar good scores. In three sets of runs of the exhaustive learning, each with 50 restarts of the greedy algorithm for learning networks (within the greedy search for iterative addition of genes) a small variation of the best scores for learned networks was observed, as a global optima was not reached in all cases (there are more than 10^25 ^possible DAGs on 12 nodes, so this is not that surprising). The quality of the order the genes are added in appears to be good (and is subject to noise in network learning, rather than in gene addition order, as if a gene is missed from the order, it is likely to be added at the next iteration anyway). A statistically rigorous stopping criterion has not yet been devised. This has not been a focus, since generally as the networks grow, large changes in the networks do not occur and more genes are added at the periphery, with much of the network the same from one iteration to the next; generally 3 or 4 edges change when each gene is added, and 2 or 3 of these tend to be to or from the added gene. So when to stop is not a critical issue. Currently a practical size of twenty-something is used when learning from the whole genome, or the whole set for small sets of genes (up to 40).

The networks learned will be related to the initial genes. Starting with a set of genes involved in a specific process genes involved in a more general global regulation of expression (such as circadian clock) may be incorporated, conversely, starting from a set of genes involved in a general global regulation of expression, it would be unlikely that the same set of specific genes would be added, other more general global regulation genes would be added first. As the iterations go on genes less related to the initial genes will be added. Indeed, different networks will be learned when iteratively adding genes from different lists (c.f. Figures 5 &6). The algorithm finds the statistically best explanation from a given gene list. Thus, the network in Figure 5 represents a good network that was most likely to have generated the expression data for those genes, whereas, network in Figure 6 adds genes from the whole genome and does not contain links between other groups of genes (i.e. cold/salt stress). Thus we can constrain lists to find a network that captures the relationships between these genes, or identify a network incorporating genes from an unconstrained (whole genome) list.

### Conditional independence, Markov equivalence, CPDAGs & CPTs

The learned network structures encode the conditional independence relations between the variables (genes). The resulting Bayesian network is represented by a DAG and the corresponding conditional probability distributions (parameter tables). However, there are a number of structures which are Markov equivalent and this set of equivalent DAGs can be represented as a Completed Partially Directed Acyclic Graph (CPDAG) where the directionality of the regulatory relationship is only indicated where a causal relation can be inferred. Specifically, where there is strong enough evidence that a v-structure is formed, i.e. a variable is dependent on more than one other variable. Thus, rather than just capturing correlations between genes, a predictive model, which is most likely to have generated the observed data is formed. The learned network models shown in Figures 3, 5 &6 are CPDAGs which represent an equivalence class of DAGs with the same predictive ability or that are equally likely to have generated the observed data.

### Generation of synthetic data for performance evaluation

In order to perform quantitative evaluation of our algorithms, the underlying ground truth network must be known. Thus, we construct a network, and generate data from it. We can then try to recover the original network from the data. To investigate the influence of the underlying network structure on the learning algorithm we wish to be able to control the strength of the relationships, rather than just using random CPDs which may by chance be strong or very weak. In the real microarray data, each gene's expression values are quantised into 3 equal sized classes and this is very important in the model comparison step of the iterative growing of the networks. Our discrete synthetic data should have the same property.

The CPTs represent a multinomial distribution, which has the Dirichlet distribution as its conjugate prior. Therefore, we are able to generate data to control the strength of relationships between variables. If each row in a CPT ~ Dir(α_1_, α_2_, α_3_) then the alpha parameters can be used to control the strength of the relationships. When the α's are equal to 1, then each probability in the CPT is randomly drawn from U(0,1) then normalised. For α_i _>> 1, the distribution tends to uniform (1/3, 1/3, 1/3) (and has a high entropy, since this distribution is not predictive); and for α_i _<< 1 to a deterministic relationship with one state having all the probability mass. Inspection of the CPTs learned from data empirically shows that they are far from fully deterministic, but more correlated than random and certainly not uniform (information-less), so alpha parameters of less than one would be expected. From the learned network on 35 genes (see Results) data from the learned CPTs was used to estimate the parameters of a Dirichlet distribution [44], revealing α = (0.6, 0.9, 0.6) as the maximum likelihood estimate of the parameters. The rows of the learned CPTs show that the middle entry (MED) has smallest variance, rarely taking tiny or large probabilities, compared to the other two states (LOW and HIGH) - the MED class separates the LOW and HIGH expression values and captures some noise in the data, and we would inherently expect this state to be more uniform than the other two.

For each gene, the probability of it being in a particular state given a particular configuration of parent's states is drawn from a Dirichlet distribution (with α = (0.6, 0.9, 0.6)). α controls the entropy of the dirichlet sampling. The expected frequencies of parent states for each gene are taken into account during the allocation of the CPTs, in order to maintain roughly equal frequencies of the states of each node.

When this model (the Bayesian network with CPTs) is sampled from, a dataset can be formed, from which we can reverse engineer the known network. We perform network inference from different sized data sets (by truncating the largest one). Network structures are learned as described previously, again starting from nodes corresponding to the same 9 key genes as before. Networks are learned on datasets of size 10, 100, 500, 1000, 2000, 3000, 5000, 10000 to demonstrate behaviour on different size datasets.

### Evaluation metrics

The network structures are evaluated based on counts of correctly and incorrectly inferred edges. Given that a number of DAGs are Markov equivalent, the CPDAGs of each DAG will be used when a learned DAG is compared to the underlying DAG. For each predicted edge, it is counted as a FP if the corresponding edge in the original network is not present or if the predicted edge is compelled in one direction and the corresponding edge in the original network is compelled in the opposite direction; otherwise the edge is counted as a TP. For each predicted non-edge, it is counted as TN if the corresponding edge in the original network was also not present; otherwise it is counted as a FN. Note the comparison is done against the whole network, even when our model only includes a limited number of nodes, as the algorithm iteratively adds nodes.

## Authors' contributions

CJN is the primary author of the paper. IWM and PMG have provided biological interpretation and insight to the results. AJB and DRW have aided in the design and analysis of the algorithms. All authors read and approved the final manuscript.

## Supplementary Material

Additional file 1 **Mini-website showing all learned network graphs for examples presented**. Mini-website showing all learned network graphs at each iteration for the examples presented in the main body of the paper, and a table of the genes involved. Click here for file
