# Supplementary material for: From gene expression to gene regulatory networks in Arabidopsis thaliana
Source: BMC Syst Biol. 2009 Sep 3;3:85. doi: 10.1186/1752-0509-3-85 (PMC2760521; doi:10.1186/1752-0509-3-85)
Supplement: Additional file 1 — Mini-website showing all learned network graphs for examples presented. Mini-website showing all learned network graphs at each iteration for the examples presented in the main body of the paper, and a table of the genes involved. [file 1752-0509-3-85-S1.zip › S/index.html]

Supplementary Information


# From gene expression to gene regulatory networks in *Arabidopsis thaliana*

This mini-website contains Supplementary Information to accompany the paper.
Each set corresponds to the examples in the paper. Following a numbered link
will show an image of the network for the iteration with that number of genes
included in the model.
It must be noted that these networks show a learned DAG in each case. There
exists an equivalence class of CPDAGs, as discussed and demonstrated on examples
in the main paper.

### 1. The *Arabidopsis* circadian clock

4 |
5 |
6 |
7 |
8 |
9 |
10 |
11 |
12

### 2. Other networks and poorly characterised genes

10 |
11 |
12 |
13 |
14 |
15 |
16 |
17 |
18 |
19 |
20 |
21 |
22 |
23 |
24 |
  
25 |
26 |
27 |
28 |
29 |
30 |
31 |
32 |
33 |
34 |
35 |

### 3. From an unselected list of 15,000+ genes

10 |
11 |
12 |
13 |
14 |
15 |
16 |
17 |
18 |
19 |
20 |
21 |
22 |
22 (with gene symbols)

### 4. Evaluation on realistic synthetic data

10 |
11 |
12 |
13 |
14 |
15 |
16 |
17 |
18 |
19 |
20 |
21 |
22 |
23 |
24 |
  
25 |
26 |
27 |
28 |
29 |
30 |
31 |
32 |
33 |
34 |
35 |
36 |
37

With nodes fixed, to aid interpretation:

10 |
11 |
12 |
13 |
14 |
15 |
16 |
17 |
18 |
19 |
20 |
21 |
22 |
23 |
24 |
  
25 |
26 |
27 |
28 |
29 |
30 |
31 |
32 |
33 |
34 |
35 |
36 |
37

### 5. List of probes and genes used in the study

View table
