# Supplementary material for: From gene expression to gene regulatory networks in Arabidopsis thaliana
Source: BMC Syst Biol. 2009 Sep 3;3:85. doi: 10.1186/1752-0509-3-85 (PMC2760521; doi:10.1186/1752-0509-3-85)
Supplement: Additional file 1 — Mini-website showing all learned network graphs for examples presented. Mini-website showing all learned network graphs at each iteration for the examples presented in the main body of the paper, and a table of the genes involved. [file 1752-0509-3-85-S1.zip › S/table1.html]

Supplementary Information


### 5. List of probes and genes used in the study

| Gene symbols | AGI code | probe set |
| --- | --- | --- |
| CCA1 | AT2G46830 | 266719\_at |
| LHY | AT1G01060 | 261569\_at |
| TOC1 | AT5G61380 | 247525\_at |
| GI | AT1G22770 | 264211\_at |
| GATA22 | AT4G26150 | 254016\_at |
| GNC/GATA21 | AT5G56860 | 247980\_at |
| GATA2 | AT2G45050 | 266125\_at |
| GATA4 | AT3G60530 | 251373\_at |
| GATA12 | AT5G25830 | 246913\_at |
| COL2 | AT3G02380 | 258497\_at |
| COL1 | AT5G15850 | 246523\_at |
| ELF4 | AT2G40080 | 267364\_at |
| PRR5 | AT5G24470 | 249741\_at |
| LUX | AT3G46640 | 252475\_s\_at |
| PRR7 | AT5G02810 | 250971\_at |
| PRR9 | AT2G46790 | 266720\_s\_at |
| CRY1 | AT4G08920 | 255068\_at |
| HYH | AT3G17609 | 258349\_at |
| ELF3 | AT2G25930 | 266839\_at |
| PRR3 | AT5G60100 | 247668\_at |
| HY5 | AT5G11260 | 250420\_at |
| CBF3 | AT4G25480 | 254066\_at |
| PHYA | AT1G09570 | 264508\_at |
| CRY2 | AT1G04400 | 255068\_at |
| CKB3 | AT3G60250 | 251411\_at |
| CBF2 | AT4G25470 | 254075\_at |
| SRR1 | AT5G59560 | 247703\_at |
| ZAT10 | AT1G27730 | 261648\_at |
| ZAT12 | AT5G59820 | 247655\_at |
| RAV1 | AT1G13260 | 259364\_at |
| PHYE | AT4G18130 | 254680\_at |
| DET1 | AT4G10180 | 255799\_at |
| PHYB | AT2G18790 | 266065\_at |
| PIF3 | AT1G09530 | 264510\_at |
| ZTL | AT5G57360 | 247898\_at |
| PHYC | AT5G35840 | 249666\_at |
| PHYD | AT4G16250 | 245487\_at |
| GUN4 | AT3G59400 | 251519\_at |
| GUN5 | AT5G13630 | 250243\_at |
| AT5G64940 | AT5G64940 | 247232\_at |
| AT3G56940 | AT3G56940 | 251664\_at |
| AT1G74470 | AT1G74470 | 260236\_at |
| AT3G26570 | AT3G26570 | 257311\_at |
| LHCB6 | AT1G15820 | 259491\_at |
| PSBY | AT1G67740 | 245195\_at |
| AT3G26570 | AT4G22890 | 254298\_at |
| HPR | AT1G68010 | 260014\_at |
| PSAN | AT5G64040 | 247320\_at |
| PSBO2 | AT3G50820 | 252130\_at |
