# Supplementary figures and images for: From gene expression to gene regulatory networks in Arabidopsis thaliana (part 2 of 2)
Source: BMC Syst Biol. 2009 Sep 3;3:85. doi: 10.1186/1752-0509-3-85 (PMC2760521; doi:10.1186/1752-0509-3-85)

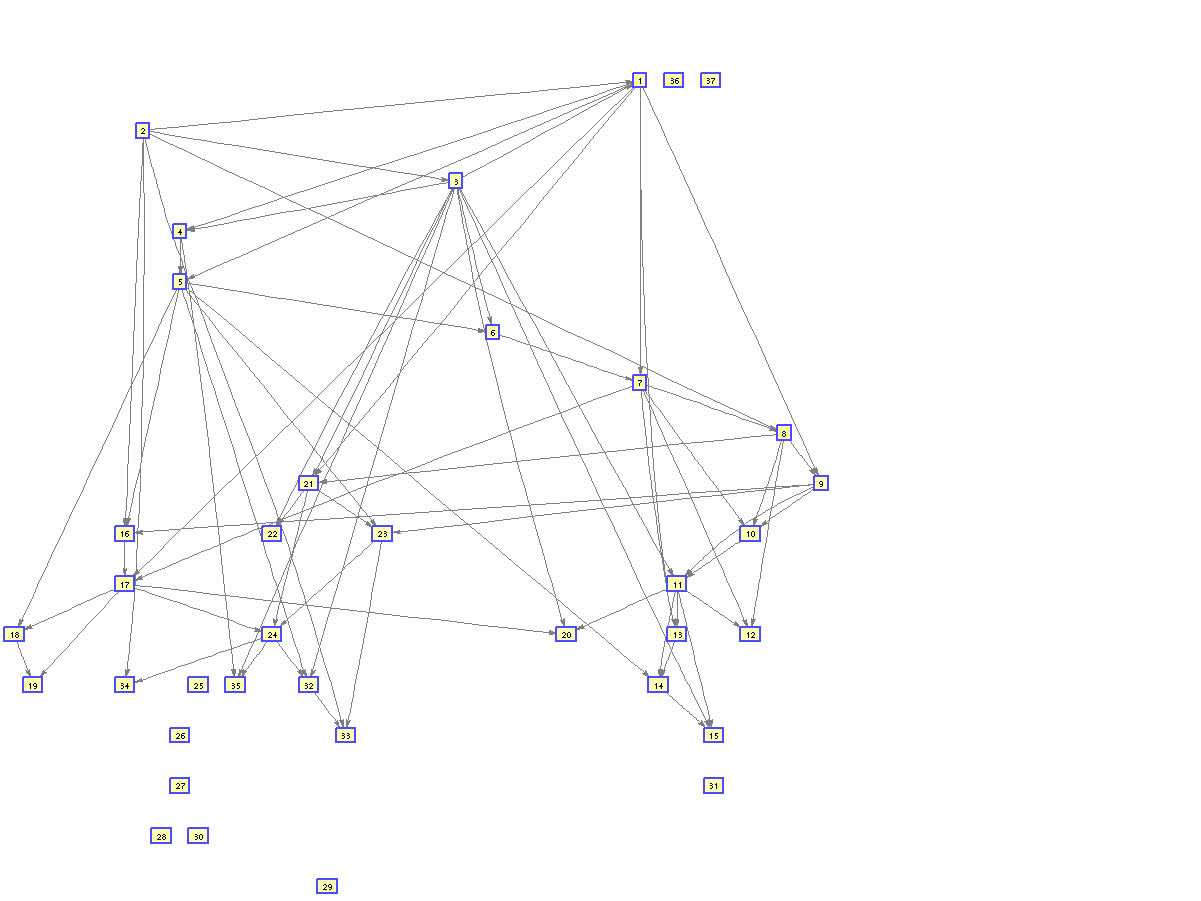

Supplement: Additional file 1 — Mini-website showing all learned network graphs for examples presented. Mini-website showing all learned network graphs at each iteration for the examples presented in the main body of the paper, and a table of the genes involved. [file 1752-0509-3-85-S1.zip › S/net4/grn_fixed_layout_28.png]

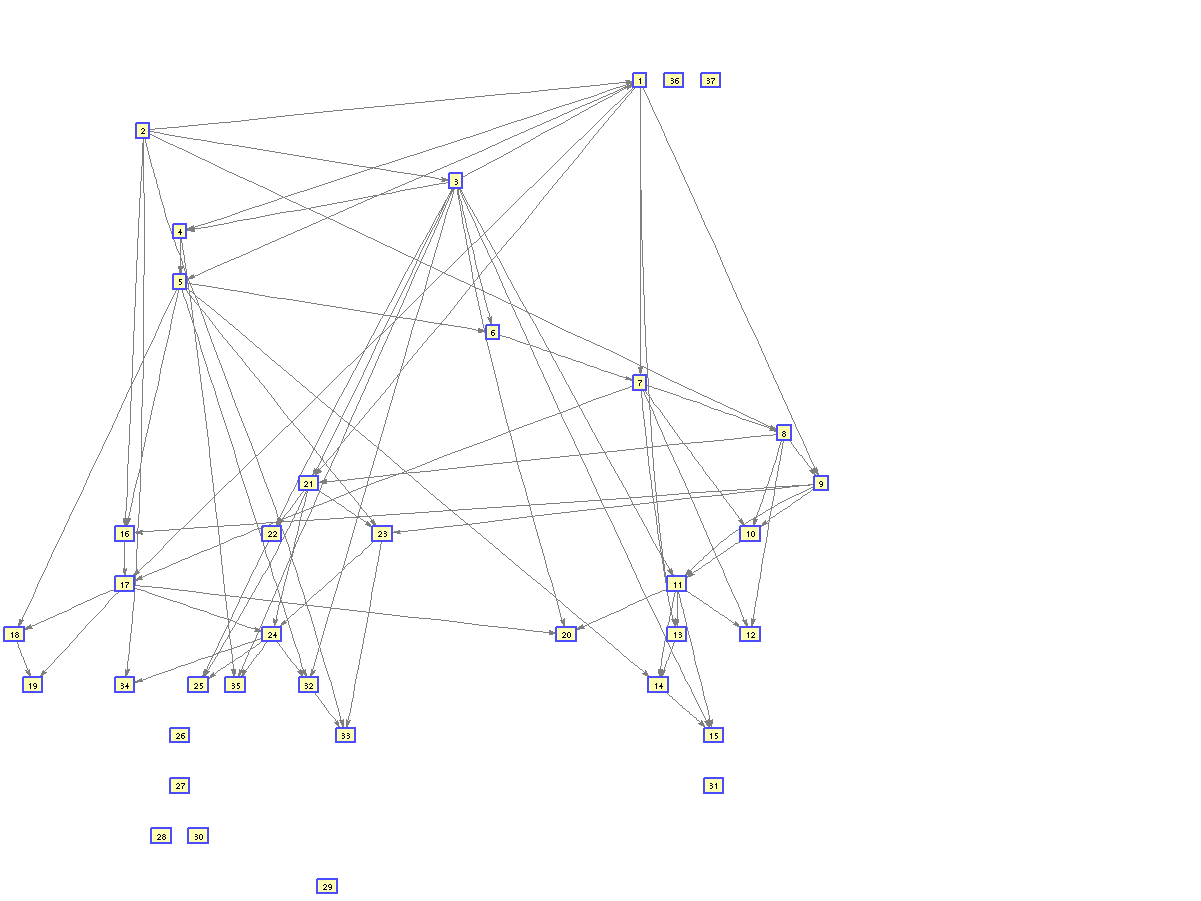

Supplement: Additional file 1 — Mini-website showing all learned network graphs for examples presented. Mini-website showing all learned network graphs at each iteration for the examples presented in the main body of the paper, and a table of the genes involved. [file 1752-0509-3-85-S1.zip › S/net4/grn_fixed_layout_29.png]

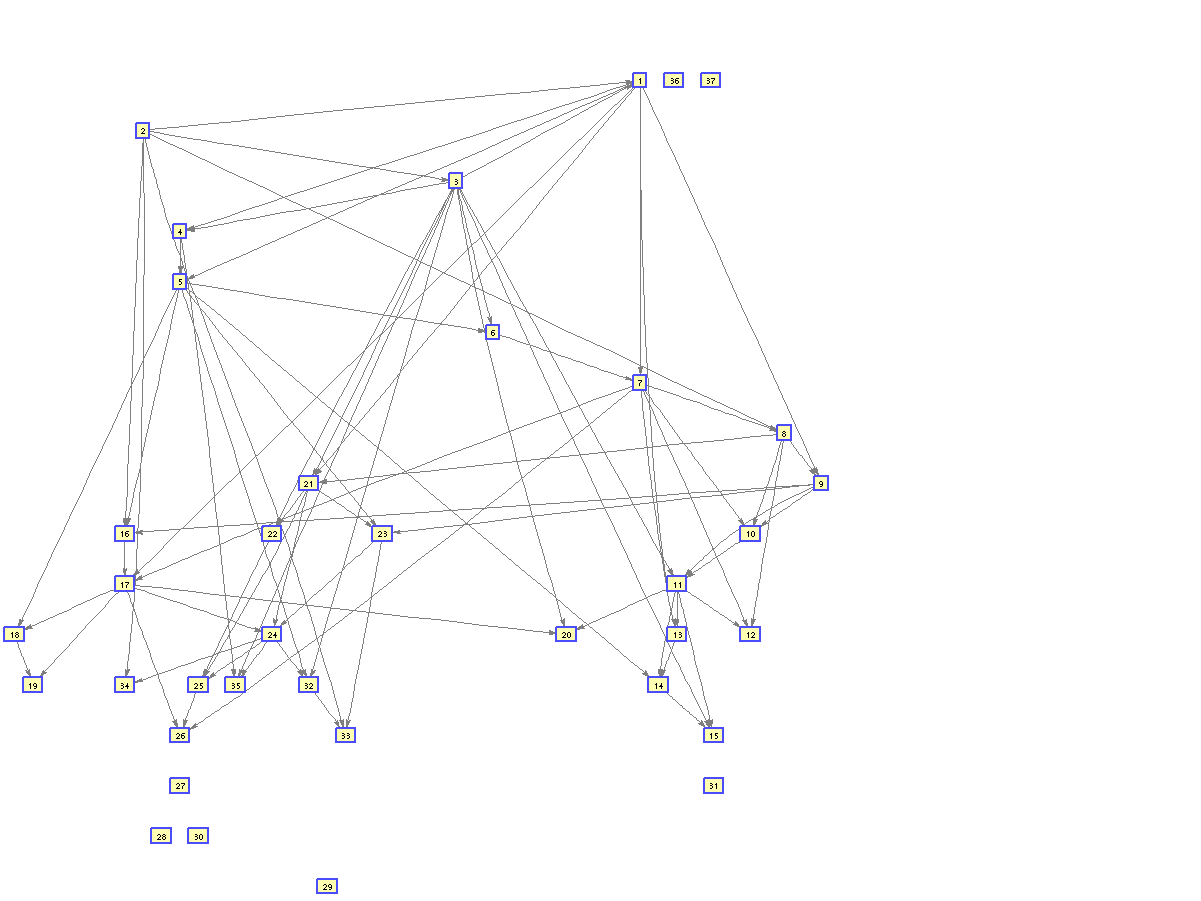

Supplement: Additional file 1 — Mini-website showing all learned network graphs for examples presented. Mini-website showing all learned network graphs at each iteration for the examples presented in the main body of the paper, and a table of the genes involved. [file 1752-0509-3-85-S1.zip › S/net4/grn_fixed_layout_30.png]

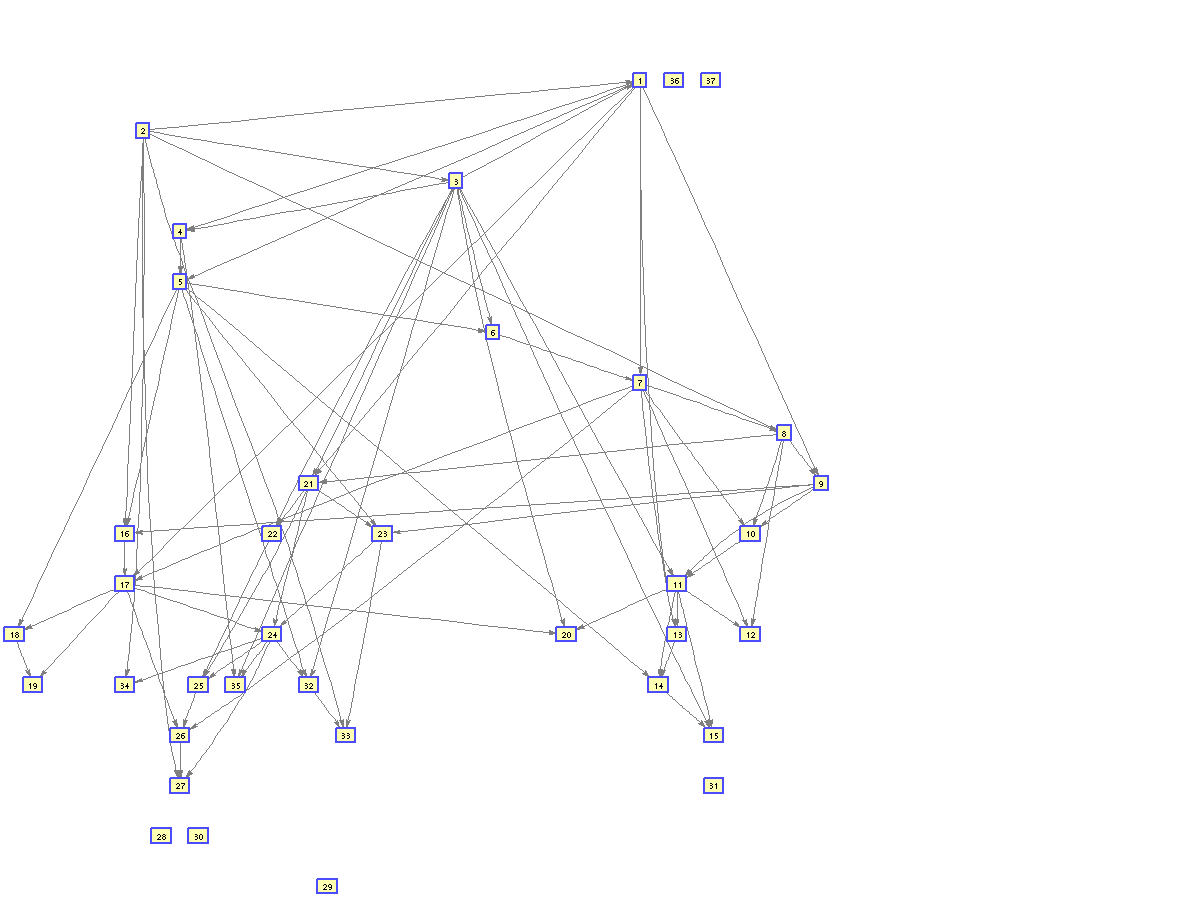

Supplement: Additional file 1 — Mini-website showing all learned network graphs for examples presented. Mini-website showing all learned network graphs at each iteration for the examples presented in the main body of the paper, and a table of the genes involved. [file 1752-0509-3-85-S1.zip › S/net4/grn_fixed_layout_31.png]

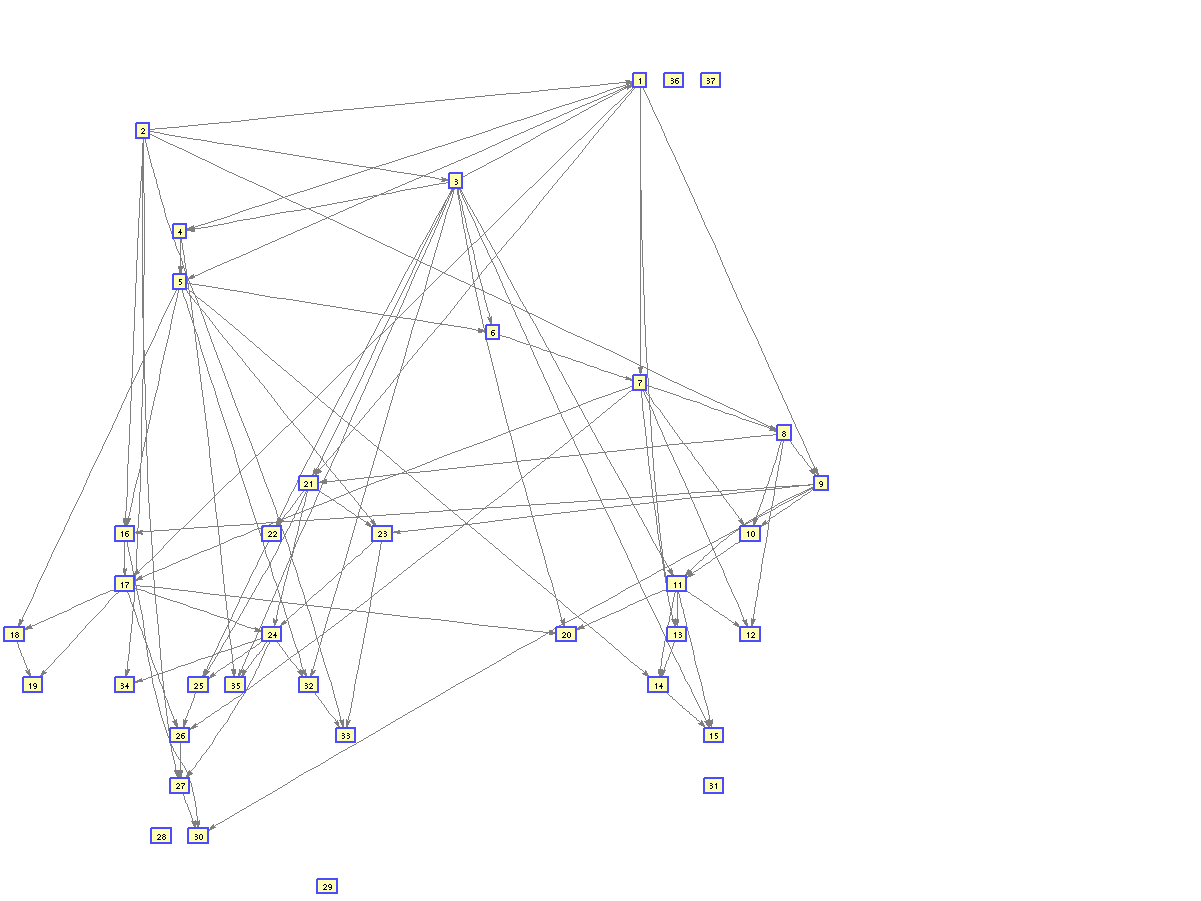

Supplement: Additional file 1 — Mini-website showing all learned network graphs for examples presented. Mini-website showing all learned network graphs at each iteration for the examples presented in the main body of the paper, and a table of the genes involved. [file 1752-0509-3-85-S1.zip › S/net4/grn_fixed_layout_32.png]

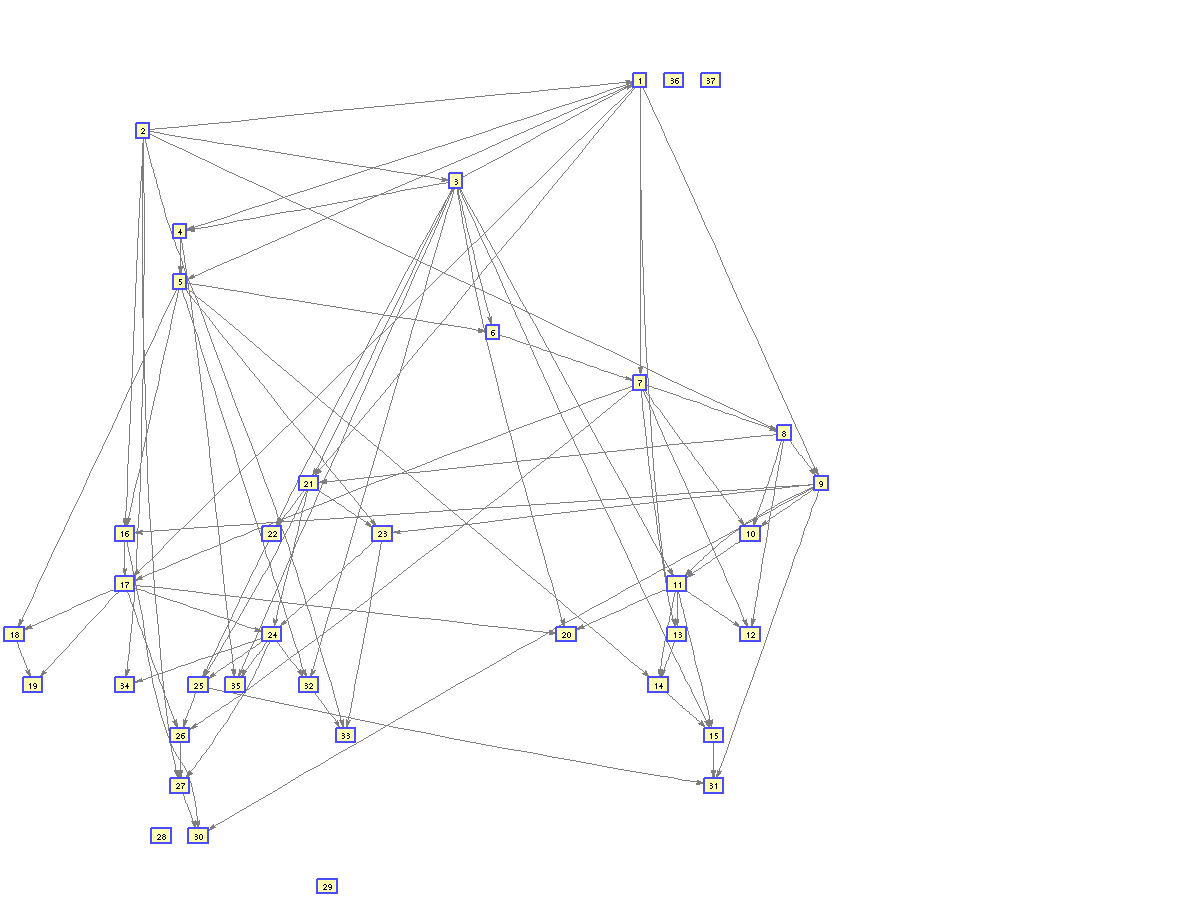

Supplement: Additional file 1 — Mini-website showing all learned network graphs for examples presented. Mini-website showing all learned network graphs at each iteration for the examples presented in the main body of the paper, and a table of the genes involved. [file 1752-0509-3-85-S1.zip › S/net4/grn_fixed_layout_33.png]

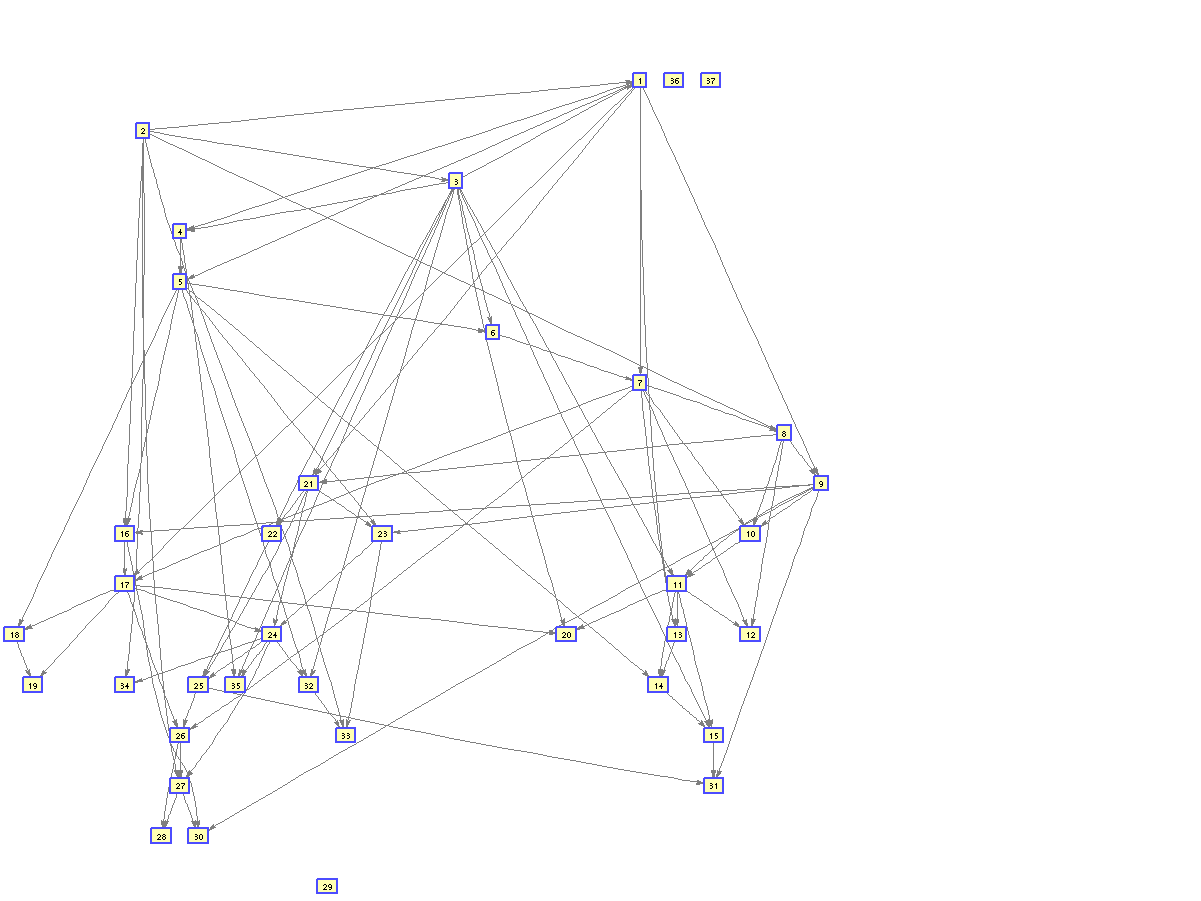

Supplement: Additional file 1 — Mini-website showing all learned network graphs for examples presented. Mini-website showing all learned network graphs at each iteration for the examples presented in the main body of the paper, and a table of the genes involved. [file 1752-0509-3-85-S1.zip › S/net4/grn_fixed_layout_34.png]

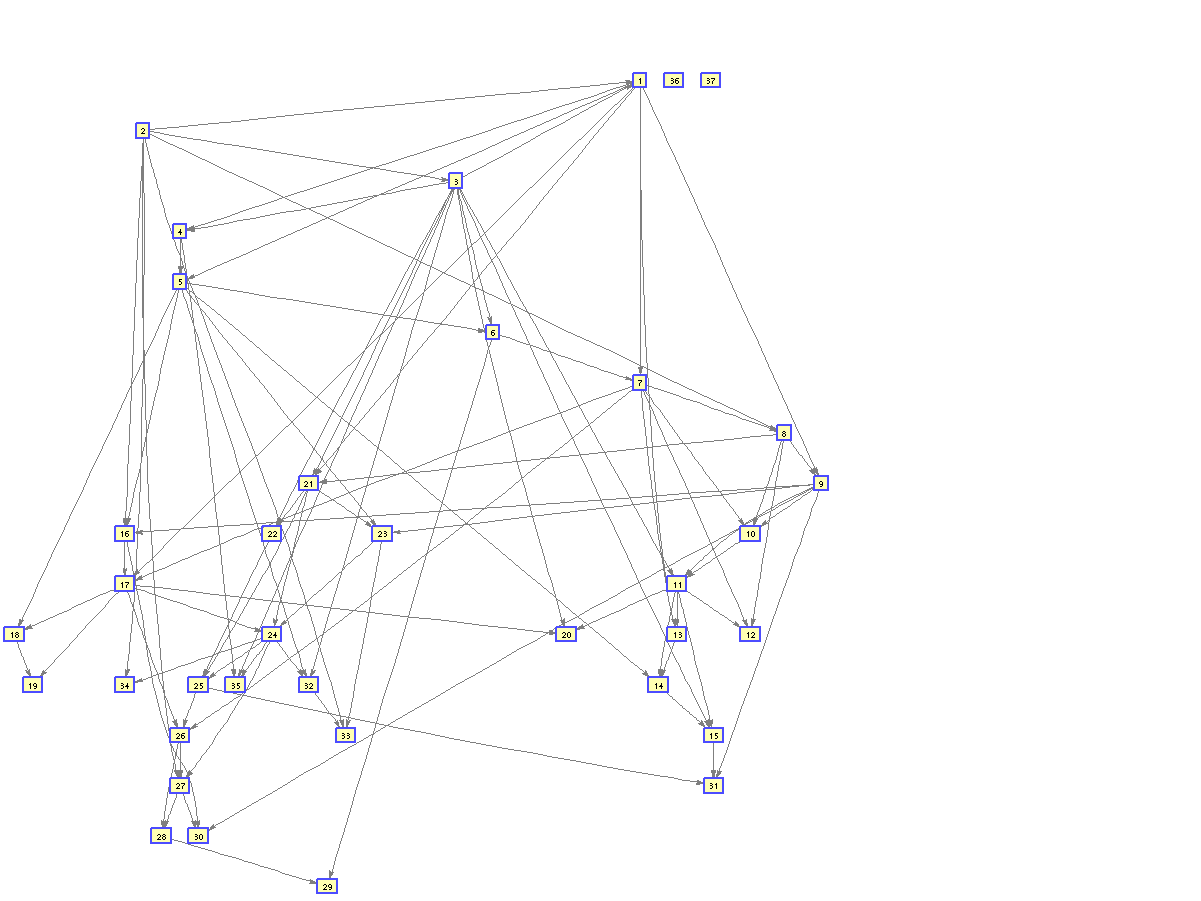

Supplement: Additional file 1 — Mini-website showing all learned network graphs for examples presented. Mini-website showing all learned network graphs at each iteration for the examples presented in the main body of the paper, and a table of the genes involved. [file 1752-0509-3-85-S1.zip › S/net4/grn_fixed_layout_35.png]

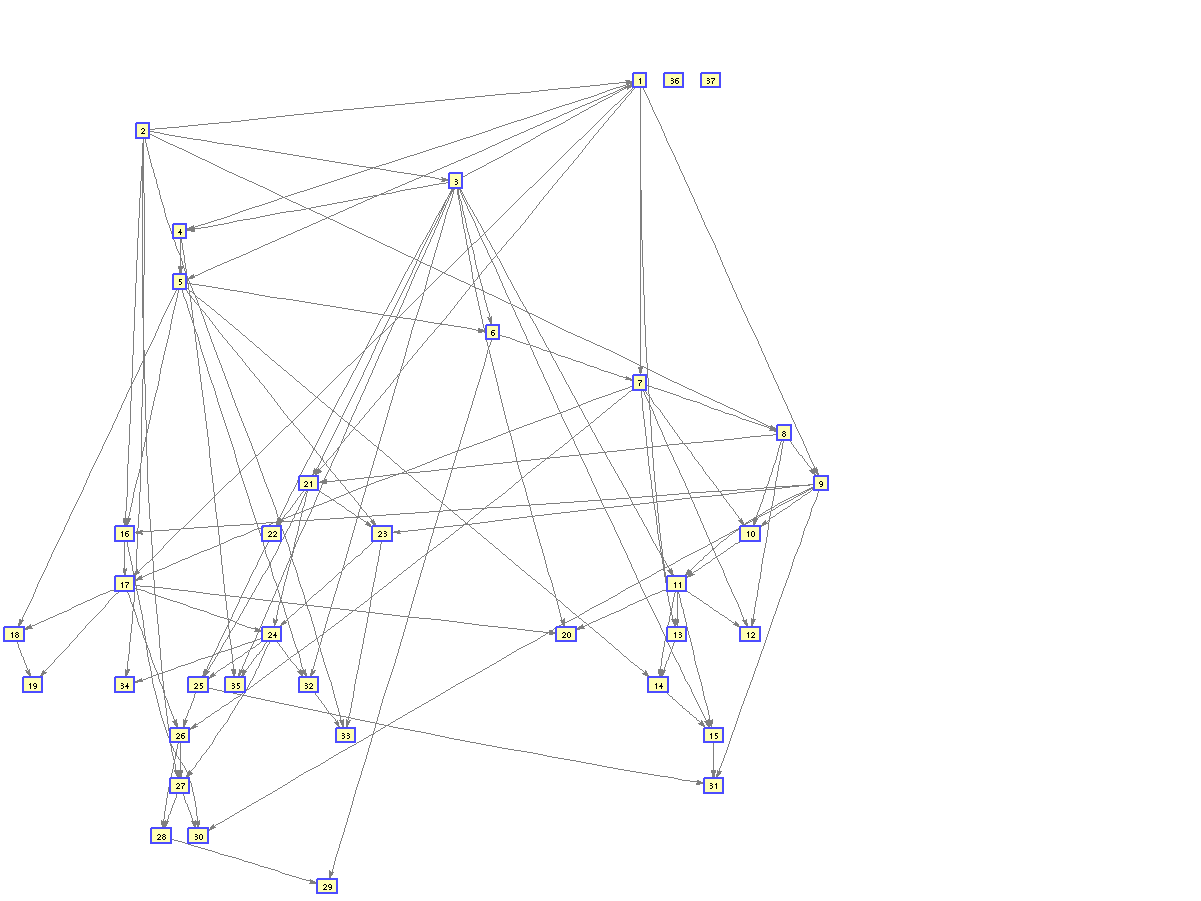

Supplement: Additional file 1 — Mini-website showing all learned network graphs for examples presented. Mini-website showing all learned network graphs at each iteration for the examples presented in the main body of the paper, and a table of the genes involved. [file 1752-0509-3-85-S1.zip › S/net4/grn_fixed_layout_36.png]

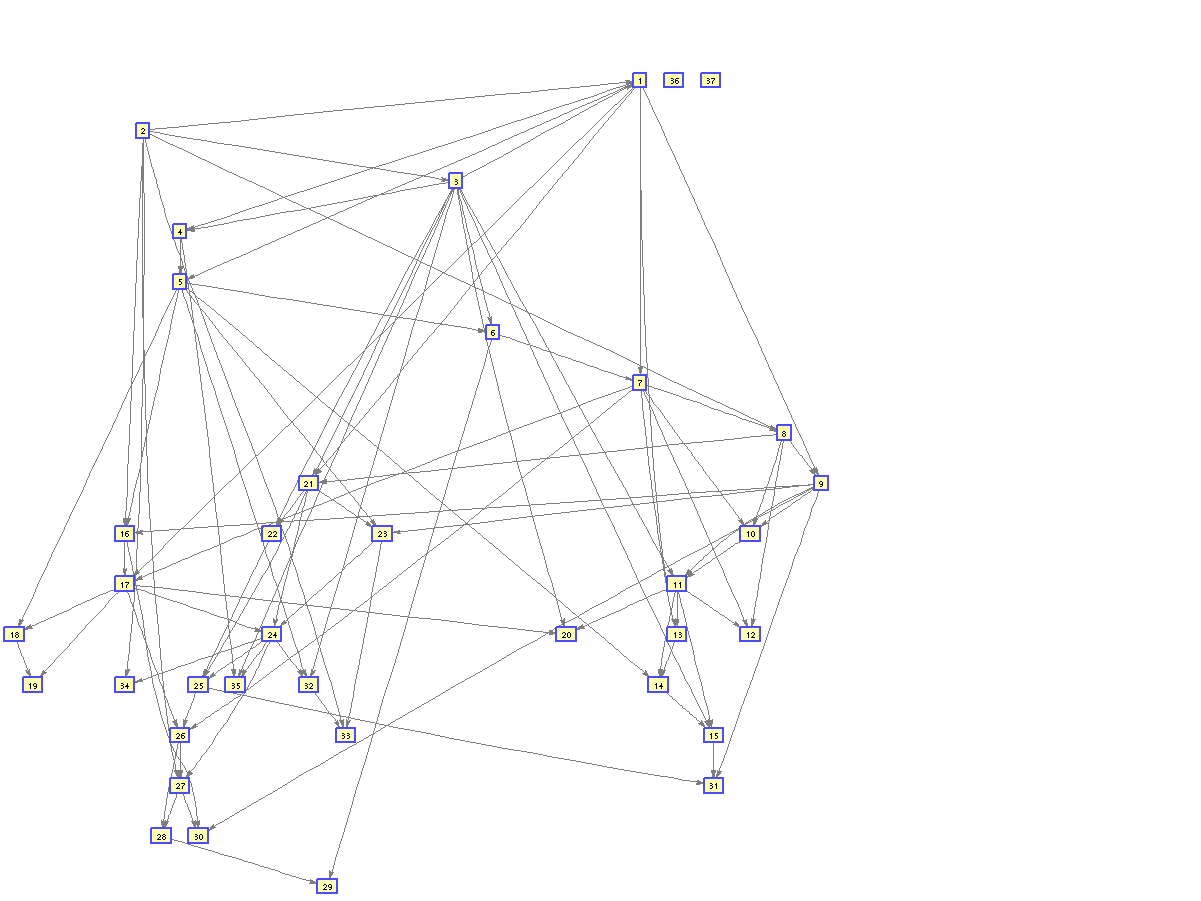

Supplement: Additional file 1 — Mini-website showing all learned network graphs for examples presented. Mini-website showing all learned network graphs at each iteration for the examples presented in the main body of the paper, and a table of the genes involved. [file 1752-0509-3-85-S1.zip › S/net4/grn_fixed_layout_37.png]
