# Supplementary figures and images for: From gene expression to gene regulatory networks in Arabidopsis thaliana (part 1 of 2)
Source: BMC Syst Biol. 2009 Sep 3;3:85. doi: 10.1186/1752-0509-3-85 (PMC2760521; doi:10.1186/1752-0509-3-85)

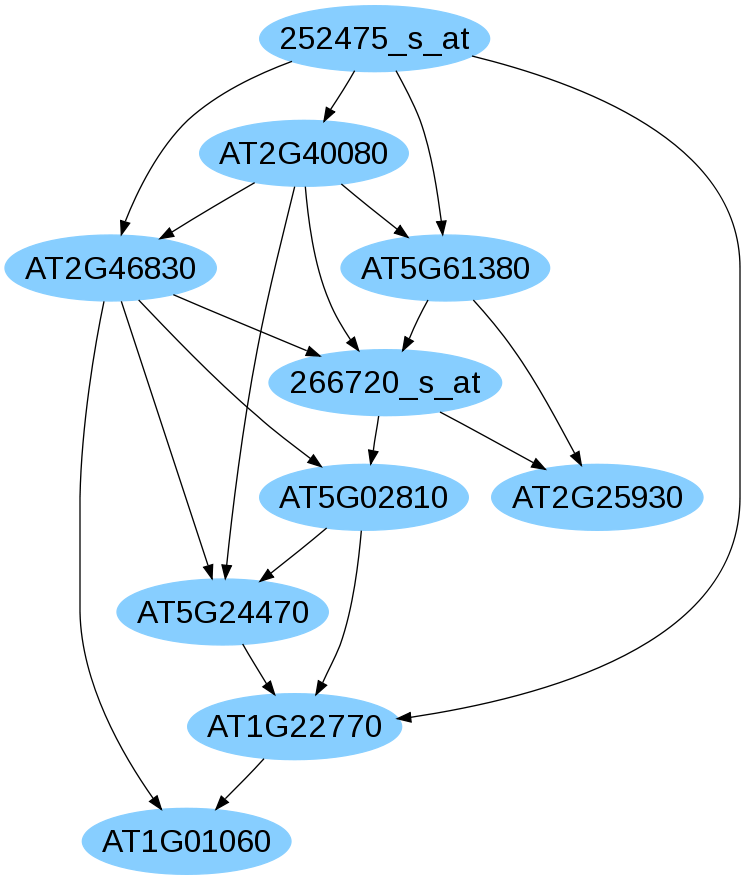

Supplement: Additional file 1 — Mini-website showing all learned network graphs for examples presented. Mini-website showing all learned network graphs at each iteration for the examples presented in the main body of the paper, and a table of the genes involved. [file 1752-0509-3-85-S1.zip › S/net1/grn_10.png]

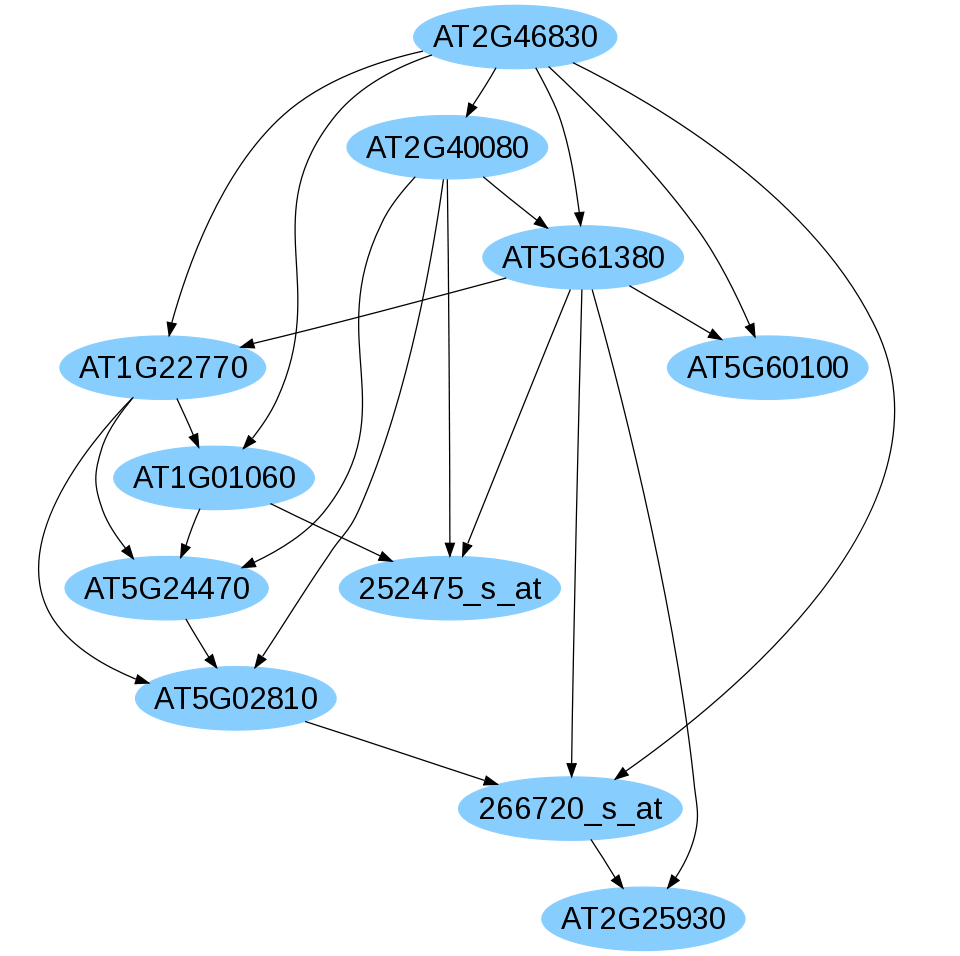

Supplement: Additional file 1 — Mini-website showing all learned network graphs for examples presented. Mini-website showing all learned network graphs at each iteration for the examples presented in the main body of the paper, and a table of the genes involved. [file 1752-0509-3-85-S1.zip › S/net1/grn_11.png]

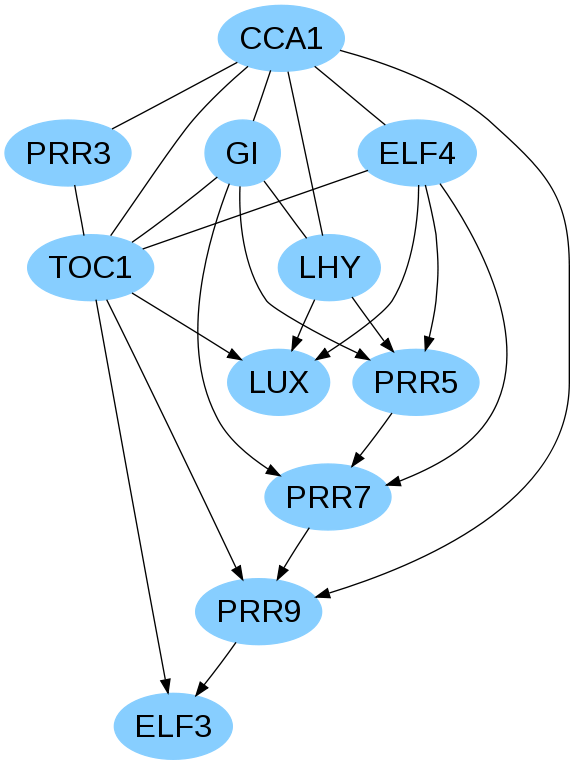

Supplement: Additional file 1 — Mini-website showing all learned network graphs for examples presented. Mini-website showing all learned network graphs at each iteration for the examples presented in the main body of the paper, and a table of the genes involved. [file 1752-0509-3-85-S1.zip › S/net1/grn11.png]

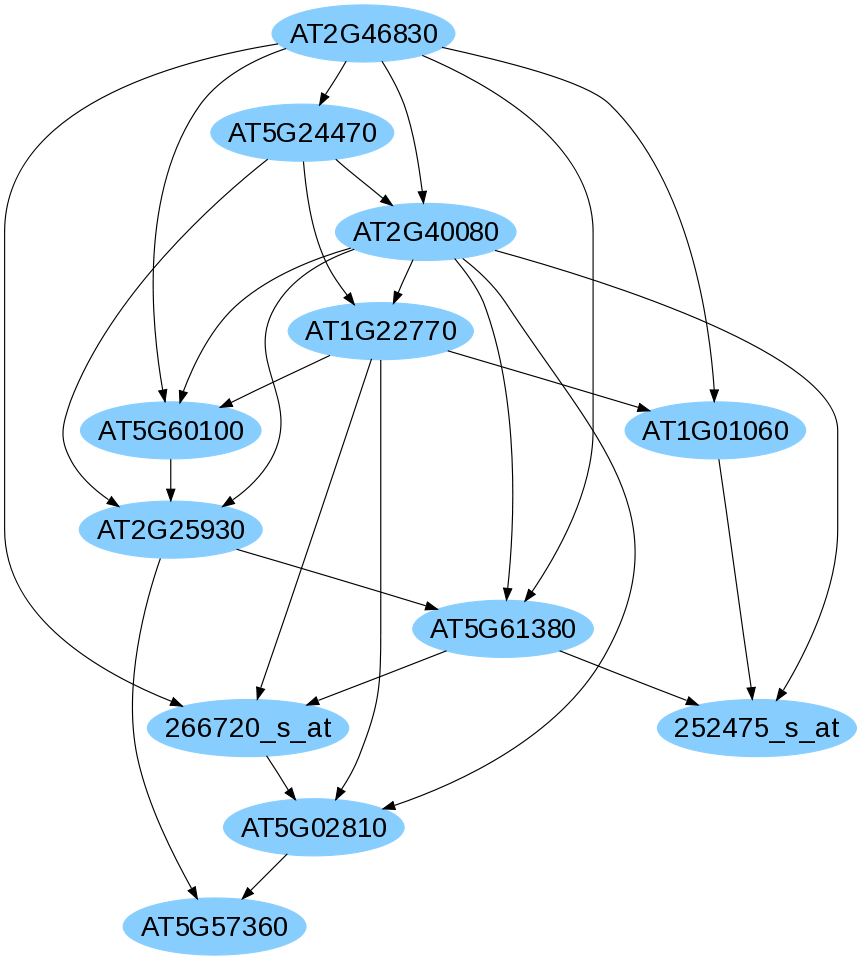

Supplement: Additional file 1 — Mini-website showing all learned network graphs for examples presented. Mini-website showing all learned network graphs at each iteration for the examples presented in the main body of the paper, and a table of the genes involved. [file 1752-0509-3-85-S1.zip › S/net1/grn_12.png]

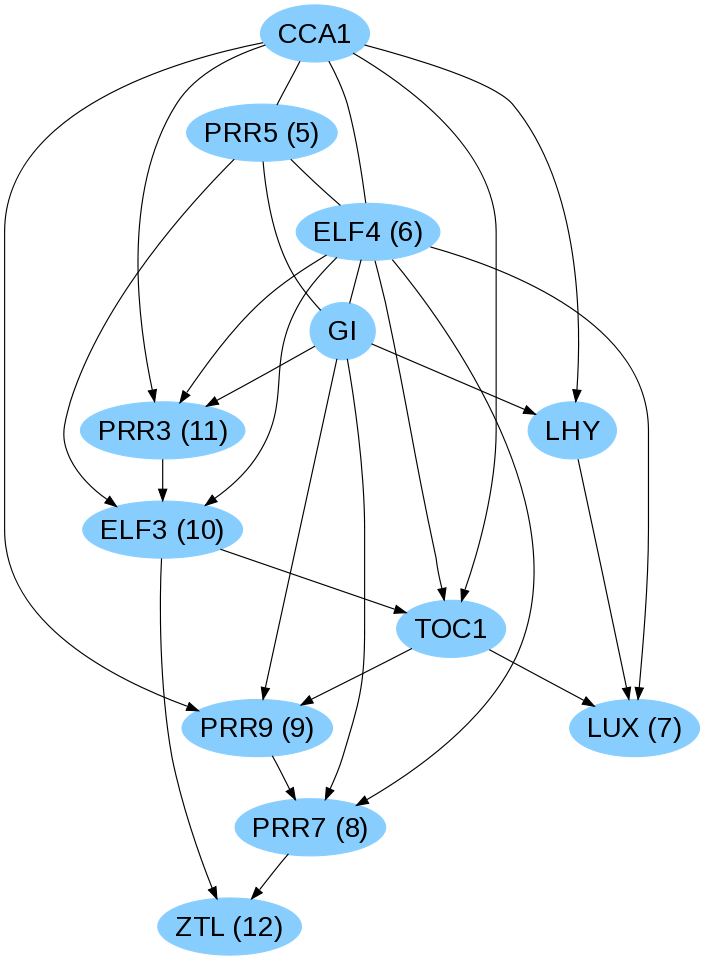

Supplement: Additional file 1 — Mini-website showing all learned network graphs for examples presented. Mini-website showing all learned network graphs at each iteration for the examples presented in the main body of the paper, and a table of the genes involved. [file 1752-0509-3-85-S1.zip › S/net1/grn12.png]

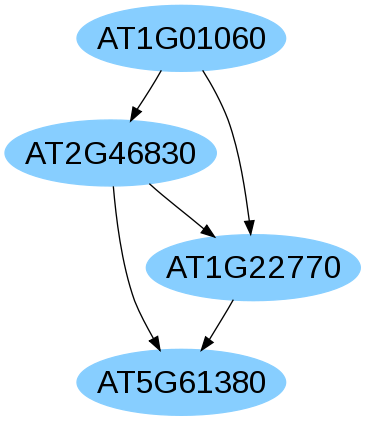

Supplement: Additional file 1 — Mini-website showing all learned network graphs for examples presented. Mini-website showing all learned network graphs at each iteration for the examples presented in the main body of the paper, and a table of the genes involved. [file 1752-0509-3-85-S1.zip › S/net1/grn_4.png]

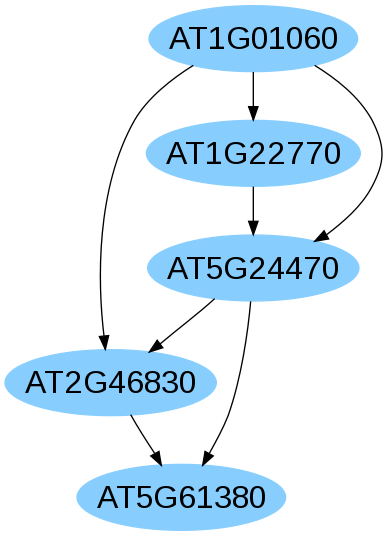

Supplement: Additional file 1 — Mini-website showing all learned network graphs for examples presented. Mini-website showing all learned network graphs at each iteration for the examples presented in the main body of the paper, and a table of the genes involved. [file 1752-0509-3-85-S1.zip › S/net1/grn_5.png]

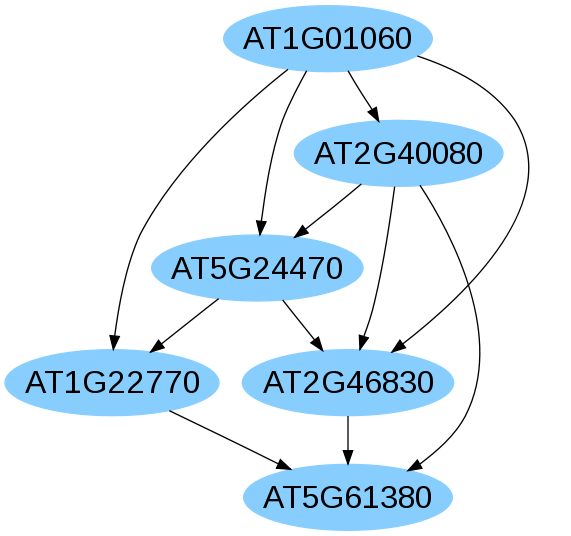

Supplement: Additional file 1 — Mini-website showing all learned network graphs for examples presented. Mini-website showing all learned network graphs at each iteration for the examples presented in the main body of the paper, and a table of the genes involved. [file 1752-0509-3-85-S1.zip › S/net1/grn_6.png]

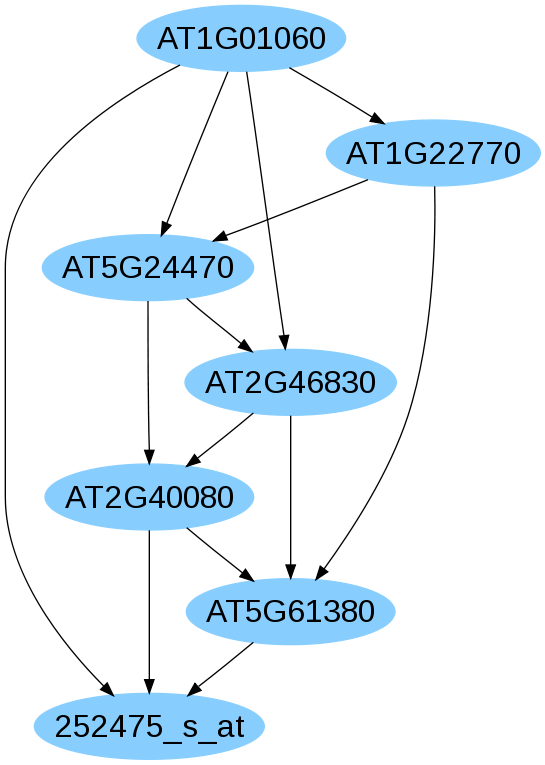

Supplement: Additional file 1 — Mini-website showing all learned network graphs for examples presented. Mini-website showing all learned network graphs at each iteration for the examples presented in the main body of the paper, and a table of the genes involved. [file 1752-0509-3-85-S1.zip › S/net1/grn_7.png]

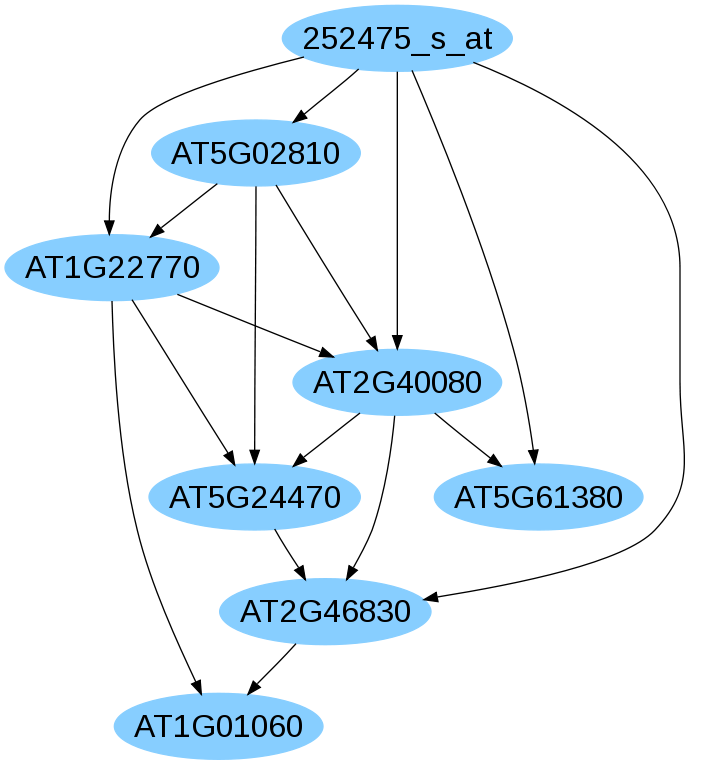

Supplement: Additional file 1 — Mini-website showing all learned network graphs for examples presented. Mini-website showing all learned network graphs at each iteration for the examples presented in the main body of the paper, and a table of the genes involved. [file 1752-0509-3-85-S1.zip › S/net1/grn_8.png]

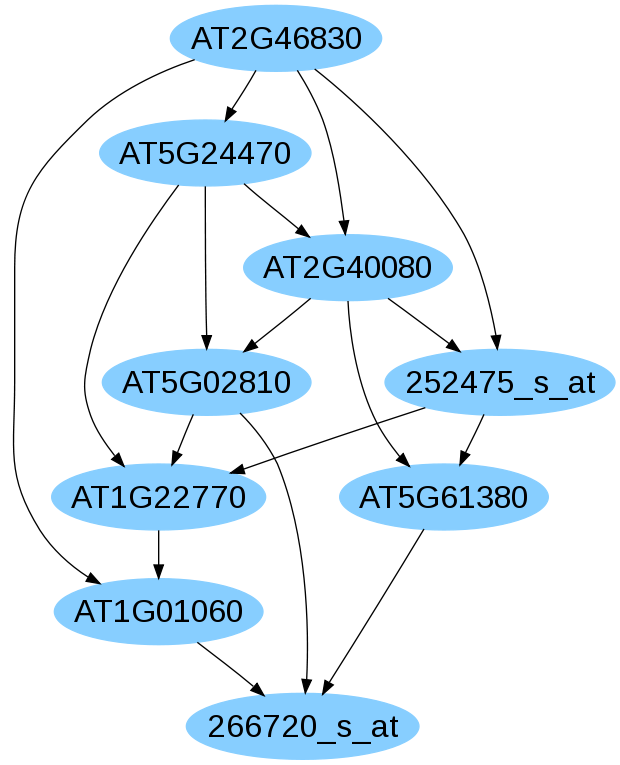

Supplement: Additional file 1 — Mini-website showing all learned network graphs for examples presented. Mini-website showing all learned network graphs at each iteration for the examples presented in the main body of the paper, and a table of the genes involved. [file 1752-0509-3-85-S1.zip › S/net1/grn_9.png]

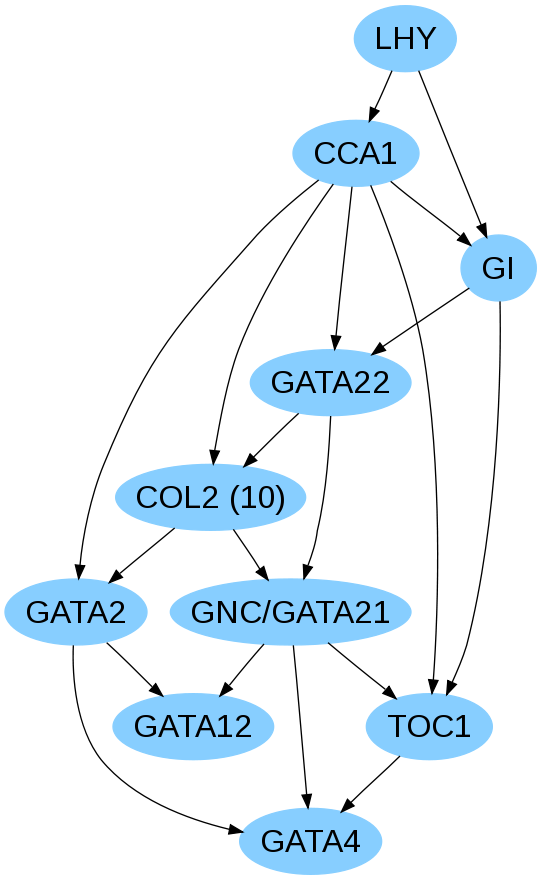

Supplement: Additional file 1 — Mini-website showing all learned network graphs for examples presented. Mini-website showing all learned network graphs at each iteration for the examples presented in the main body of the paper, and a table of the genes involved. [file 1752-0509-3-85-S1.zip › S/net2/grn_10.png]

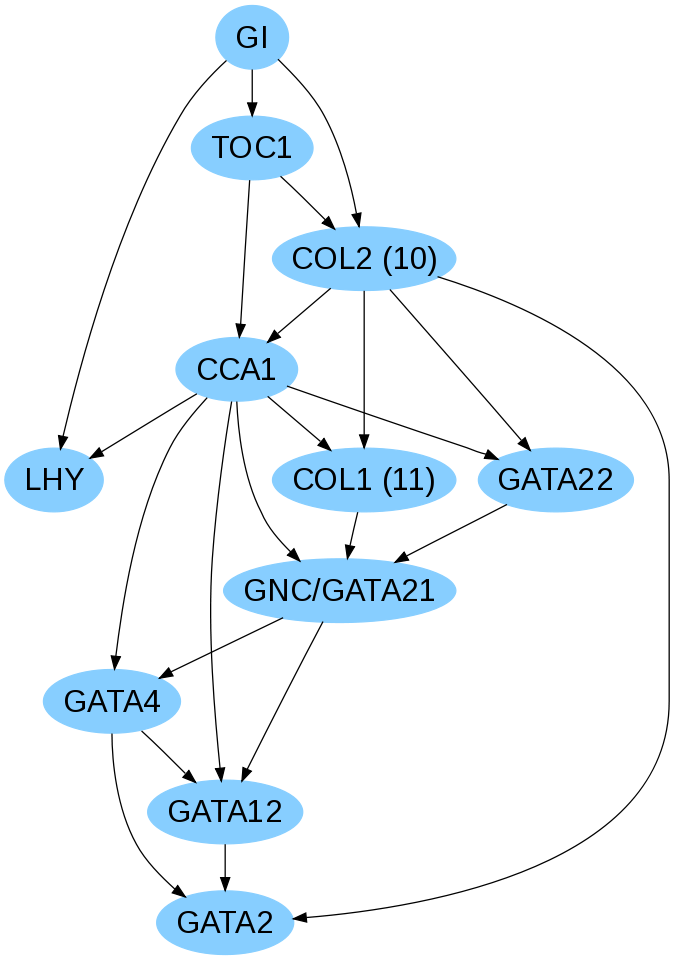

Supplement: Additional file 1 — Mini-website showing all learned network graphs for examples presented. Mini-website showing all learned network graphs at each iteration for the examples presented in the main body of the paper, and a table of the genes involved. [file 1752-0509-3-85-S1.zip › S/net2/grn_11.png]

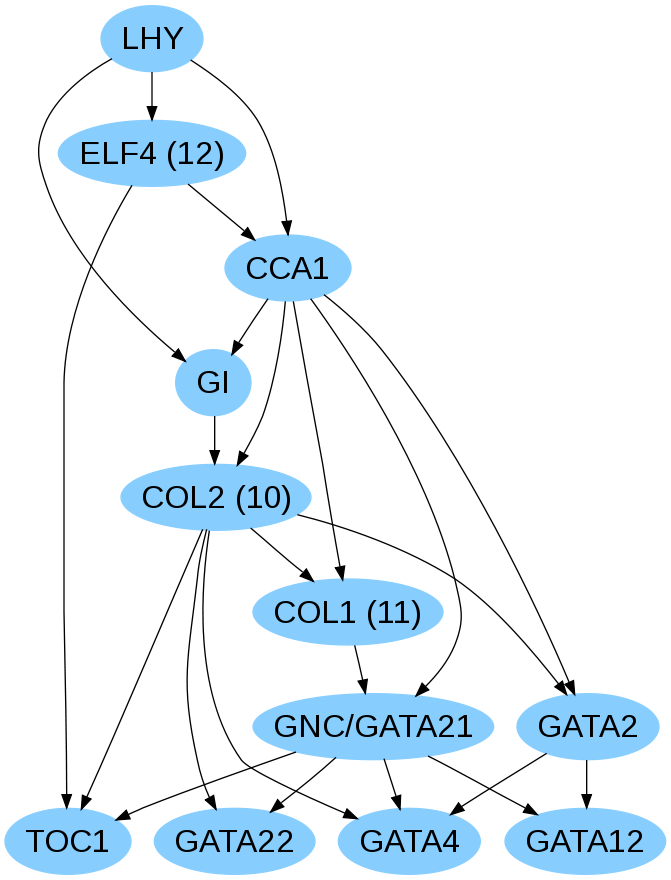

Supplement: Additional file 1 — Mini-website showing all learned network graphs for examples presented. Mini-website showing all learned network graphs at each iteration for the examples presented in the main body of the paper, and a table of the genes involved. [file 1752-0509-3-85-S1.zip › S/net2/grn_12.png]

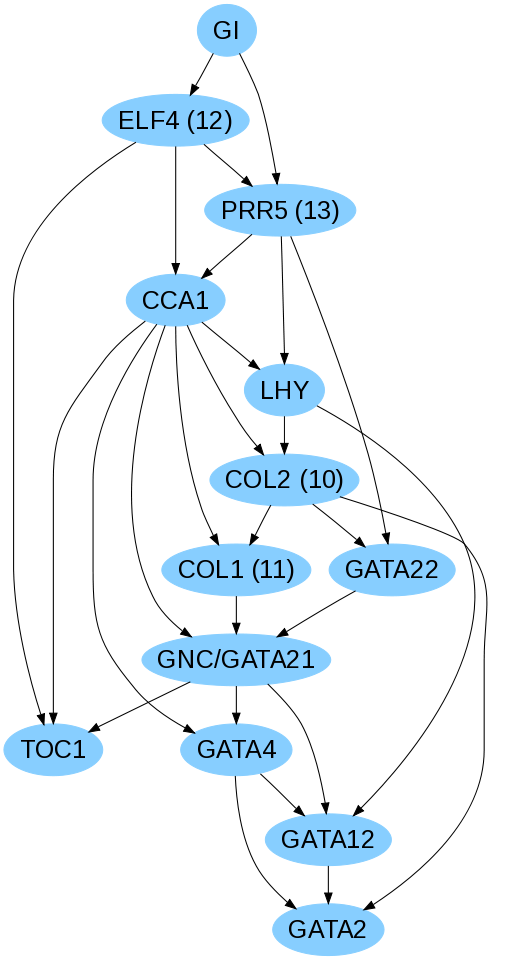

Supplement: Additional file 1 — Mini-website showing all learned network graphs for examples presented. Mini-website showing all learned network graphs at each iteration for the examples presented in the main body of the paper, and a table of the genes involved. [file 1752-0509-3-85-S1.zip › S/net2/grn_13.png]

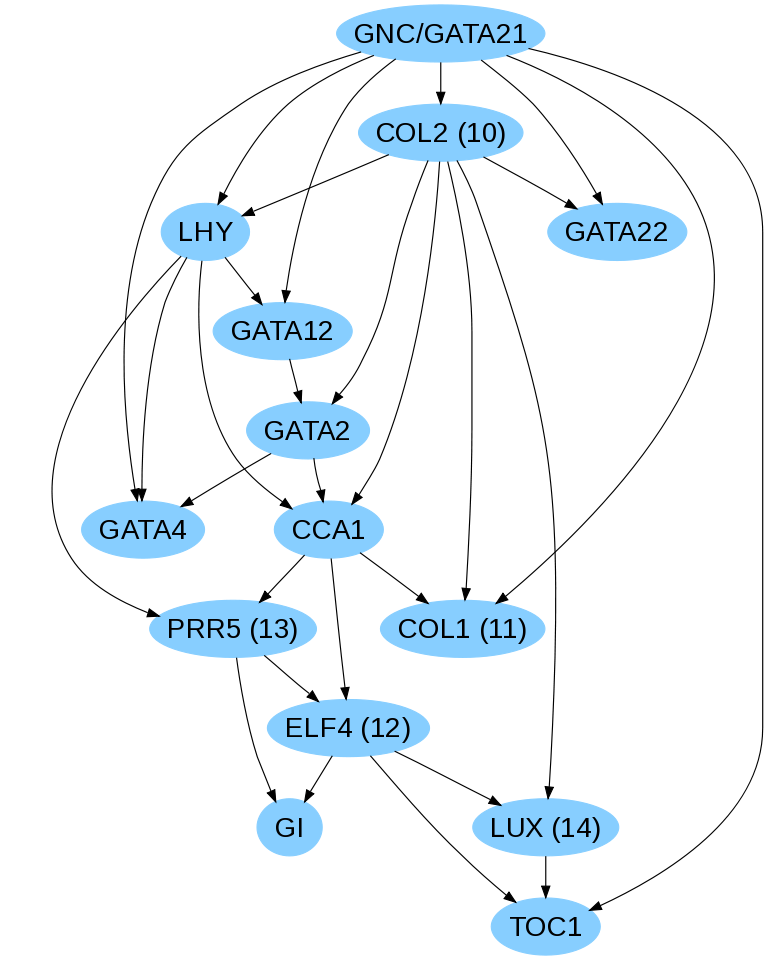

Supplement: Additional file 1 — Mini-website showing all learned network graphs for examples presented. Mini-website showing all learned network graphs at each iteration for the examples presented in the main body of the paper, and a table of the genes involved. [file 1752-0509-3-85-S1.zip › S/net2/grn_14.png]

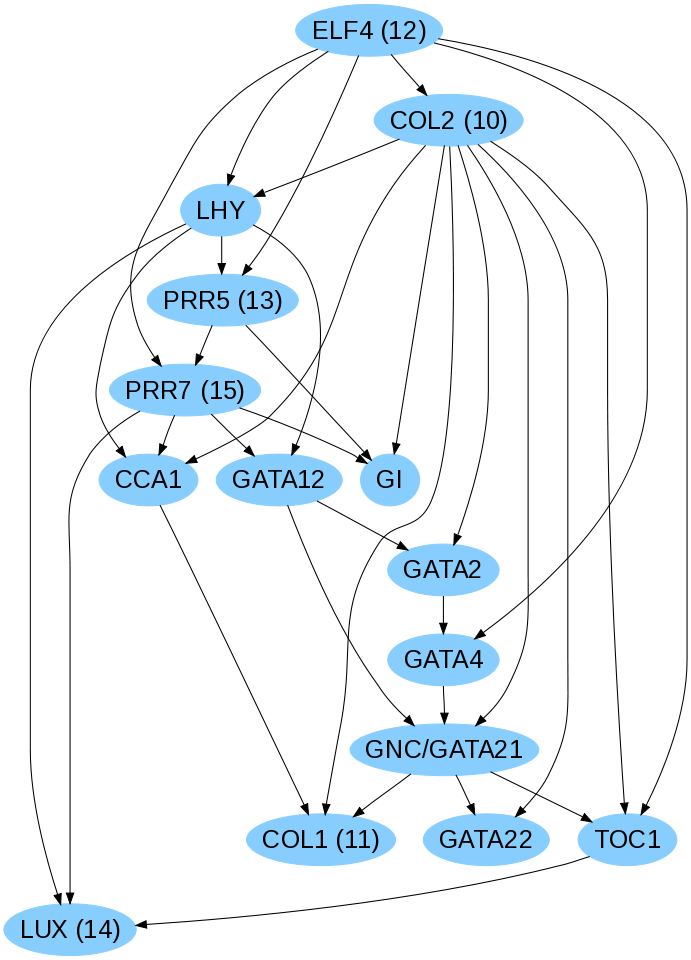

Supplement: Additional file 1 — Mini-website showing all learned network graphs for examples presented. Mini-website showing all learned network graphs at each iteration for the examples presented in the main body of the paper, and a table of the genes involved. [file 1752-0509-3-85-S1.zip › S/net2/grn_15.png]

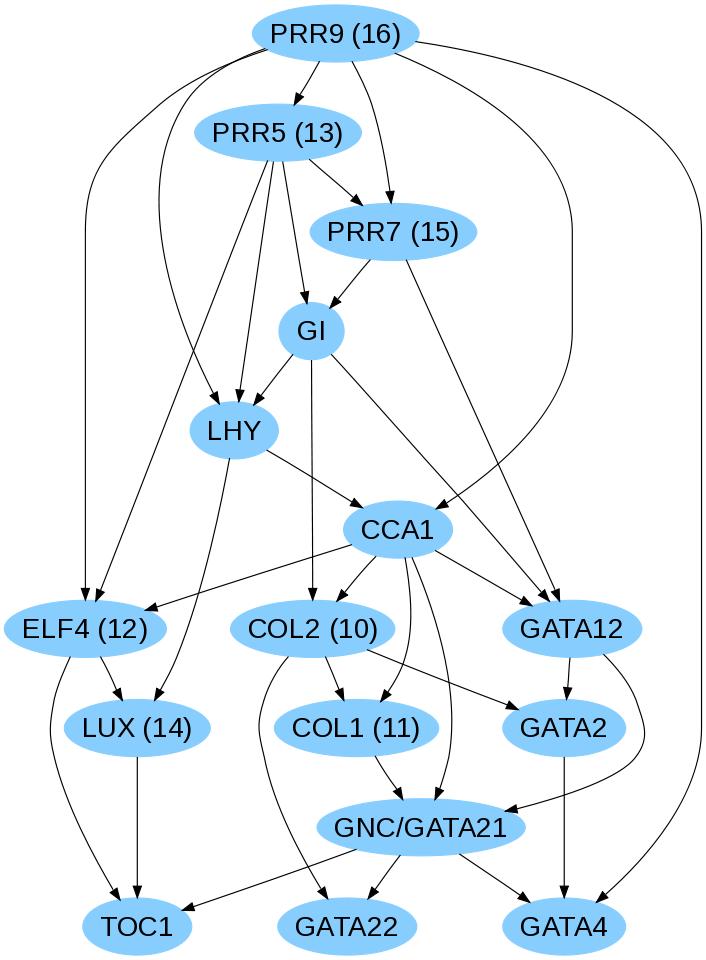

Supplement: Additional file 1 — Mini-website showing all learned network graphs for examples presented. Mini-website showing all learned network graphs at each iteration for the examples presented in the main body of the paper, and a table of the genes involved. [file 1752-0509-3-85-S1.zip › S/net2/grn_16.png]

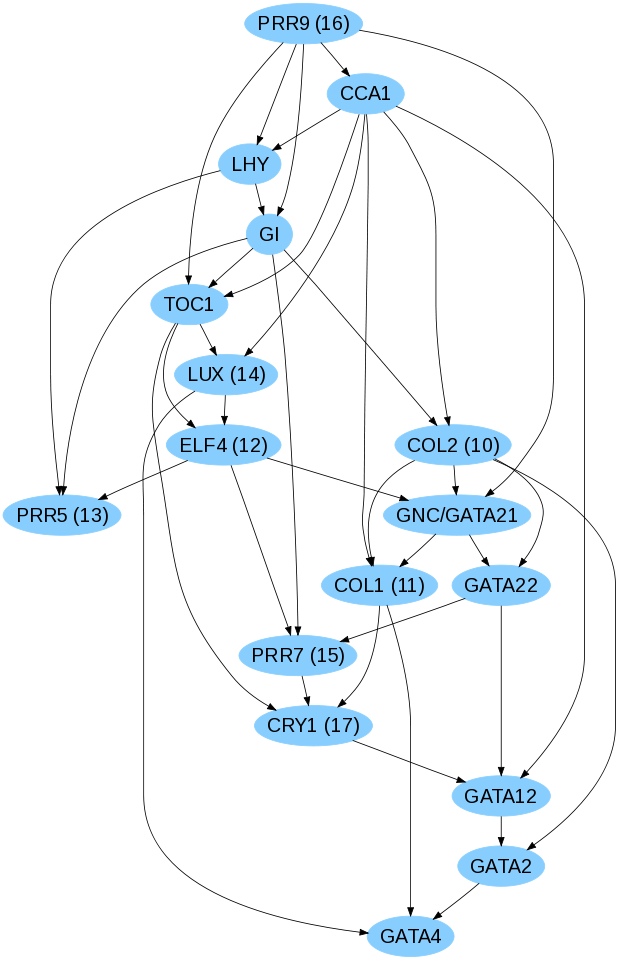

Supplement: Additional file 1 — Mini-website showing all learned network graphs for examples presented. Mini-website showing all learned network graphs at each iteration for the examples presented in the main body of the paper, and a table of the genes involved. [file 1752-0509-3-85-S1.zip › S/net2/grn_17.png]

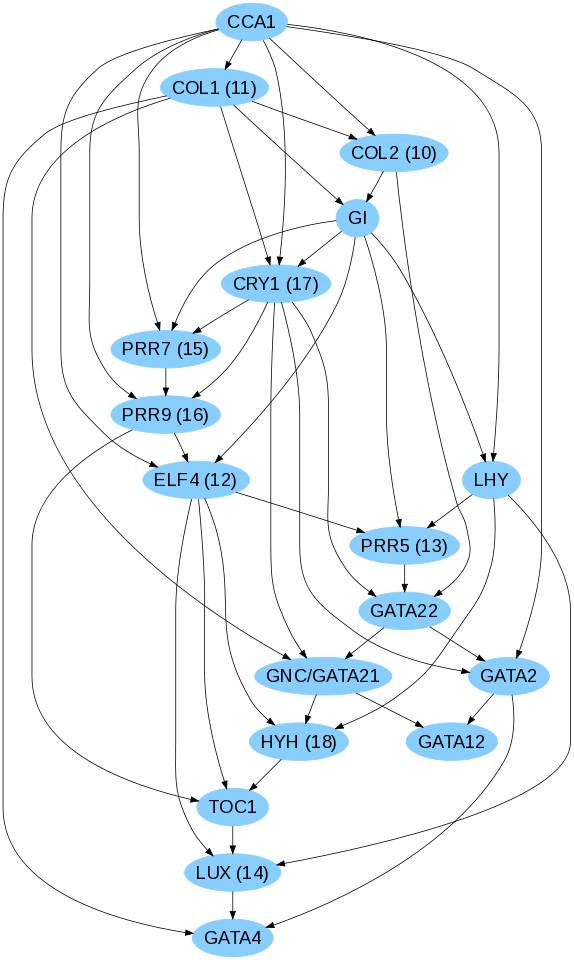

Supplement: Additional file 1 — Mini-website showing all learned network graphs for examples presented. Mini-website showing all learned network graphs at each iteration for the examples presented in the main body of the paper, and a table of the genes involved. [file 1752-0509-3-85-S1.zip › S/net2/grn_18.png]

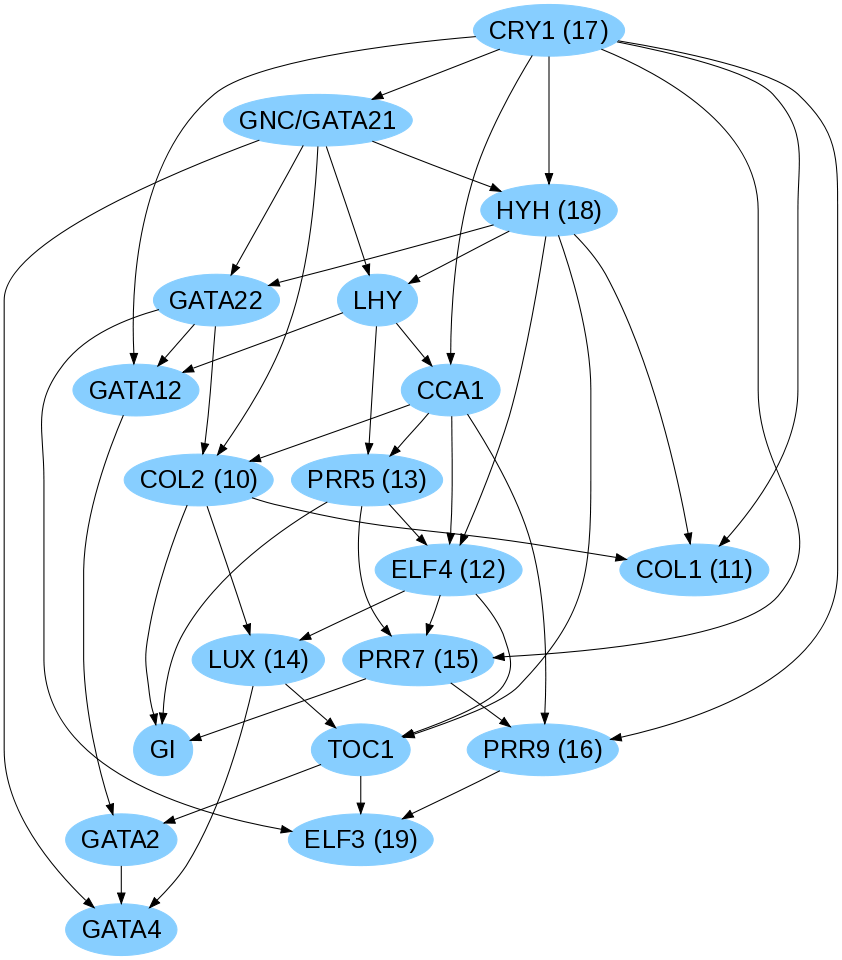

Supplement: Additional file 1 — Mini-website showing all learned network graphs for examples presented. Mini-website showing all learned network graphs at each iteration for the examples presented in the main body of the paper, and a table of the genes involved. [file 1752-0509-3-85-S1.zip › S/net2/grn_19.png]

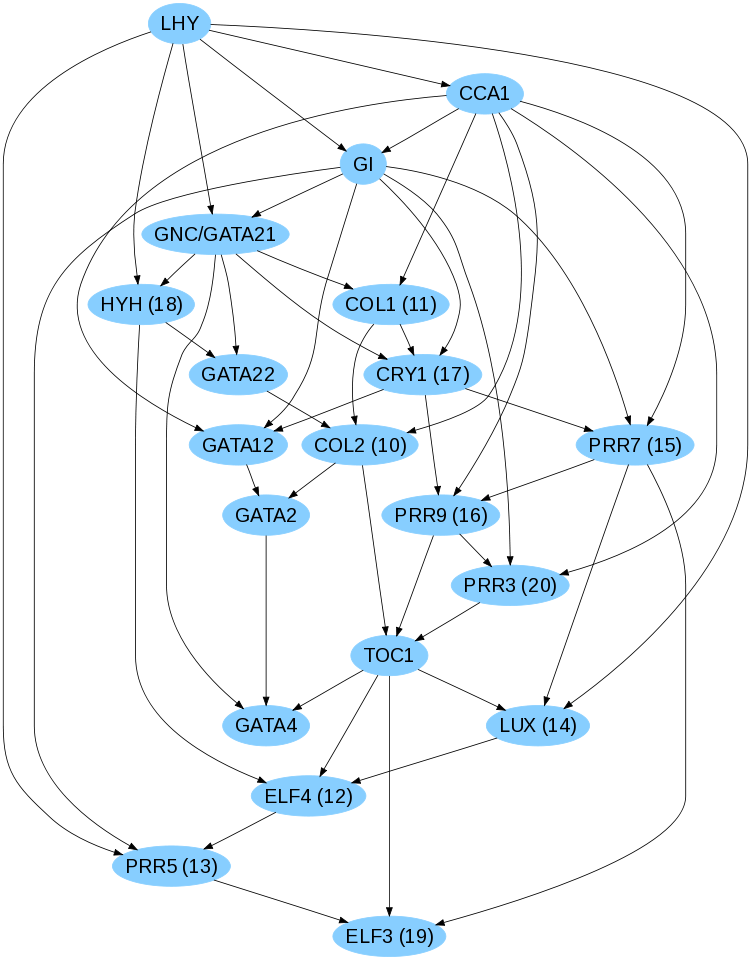

Supplement: Additional file 1 — Mini-website showing all learned network graphs for examples presented. Mini-website showing all learned network graphs at each iteration for the examples presented in the main body of the paper, and a table of the genes involved. [file 1752-0509-3-85-S1.zip › S/net2/grn_20.png]

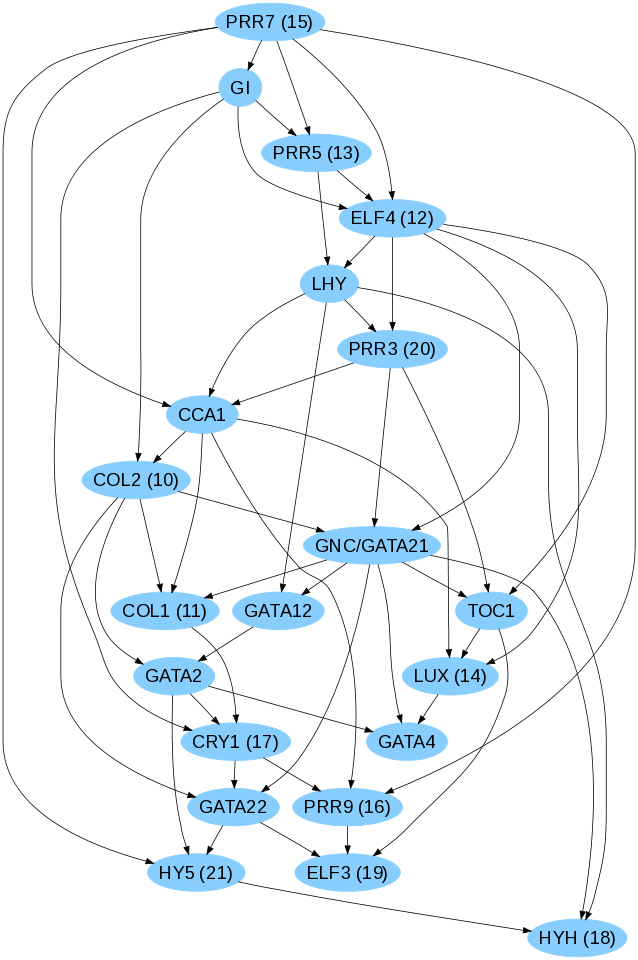

Supplement: Additional file 1 — Mini-website showing all learned network graphs for examples presented. Mini-website showing all learned network graphs at each iteration for the examples presented in the main body of the paper, and a table of the genes involved. [file 1752-0509-3-85-S1.zip › S/net2/grn_21.png]

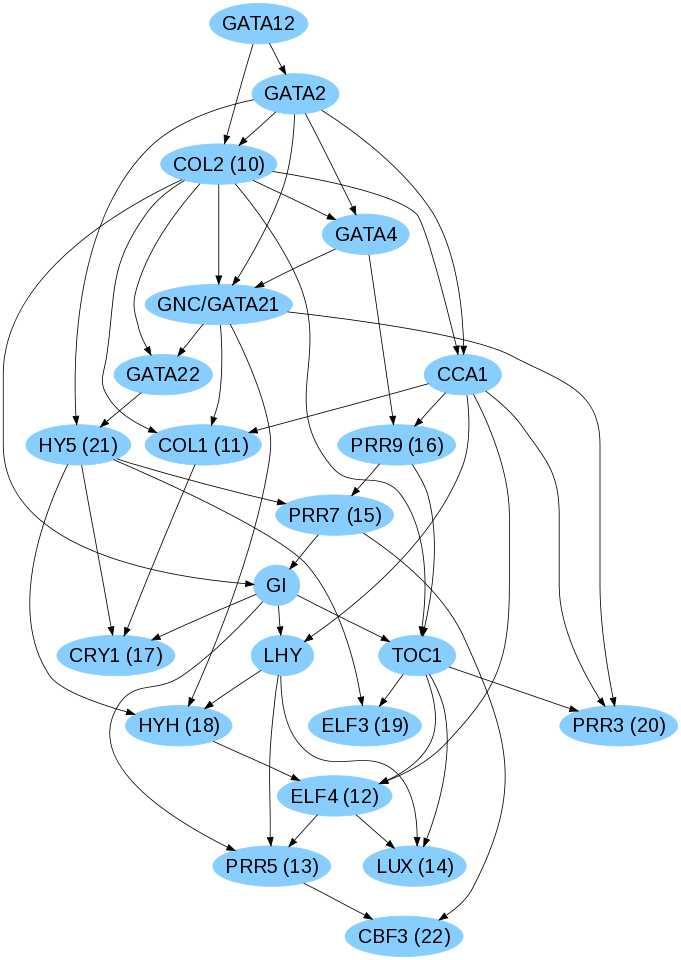

Supplement: Additional file 1 — Mini-website showing all learned network graphs for examples presented. Mini-website showing all learned network graphs at each iteration for the examples presented in the main body of the paper, and a table of the genes involved. [file 1752-0509-3-85-S1.zip › S/net2/grn_22.png]

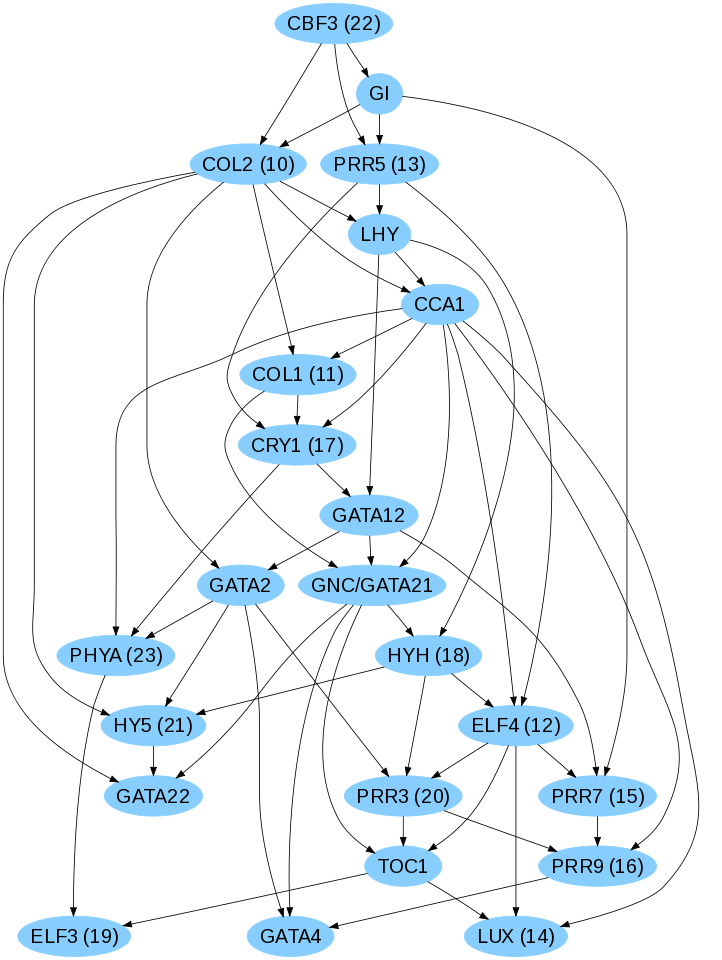

Supplement: Additional file 1 — Mini-website showing all learned network graphs for examples presented. Mini-website showing all learned network graphs at each iteration for the examples presented in the main body of the paper, and a table of the genes involved. [file 1752-0509-3-85-S1.zip › S/net2/grn_23.png]

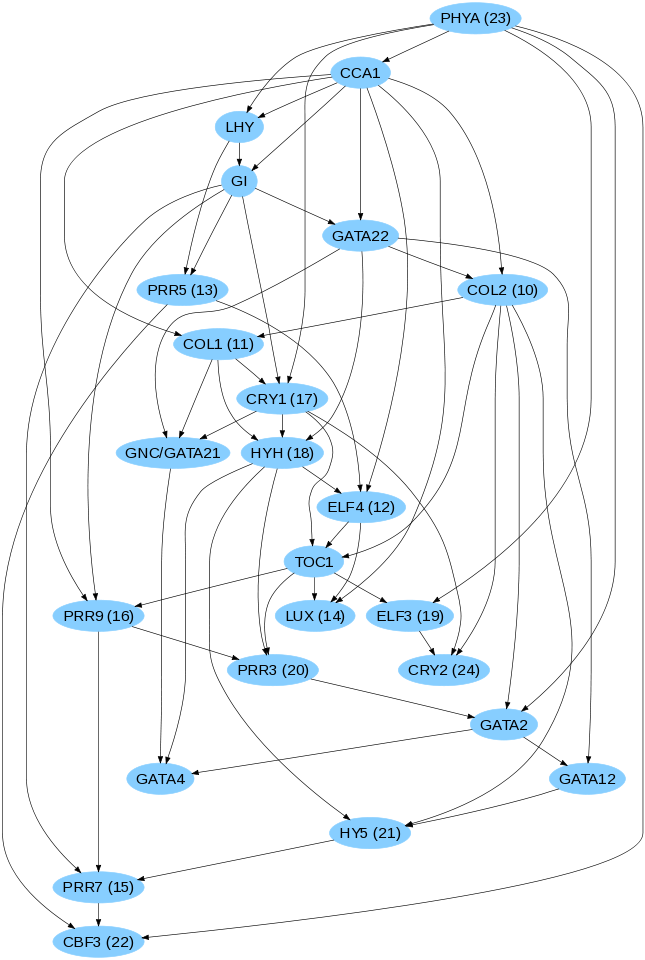

Supplement: Additional file 1 — Mini-website showing all learned network graphs for examples presented. Mini-website showing all learned network graphs at each iteration for the examples presented in the main body of the paper, and a table of the genes involved. [file 1752-0509-3-85-S1.zip › S/net2/grn_24.png]

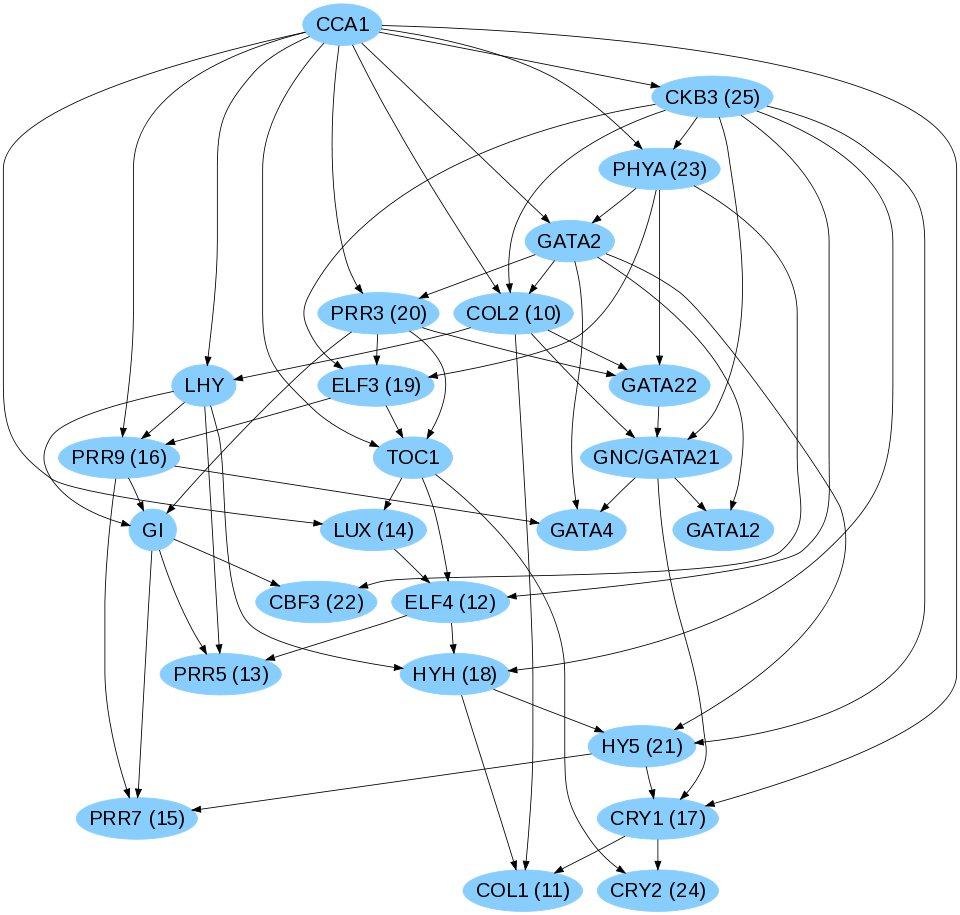

Supplement: Additional file 1 — Mini-website showing all learned network graphs for examples presented. Mini-website showing all learned network graphs at each iteration for the examples presented in the main body of the paper, and a table of the genes involved. [file 1752-0509-3-85-S1.zip › S/net2/grn_25.png]

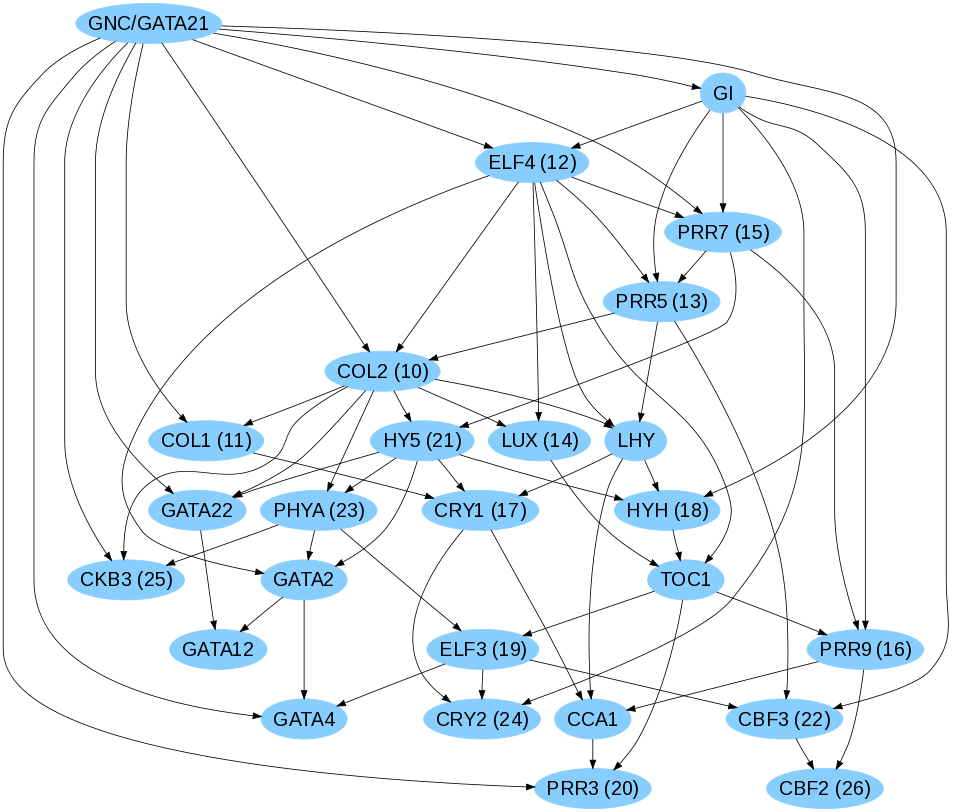

Supplement: Additional file 1 — Mini-website showing all learned network graphs for examples presented. Mini-website showing all learned network graphs at each iteration for the examples presented in the main body of the paper, and a table of the genes involved. [file 1752-0509-3-85-S1.zip › S/net2/grn_26.png]

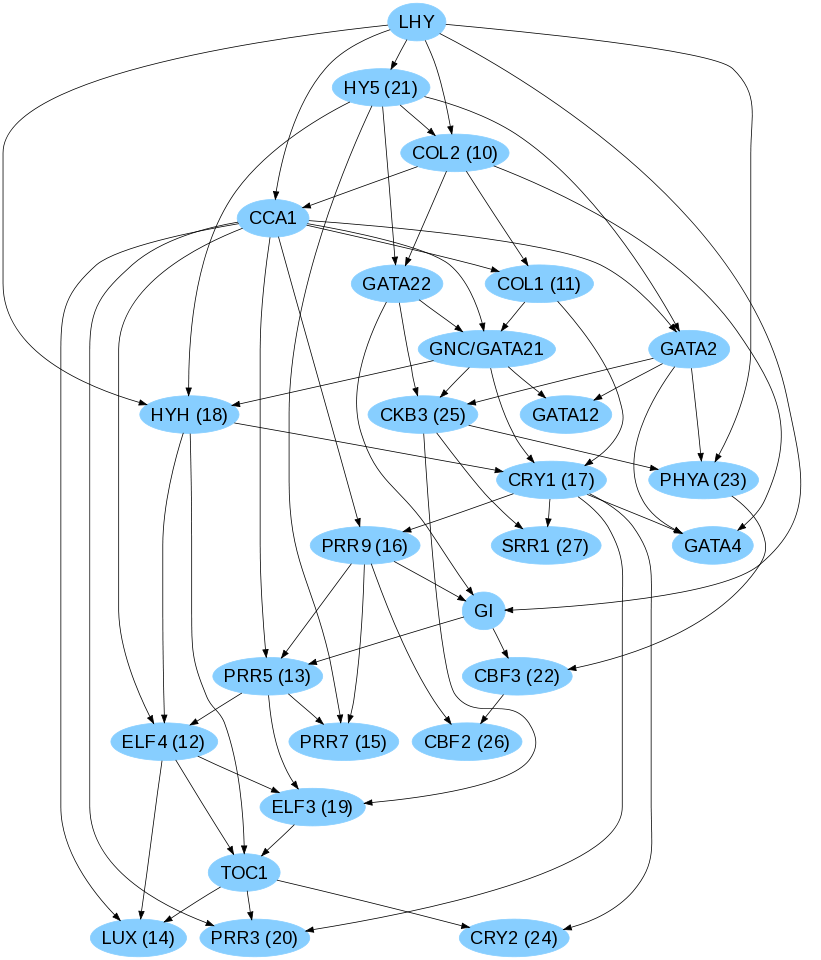

Supplement: Additional file 1 — Mini-website showing all learned network graphs for examples presented. Mini-website showing all learned network graphs at each iteration for the examples presented in the main body of the paper, and a table of the genes involved. [file 1752-0509-3-85-S1.zip › S/net2/grn_27.png]

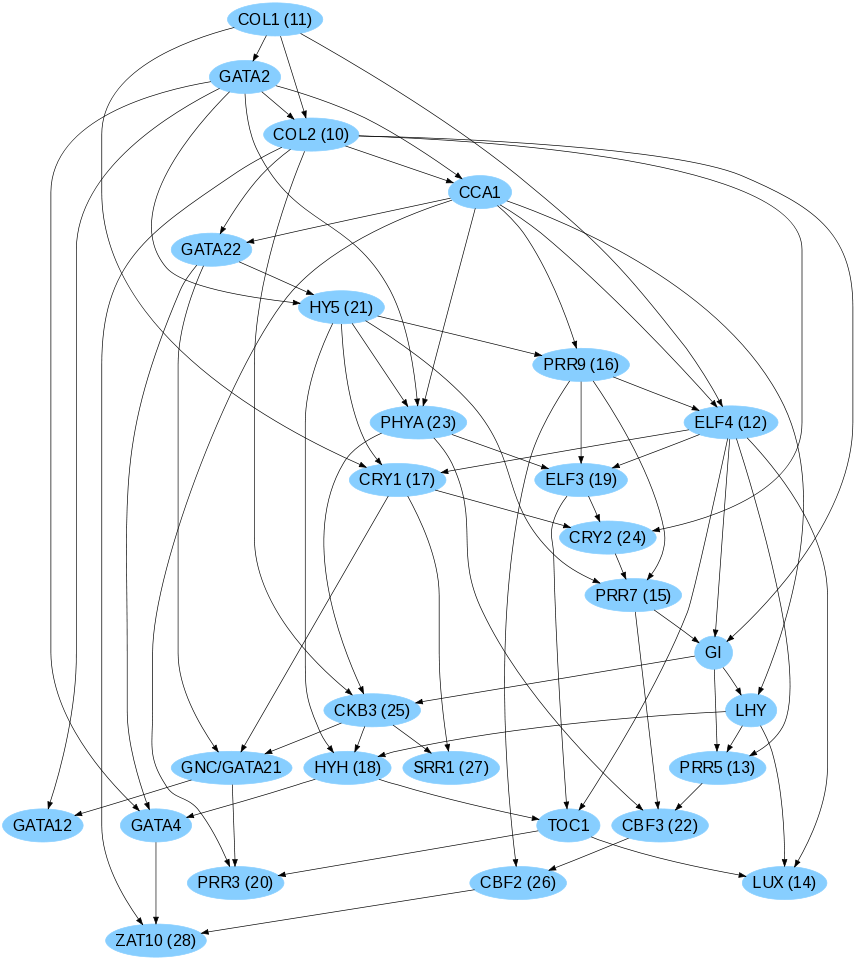

Supplement: Additional file 1 — Mini-website showing all learned network graphs for examples presented. Mini-website showing all learned network graphs at each iteration for the examples presented in the main body of the paper, and a table of the genes involved. [file 1752-0509-3-85-S1.zip › S/net2/grn_28.png]

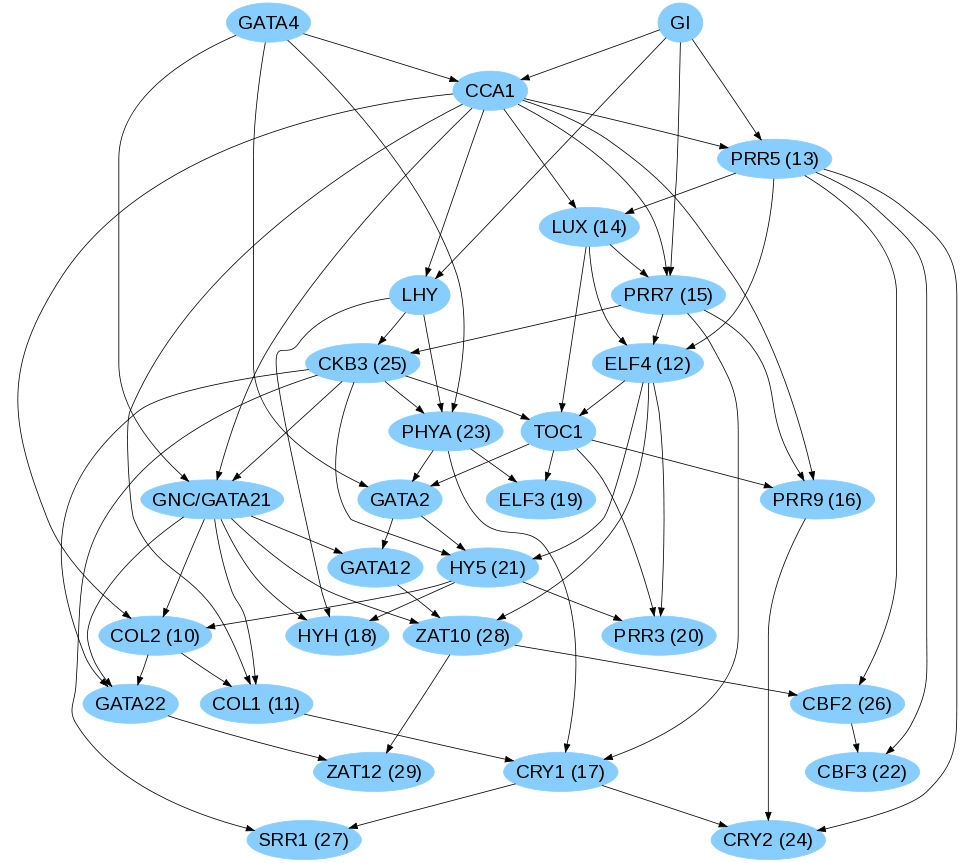

Supplement: Additional file 1 — Mini-website showing all learned network graphs for examples presented. Mini-website showing all learned network graphs at each iteration for the examples presented in the main body of the paper, and a table of the genes involved. [file 1752-0509-3-85-S1.zip › S/net2/grn_29.png]

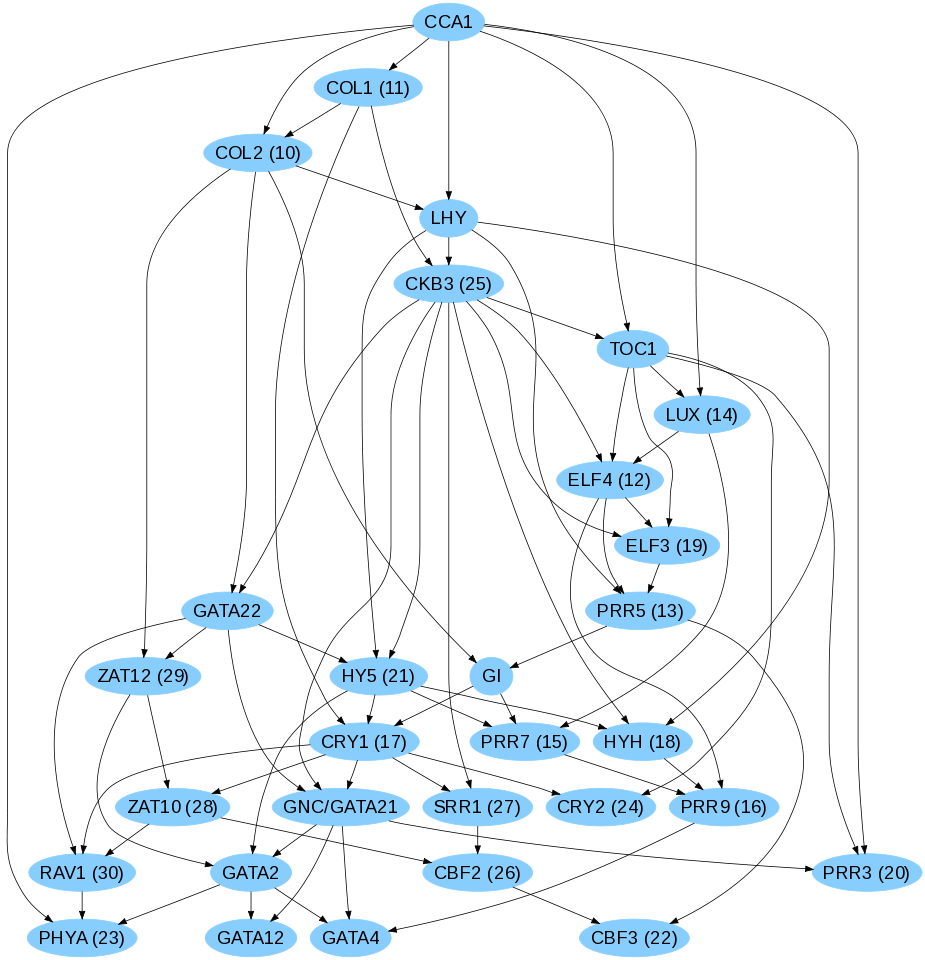

Supplement: Additional file 1 — Mini-website showing all learned network graphs for examples presented. Mini-website showing all learned network graphs at each iteration for the examples presented in the main body of the paper, and a table of the genes involved. [file 1752-0509-3-85-S1.zip › S/net2/grn_30.png]

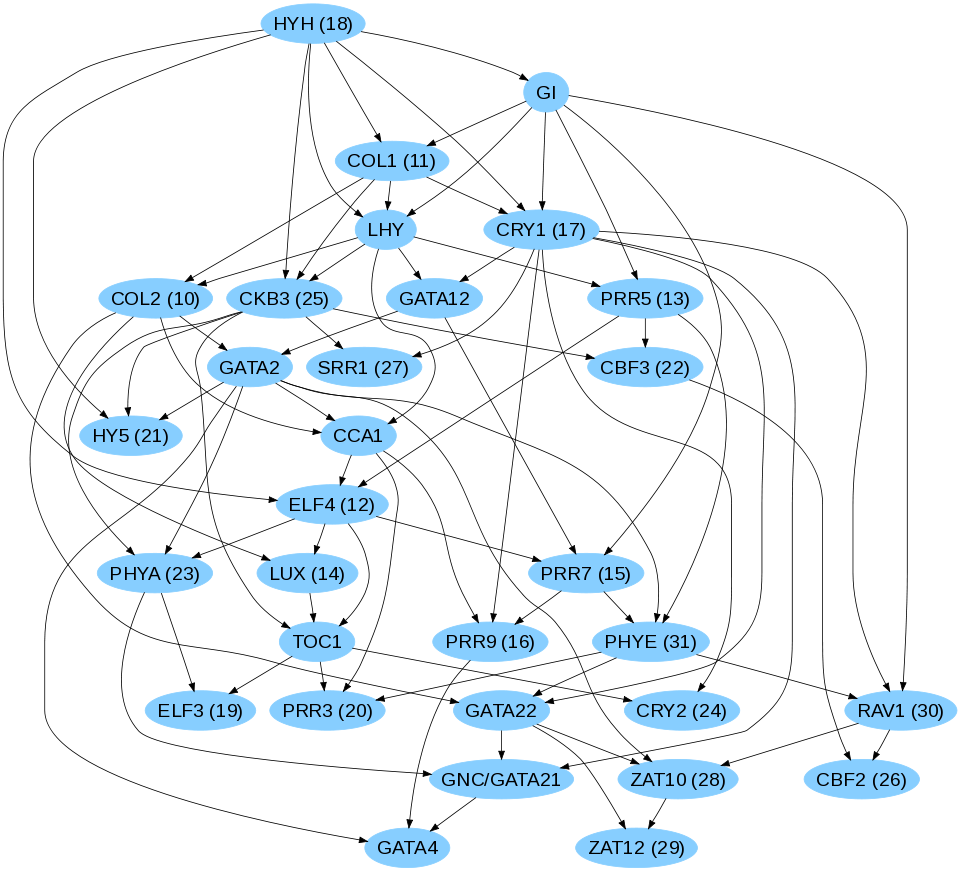

Supplement: Additional file 1 — Mini-website showing all learned network graphs for examples presented. Mini-website showing all learned network graphs at each iteration for the examples presented in the main body of the paper, and a table of the genes involved. [file 1752-0509-3-85-S1.zip › S/net2/grn_31.png]

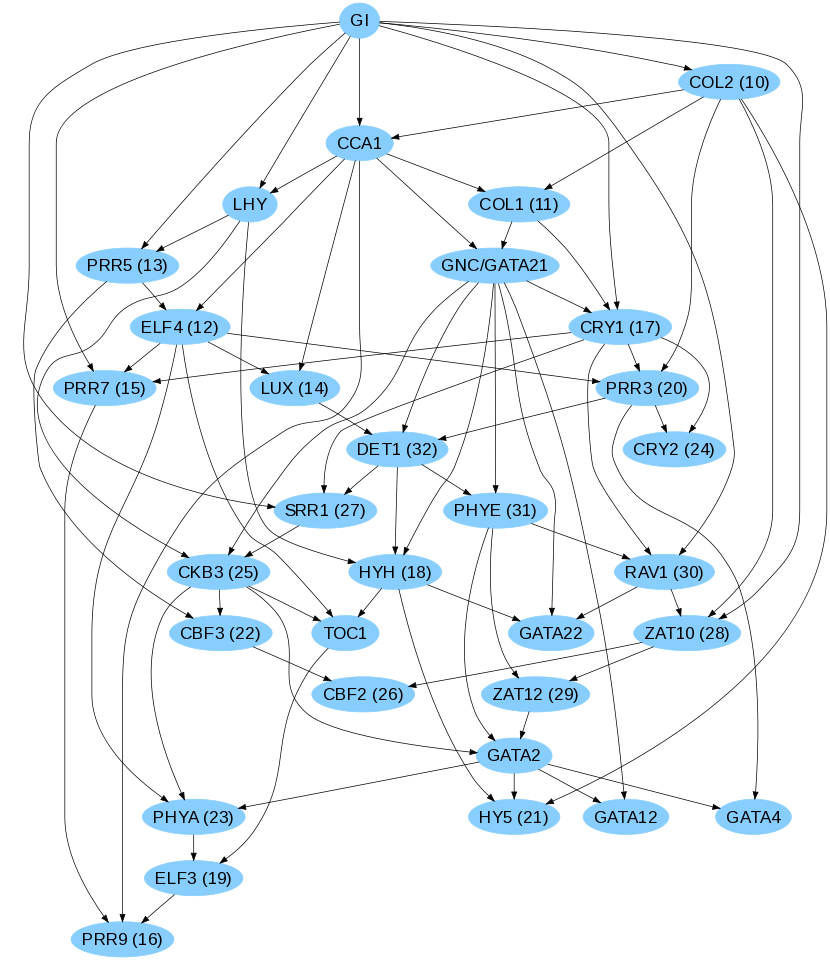

Supplement: Additional file 1 — Mini-website showing all learned network graphs for examples presented. Mini-website showing all learned network graphs at each iteration for the examples presented in the main body of the paper, and a table of the genes involved. [file 1752-0509-3-85-S1.zip › S/net2/grn_32.png]

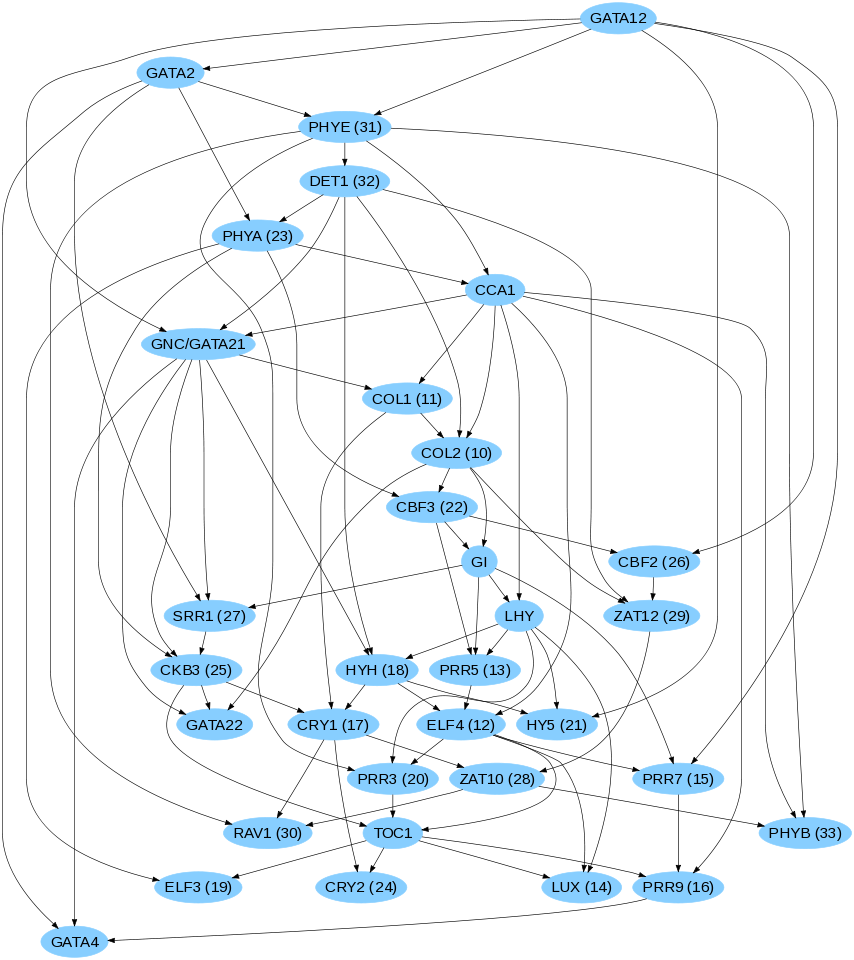

Supplement: Additional file 1 — Mini-website showing all learned network graphs for examples presented. Mini-website showing all learned network graphs at each iteration for the examples presented in the main body of the paper, and a table of the genes involved. [file 1752-0509-3-85-S1.zip › S/net2/grn_33.png]

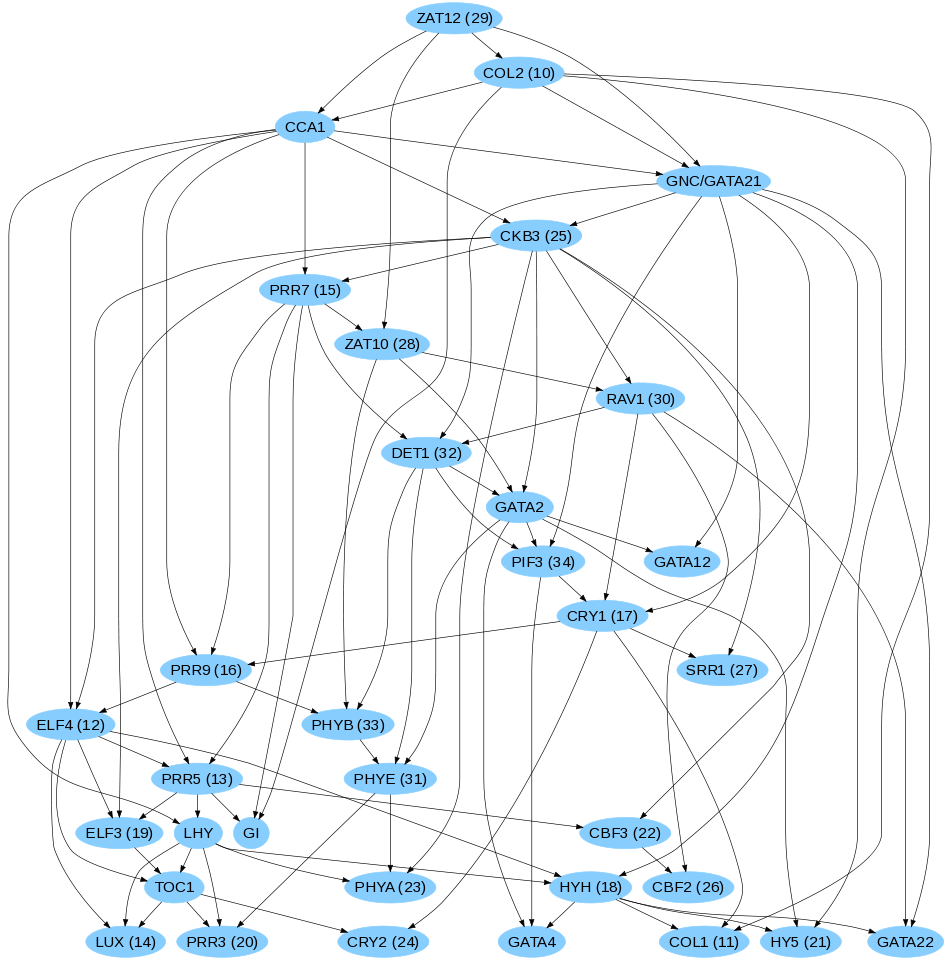

Supplement: Additional file 1 — Mini-website showing all learned network graphs for examples presented. Mini-website showing all learned network graphs at each iteration for the examples presented in the main body of the paper, and a table of the genes involved. [file 1752-0509-3-85-S1.zip › S/net2/grn_34.png]

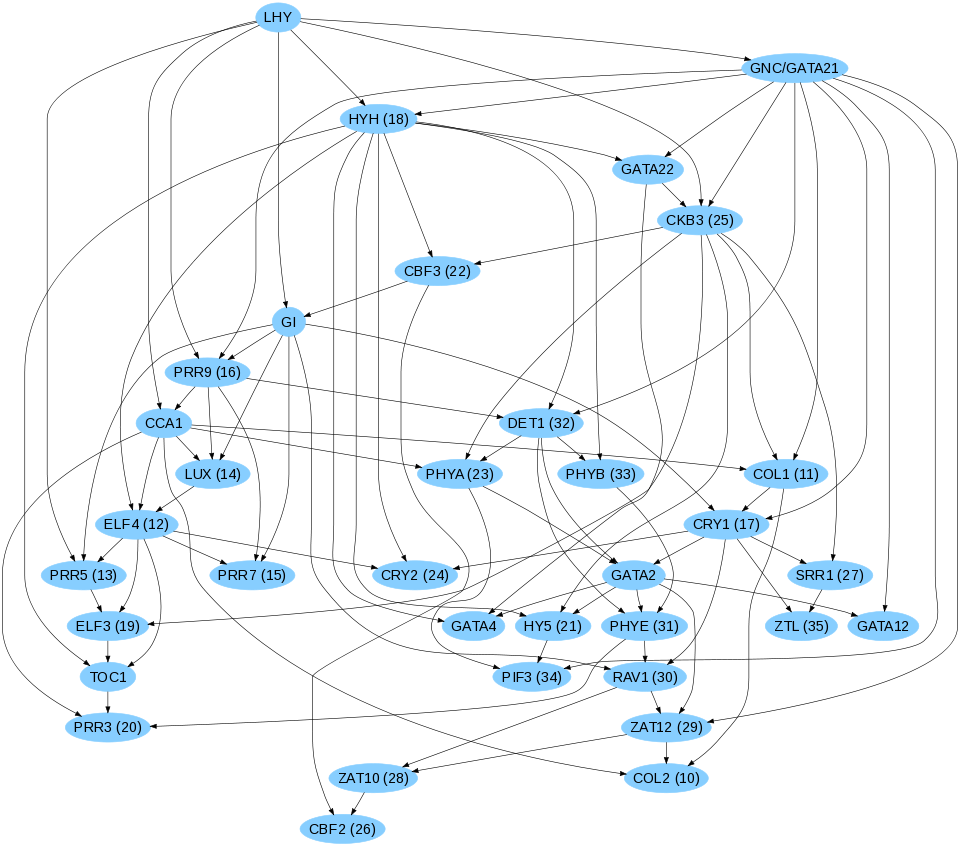

Supplement: Additional file 1 — Mini-website showing all learned network graphs for examples presented. Mini-website showing all learned network graphs at each iteration for the examples presented in the main body of the paper, and a table of the genes involved. [file 1752-0509-3-85-S1.zip › S/net2/grn_35.png]

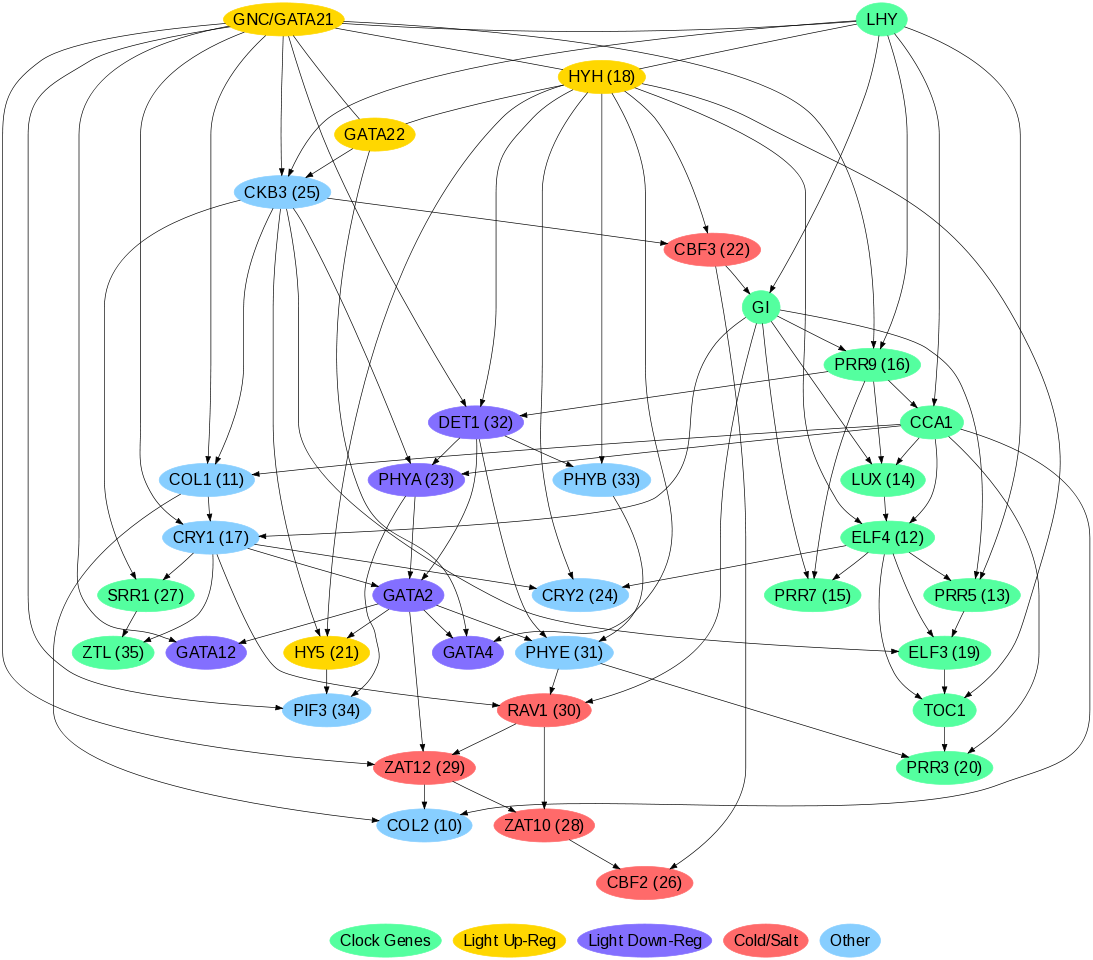

Supplement: Additional file 1 — Mini-website showing all learned network graphs for examples presented. Mini-website showing all learned network graphs at each iteration for the examples presented in the main body of the paper, and a table of the genes involved. [file 1752-0509-3-85-S1.zip › S/net2/grn35.png]

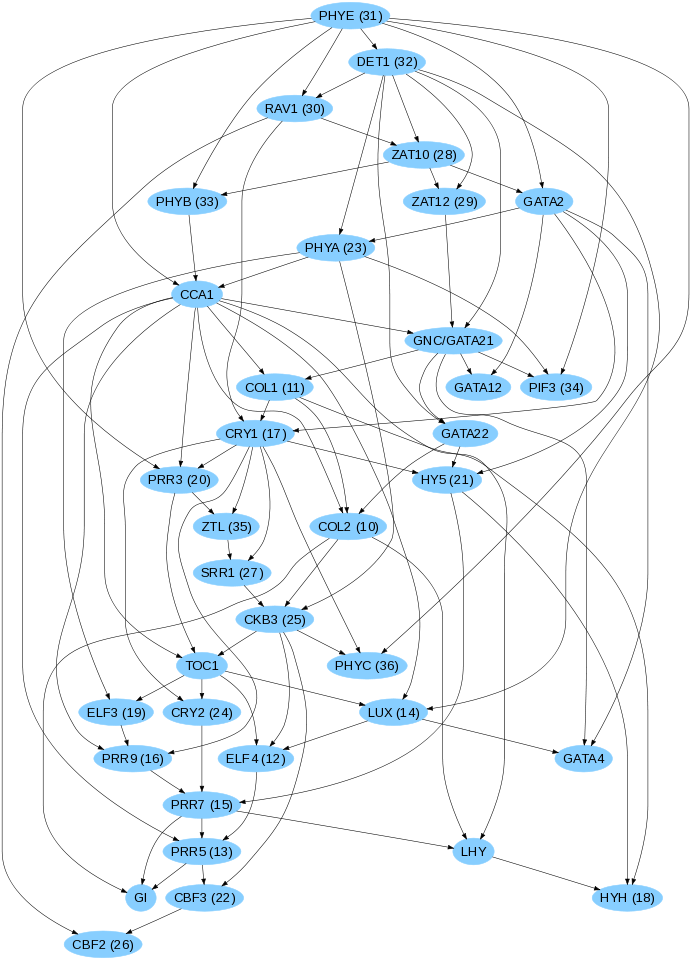

Supplement: Additional file 1 — Mini-website showing all learned network graphs for examples presented. Mini-website showing all learned network graphs at each iteration for the examples presented in the main body of the paper, and a table of the genes involved. [file 1752-0509-3-85-S1.zip › S/net2/grn_36.png]

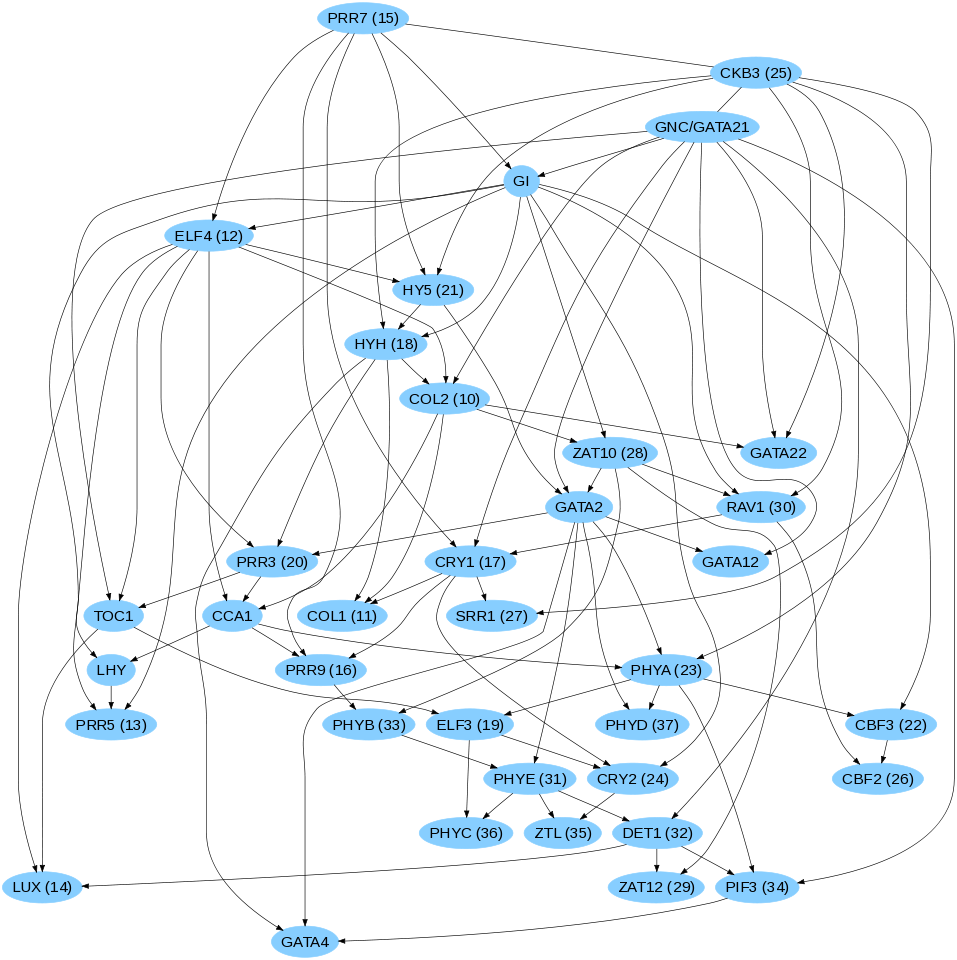

Supplement: Additional file 1 — Mini-website showing all learned network graphs for examples presented. Mini-website showing all learned network graphs at each iteration for the examples presented in the main body of the paper, and a table of the genes involved. [file 1752-0509-3-85-S1.zip › S/net2/grn_37.png]

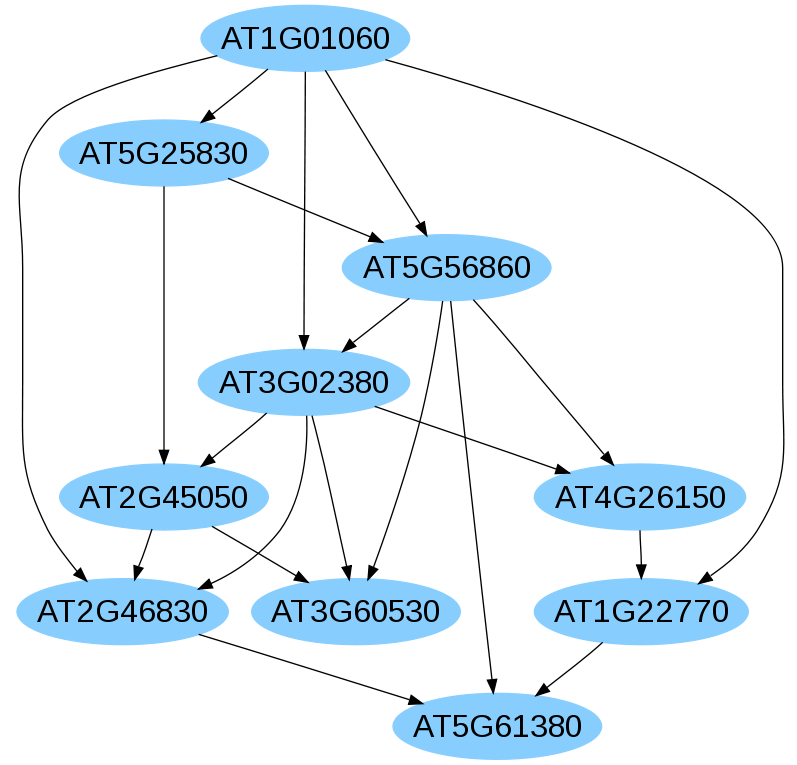

Supplement: Additional file 1 — Mini-website showing all learned network graphs for examples presented. Mini-website showing all learned network graphs at each iteration for the examples presented in the main body of the paper, and a table of the genes involved. [file 1752-0509-3-85-S1.zip › S/net3/grn_10.png]

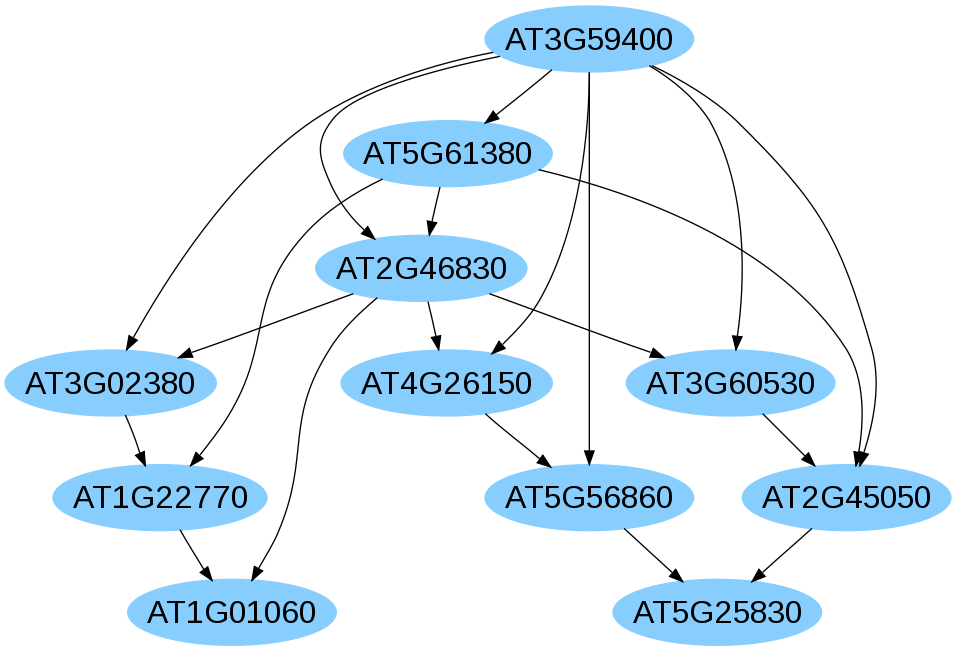

Supplement: Additional file 1 — Mini-website showing all learned network graphs for examples presented. Mini-website showing all learned network graphs at each iteration for the examples presented in the main body of the paper, and a table of the genes involved. [file 1752-0509-3-85-S1.zip › S/net3/grn_11.png]

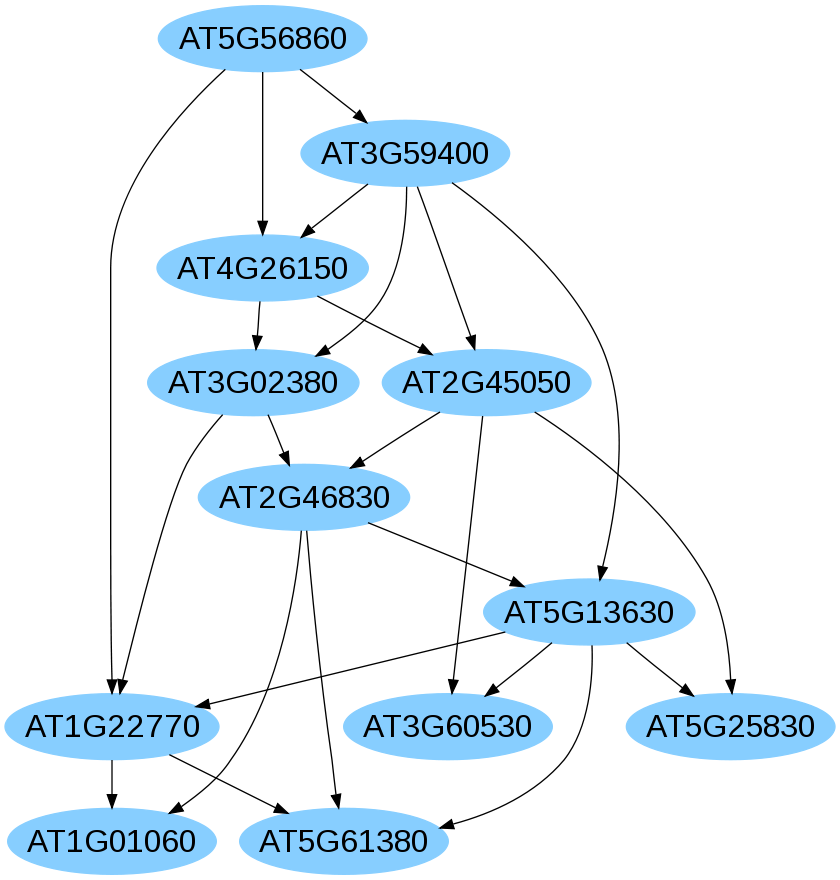

Supplement: Additional file 1 — Mini-website showing all learned network graphs for examples presented. Mini-website showing all learned network graphs at each iteration for the examples presented in the main body of the paper, and a table of the genes involved. [file 1752-0509-3-85-S1.zip › S/net3/grn_12.png]

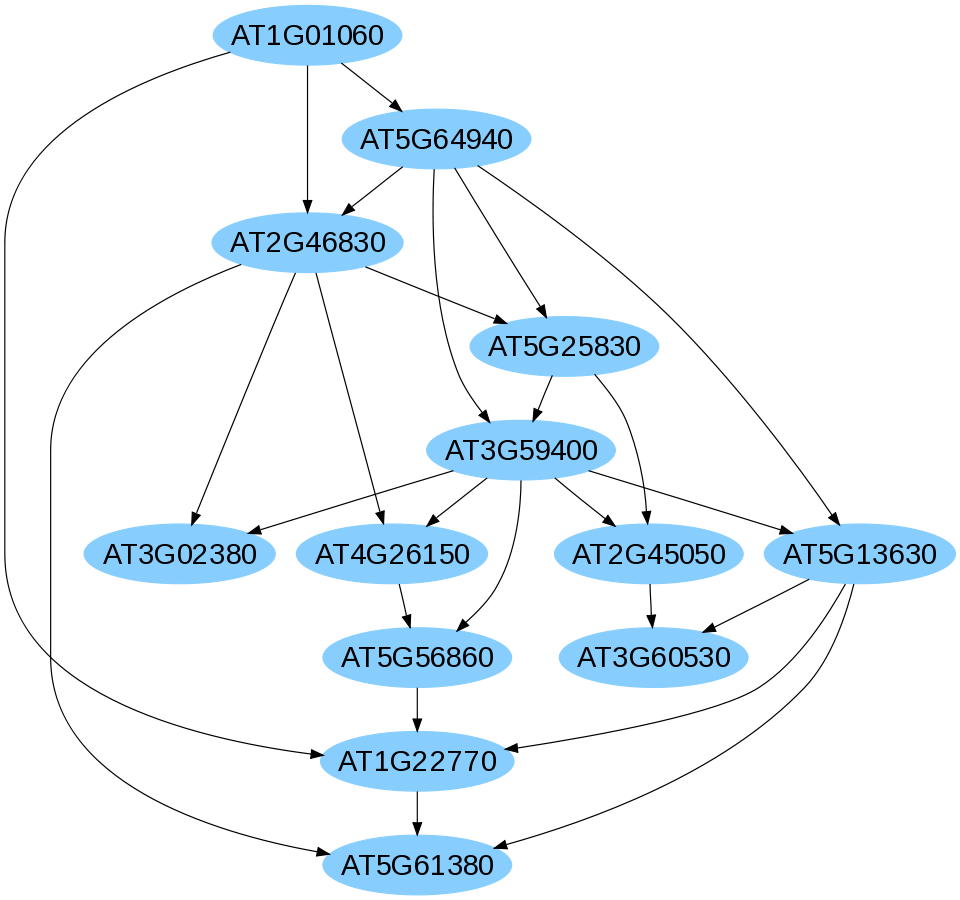

Supplement: Additional file 1 — Mini-website showing all learned network graphs for examples presented. Mini-website showing all learned network graphs at each iteration for the examples presented in the main body of the paper, and a table of the genes involved. [file 1752-0509-3-85-S1.zip › S/net3/grn_13.png]

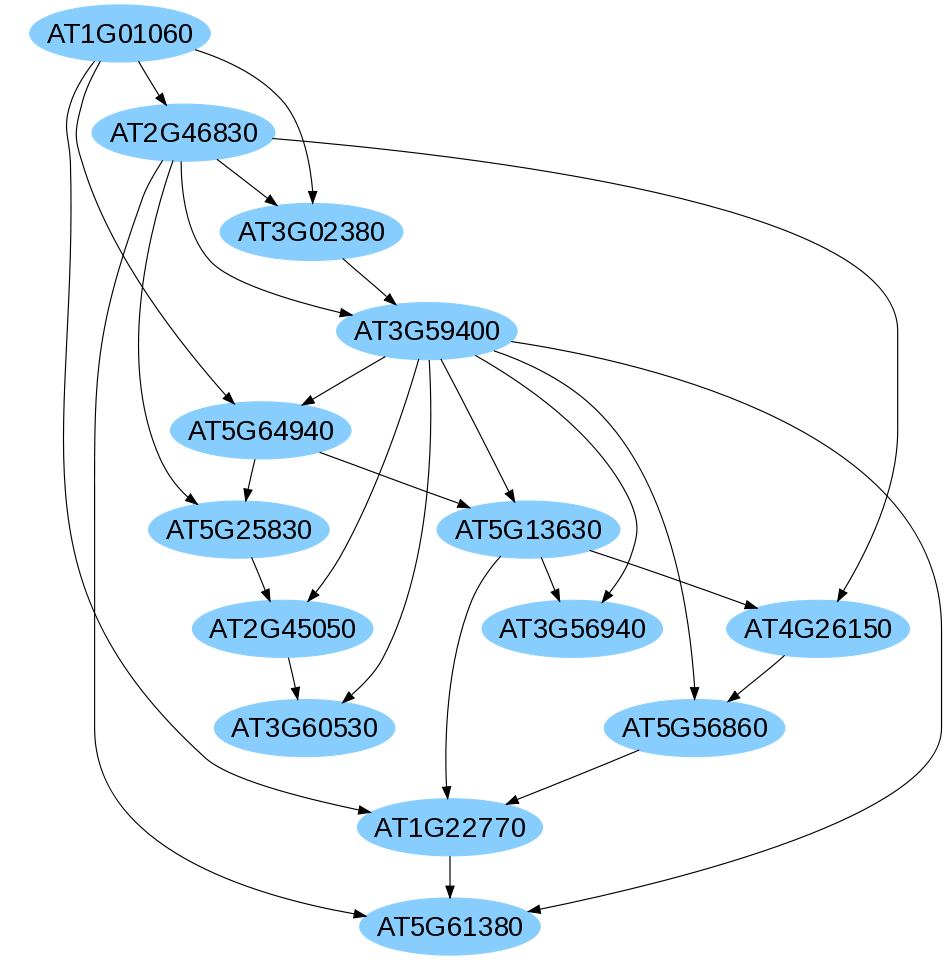

Supplement: Additional file 1 — Mini-website showing all learned network graphs for examples presented. Mini-website showing all learned network graphs at each iteration for the examples presented in the main body of the paper, and a table of the genes involved. [file 1752-0509-3-85-S1.zip › S/net3/grn_14.png]

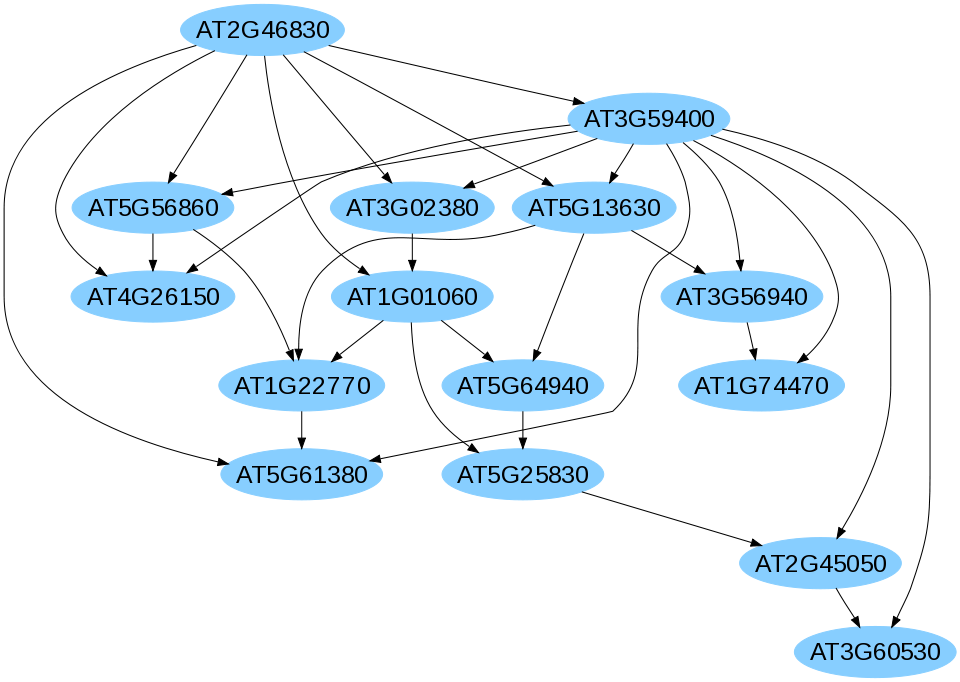

Supplement: Additional file 1 — Mini-website showing all learned network graphs for examples presented. Mini-website showing all learned network graphs at each iteration for the examples presented in the main body of the paper, and a table of the genes involved. [file 1752-0509-3-85-S1.zip › S/net3/grn_15.png]

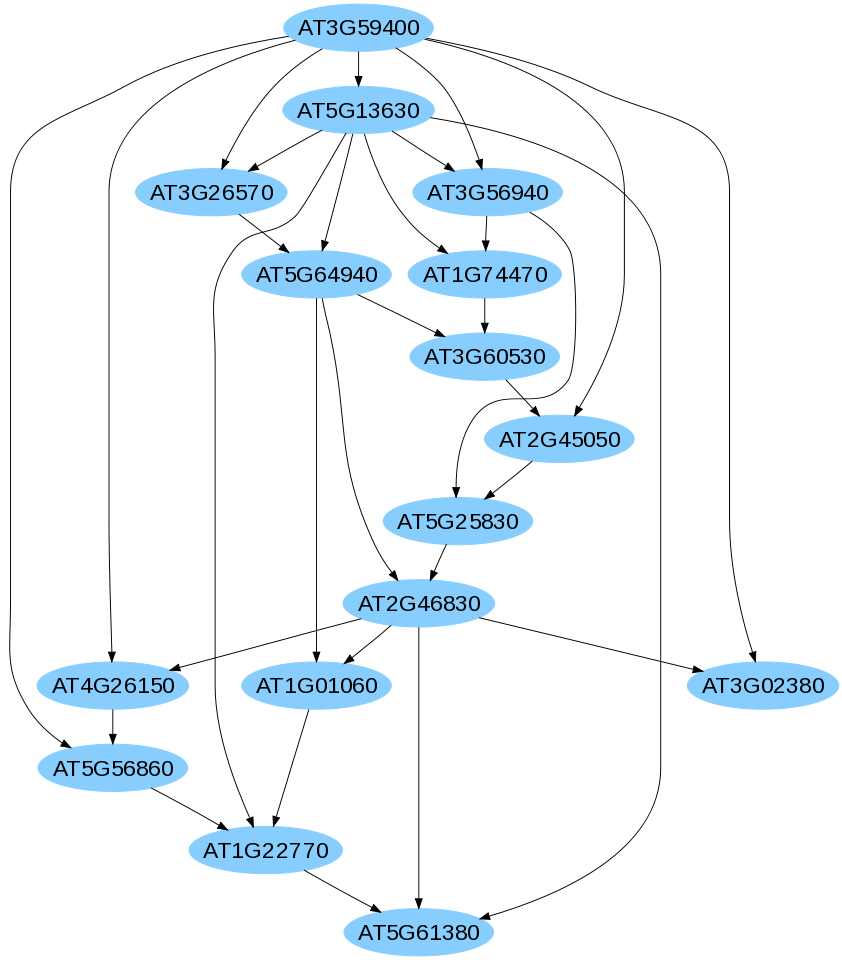

Supplement: Additional file 1 — Mini-website showing all learned network graphs for examples presented. Mini-website showing all learned network graphs at each iteration for the examples presented in the main body of the paper, and a table of the genes involved. [file 1752-0509-3-85-S1.zip › S/net3/grn_16.png]

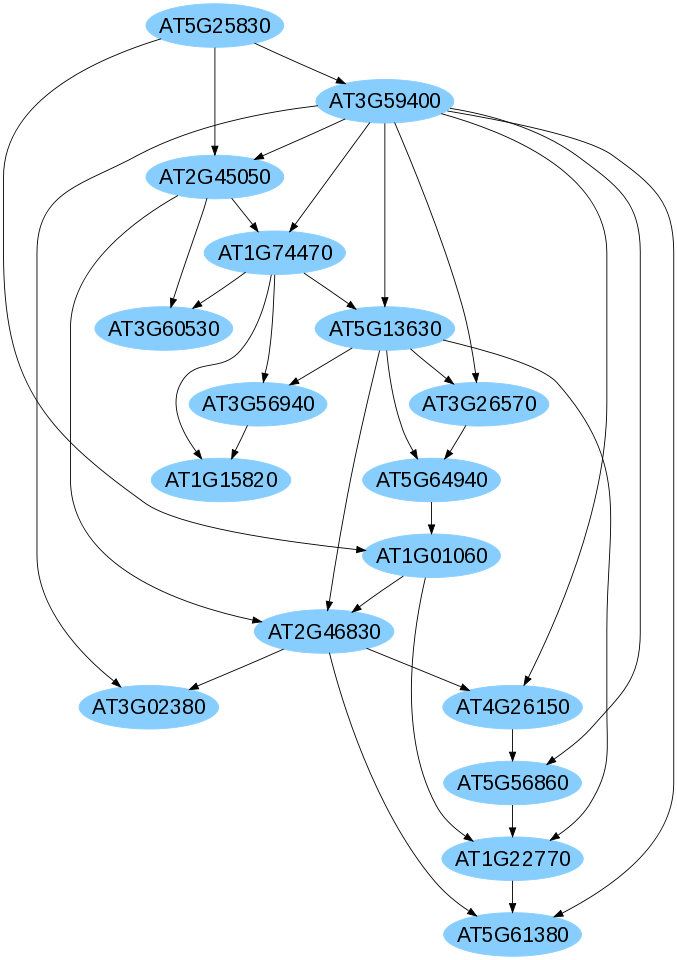

Supplement: Additional file 1 — Mini-website showing all learned network graphs for examples presented. Mini-website showing all learned network graphs at each iteration for the examples presented in the main body of the paper, and a table of the genes involved. [file 1752-0509-3-85-S1.zip › S/net3/grn_17.png]

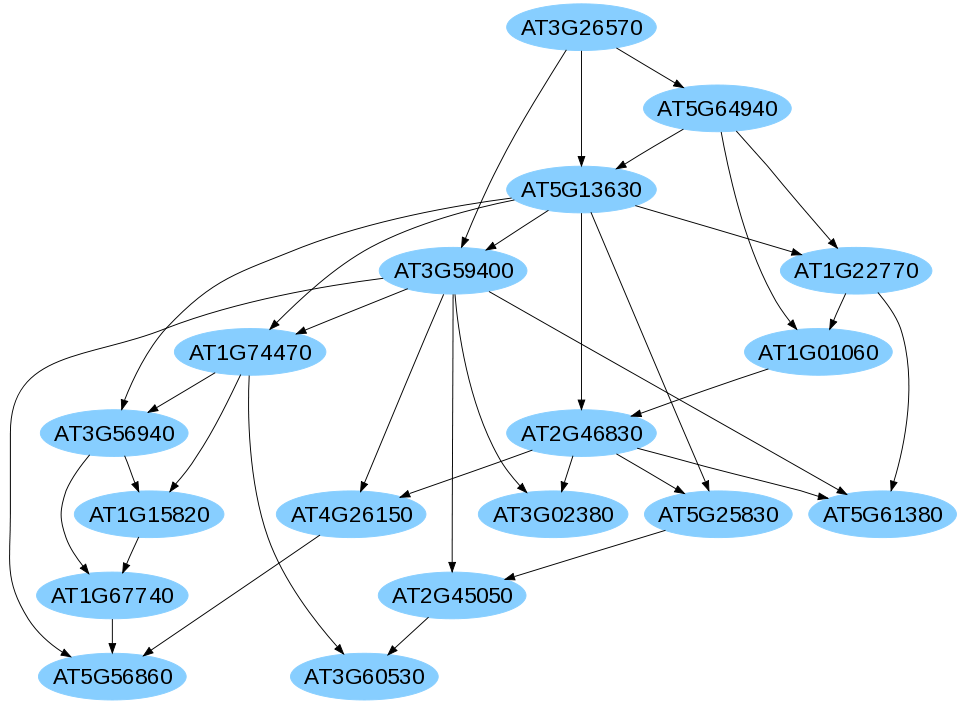

Supplement: Additional file 1 — Mini-website showing all learned network graphs for examples presented. Mini-website showing all learned network graphs at each iteration for the examples presented in the main body of the paper, and a table of the genes involved. [file 1752-0509-3-85-S1.zip › S/net3/grn_18.png]

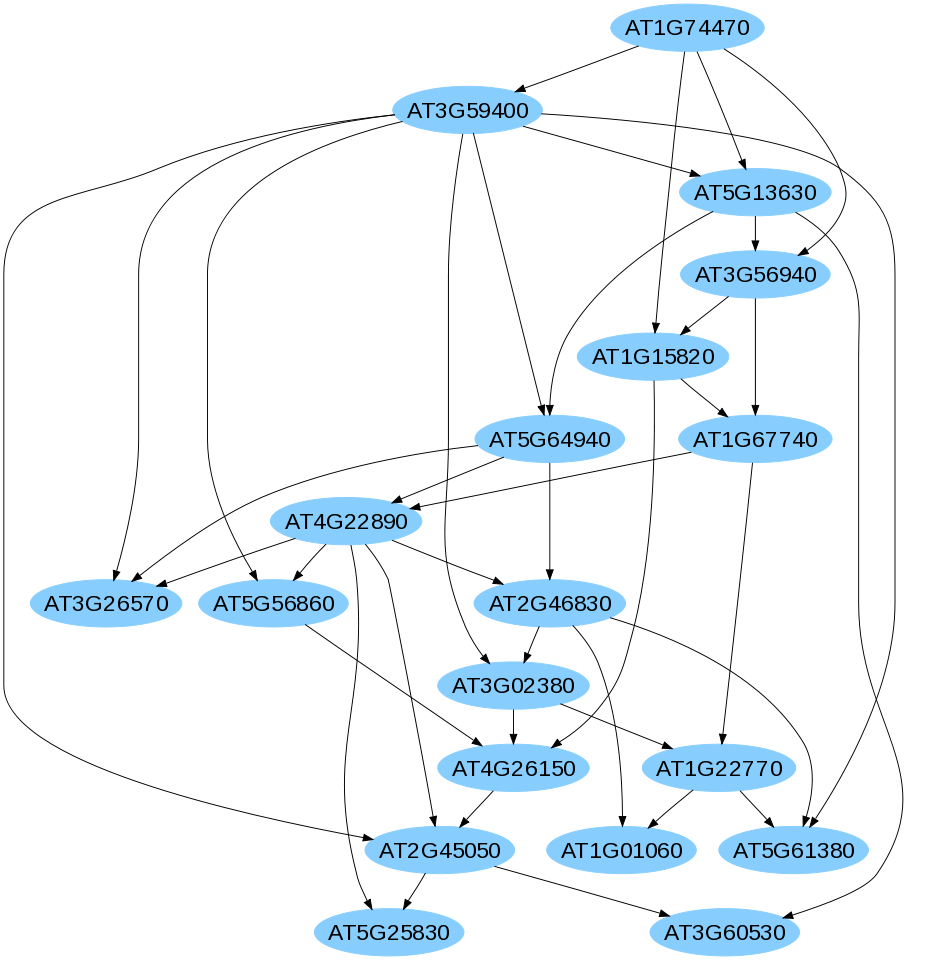

Supplement: Additional file 1 — Mini-website showing all learned network graphs for examples presented. Mini-website showing all learned network graphs at each iteration for the examples presented in the main body of the paper, and a table of the genes involved. [file 1752-0509-3-85-S1.zip › S/net3/grn_19.png]

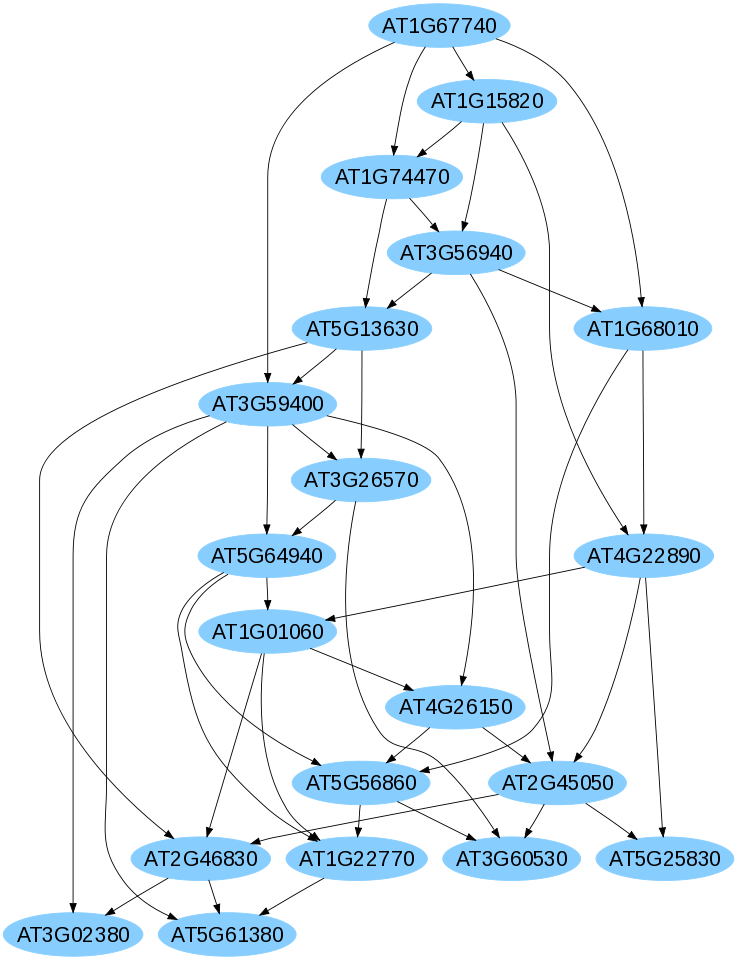

Supplement: Additional file 1 — Mini-website showing all learned network graphs for examples presented. Mini-website showing all learned network graphs at each iteration for the examples presented in the main body of the paper, and a table of the genes involved. [file 1752-0509-3-85-S1.zip › S/net3/grn_20.png]

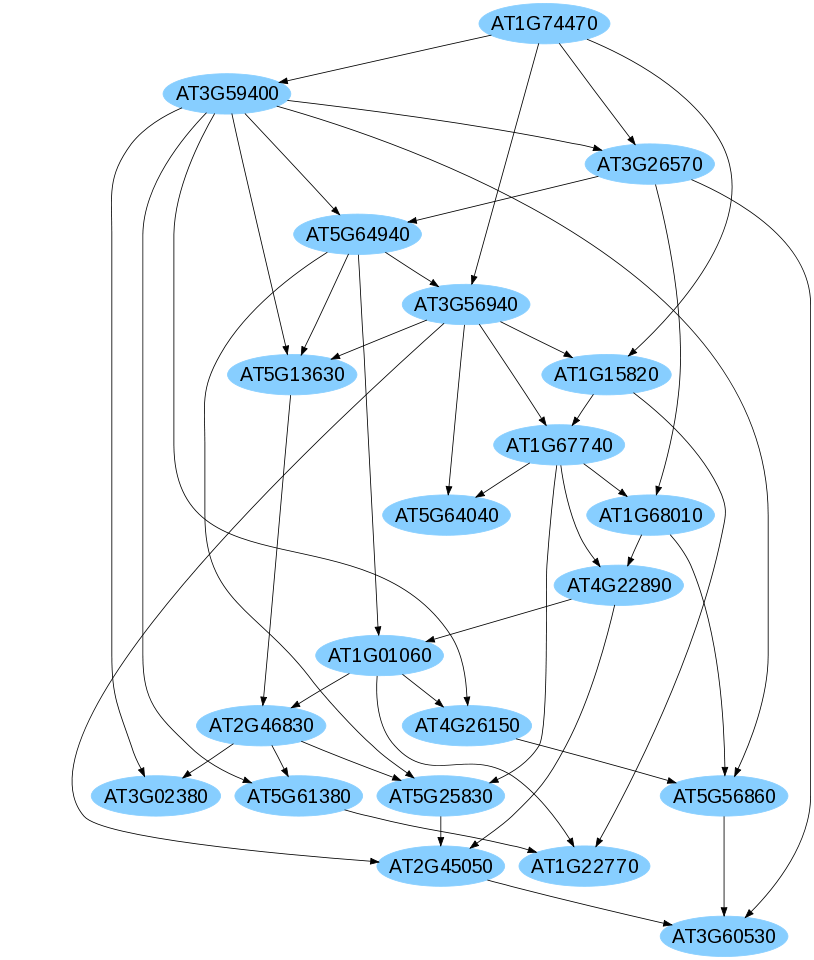

Supplement: Additional file 1 — Mini-website showing all learned network graphs for examples presented. Mini-website showing all learned network graphs at each iteration for the examples presented in the main body of the paper, and a table of the genes involved. [file 1752-0509-3-85-S1.zip › S/net3/grn_21.png]

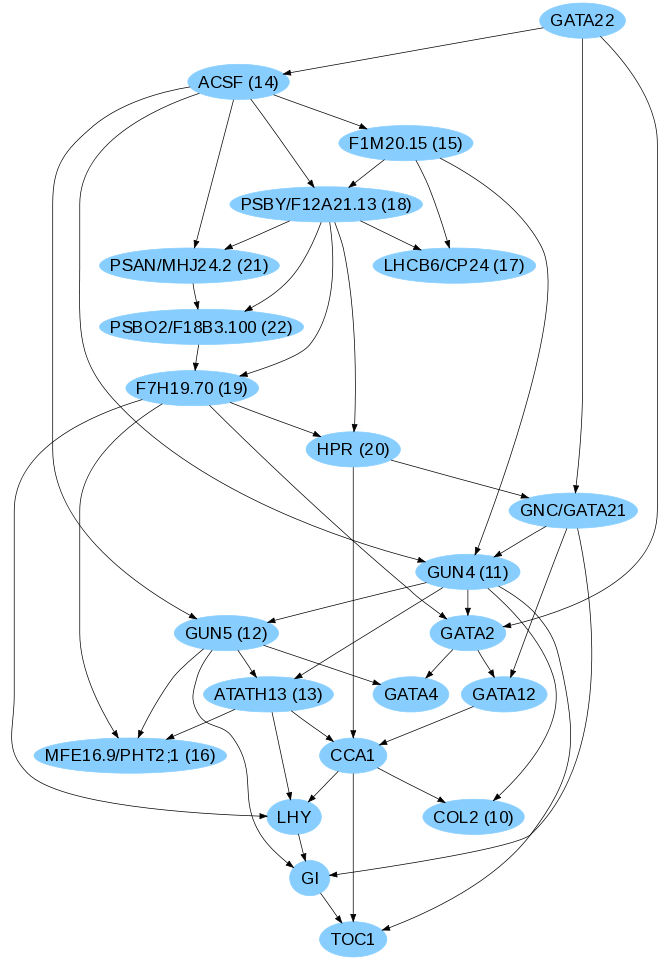

Supplement: Additional file 1 — Mini-website showing all learned network graphs for examples presented. Mini-website showing all learned network graphs at each iteration for the examples presented in the main body of the paper, and a table of the genes involved. [file 1752-0509-3-85-S1.zip › S/net3/grn_22genesymbols.png]

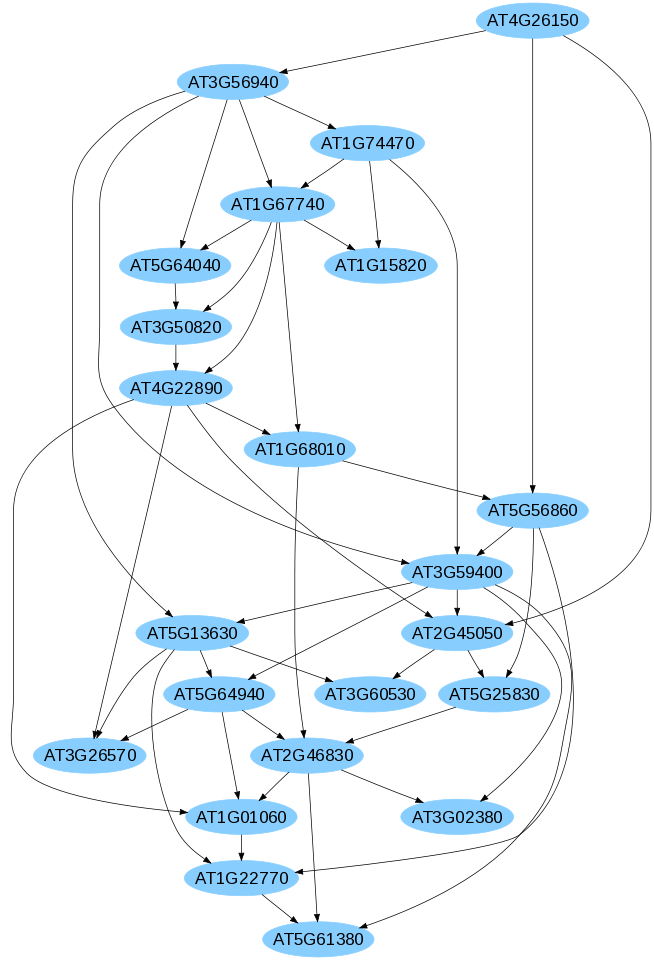

Supplement: Additional file 1 — Mini-website showing all learned network graphs for examples presented. Mini-website showing all learned network graphs at each iteration for the examples presented in the main body of the paper, and a table of the genes involved. [file 1752-0509-3-85-S1.zip › S/net3/grn_22.png]

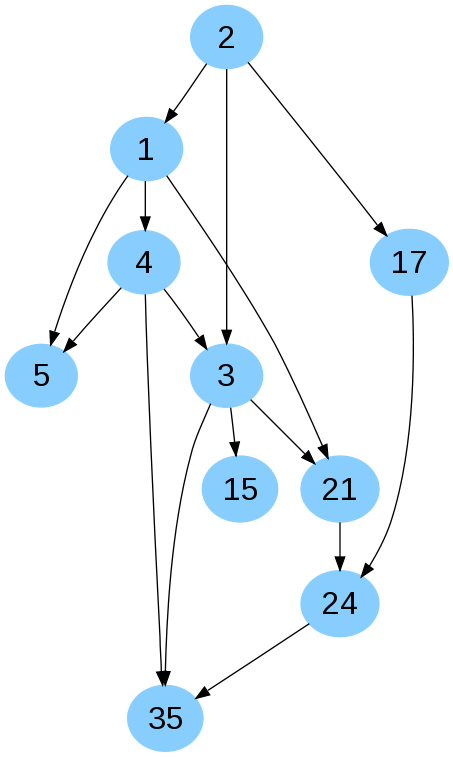

Supplement: Additional file 1 — Mini-website showing all learned network graphs for examples presented. Mini-website showing all learned network graphs at each iteration for the examples presented in the main body of the paper, and a table of the genes involved. [file 1752-0509-3-85-S1.zip › S/net4/grn_10.png]

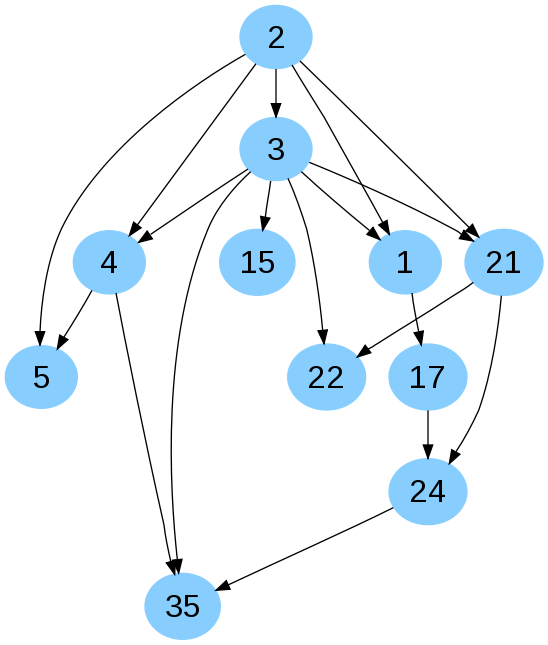

Supplement: Additional file 1 — Mini-website showing all learned network graphs for examples presented. Mini-website showing all learned network graphs at each iteration for the examples presented in the main body of the paper, and a table of the genes involved. [file 1752-0509-3-85-S1.zip › S/net4/grn_11.png]

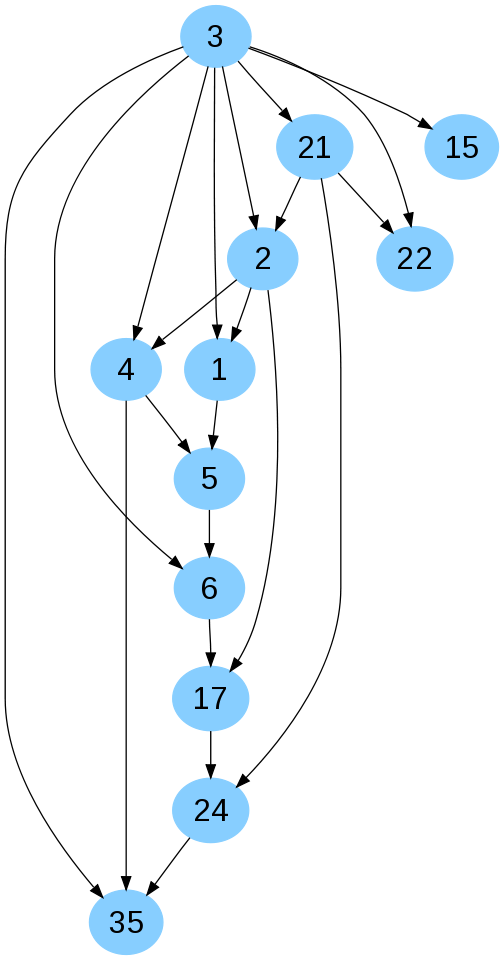

Supplement: Additional file 1 — Mini-website showing all learned network graphs for examples presented. Mini-website showing all learned network graphs at each iteration for the examples presented in the main body of the paper, and a table of the genes involved. [file 1752-0509-3-85-S1.zip › S/net4/grn_12.png]

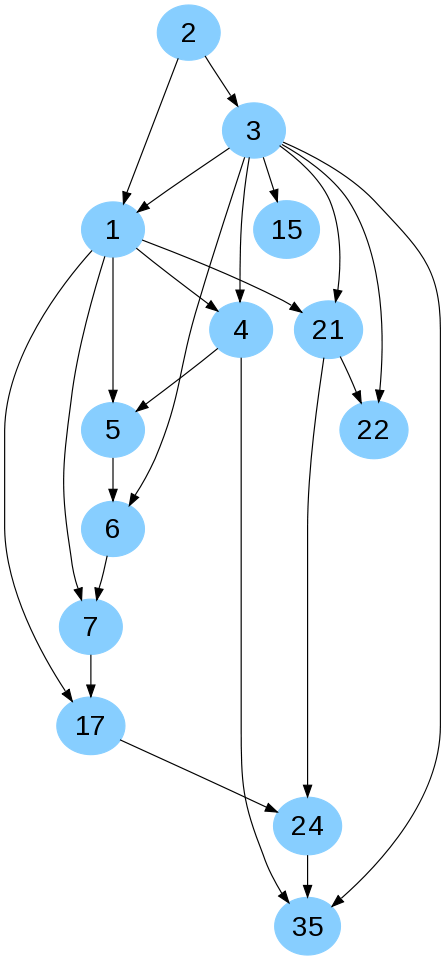

Supplement: Additional file 1 — Mini-website showing all learned network graphs for examples presented. Mini-website showing all learned network graphs at each iteration for the examples presented in the main body of the paper, and a table of the genes involved. [file 1752-0509-3-85-S1.zip › S/net4/grn_13.png]

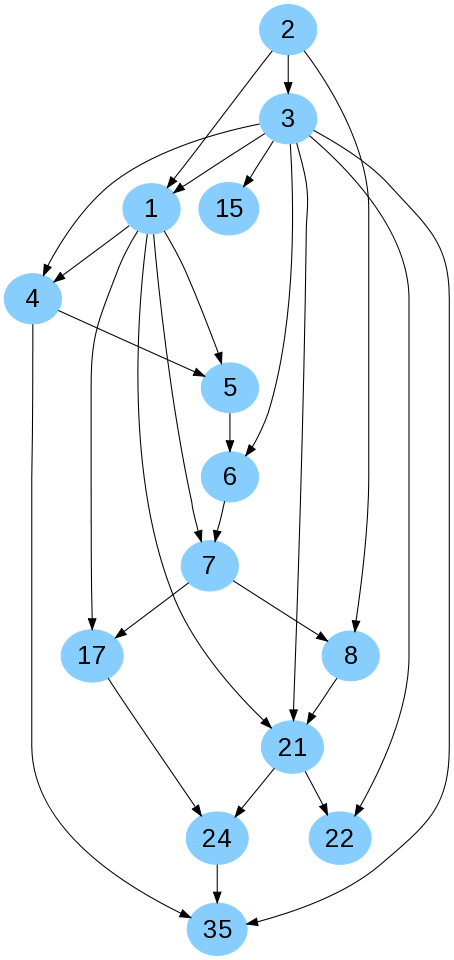

Supplement: Additional file 1 — Mini-website showing all learned network graphs for examples presented. Mini-website showing all learned network graphs at each iteration for the examples presented in the main body of the paper, and a table of the genes involved. [file 1752-0509-3-85-S1.zip › S/net4/grn_14.png]

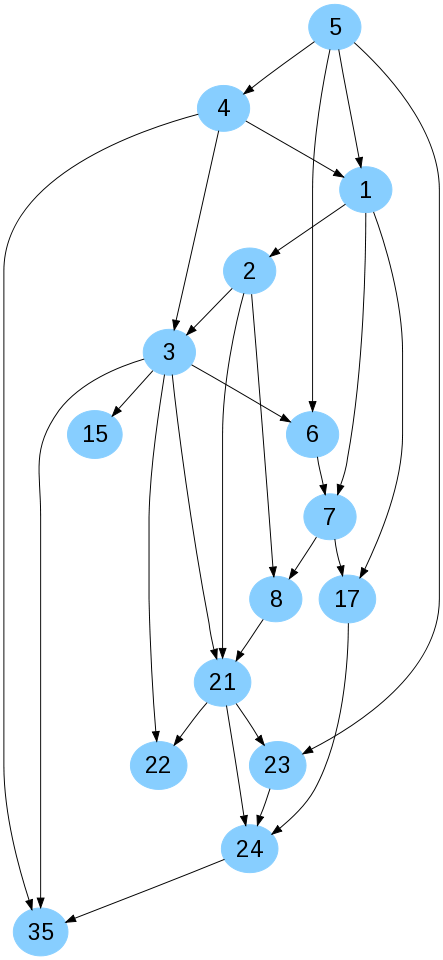

Supplement: Additional file 1 — Mini-website showing all learned network graphs for examples presented. Mini-website showing all learned network graphs at each iteration for the examples presented in the main body of the paper, and a table of the genes involved. [file 1752-0509-3-85-S1.zip › S/net4/grn_15.png]

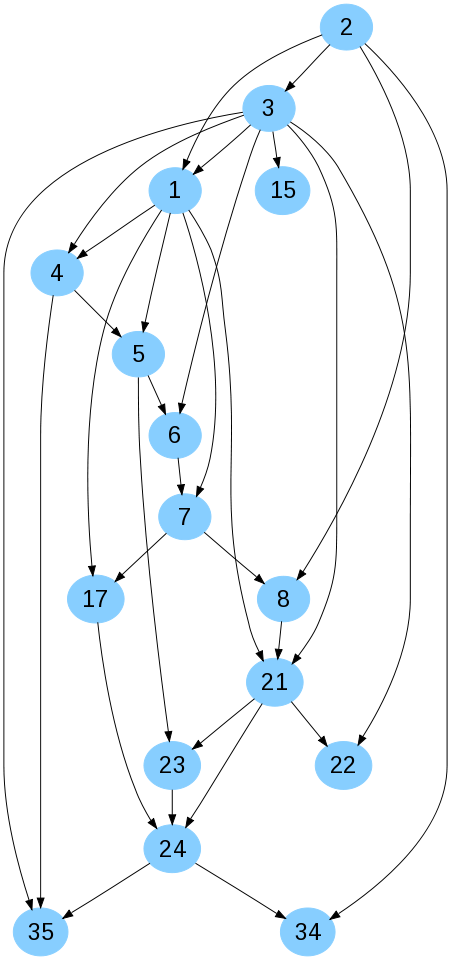

Supplement: Additional file 1 — Mini-website showing all learned network graphs for examples presented. Mini-website showing all learned network graphs at each iteration for the examples presented in the main body of the paper, and a table of the genes involved. [file 1752-0509-3-85-S1.zip › S/net4/grn_16.png]

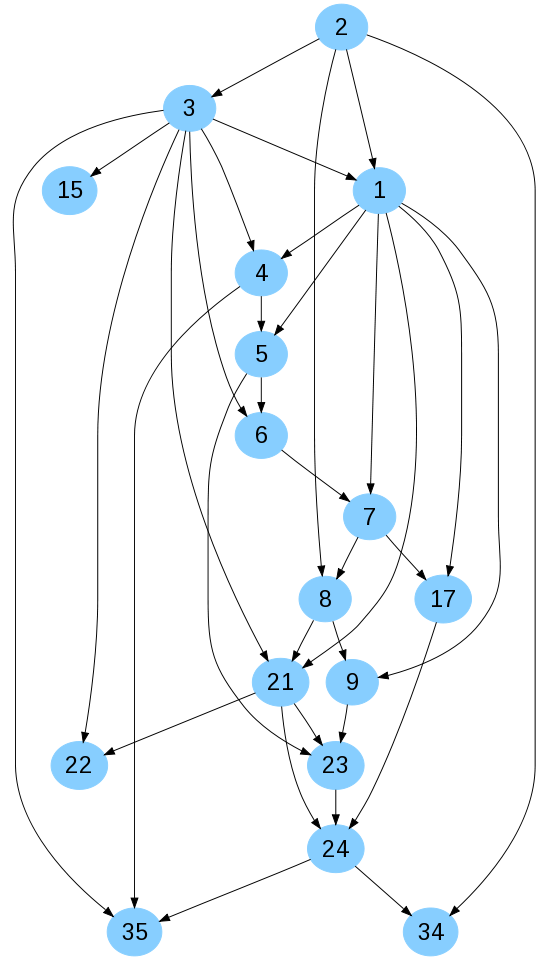

Supplement: Additional file 1 — Mini-website showing all learned network graphs for examples presented. Mini-website showing all learned network graphs at each iteration for the examples presented in the main body of the paper, and a table of the genes involved. [file 1752-0509-3-85-S1.zip › S/net4/grn_17.png]

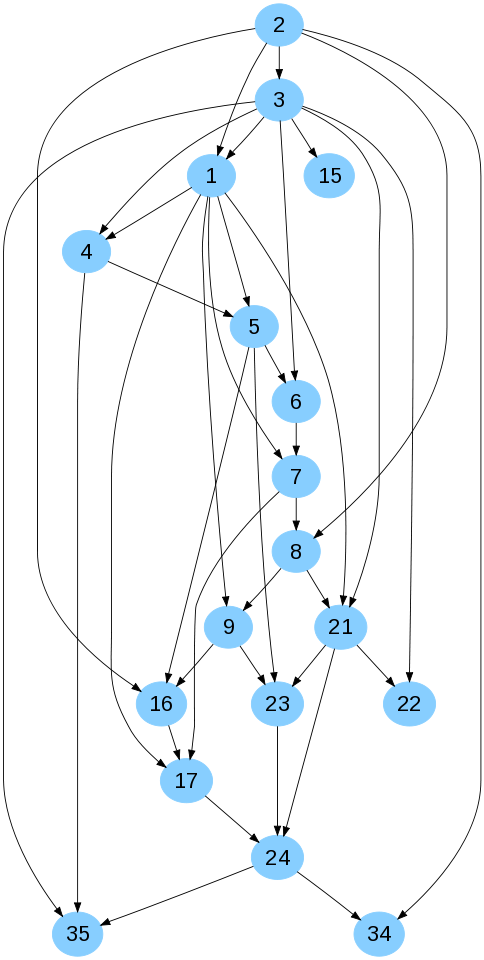

Supplement: Additional file 1 — Mini-website showing all learned network graphs for examples presented. Mini-website showing all learned network graphs at each iteration for the examples presented in the main body of the paper, and a table of the genes involved. [file 1752-0509-3-85-S1.zip › S/net4/grn_18.png]

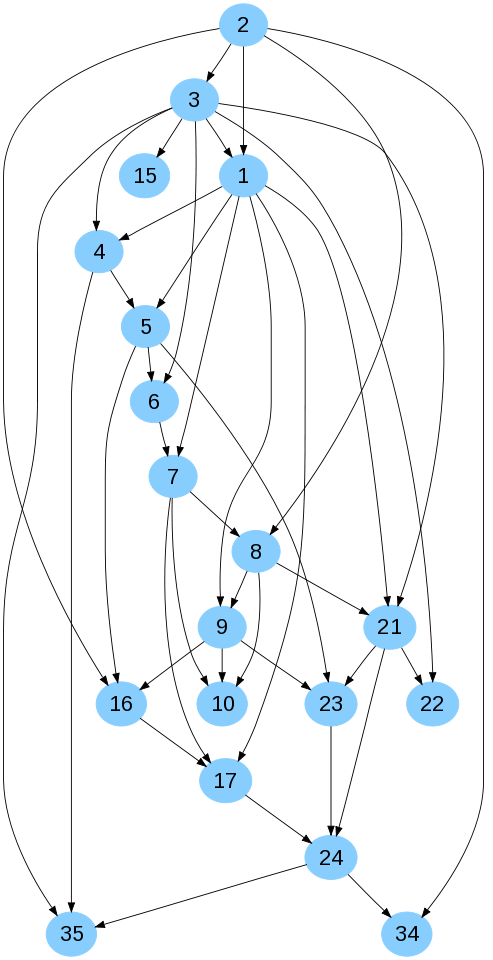

Supplement: Additional file 1 — Mini-website showing all learned network graphs for examples presented. Mini-website showing all learned network graphs at each iteration for the examples presented in the main body of the paper, and a table of the genes involved. [file 1752-0509-3-85-S1.zip › S/net4/grn_19.png]

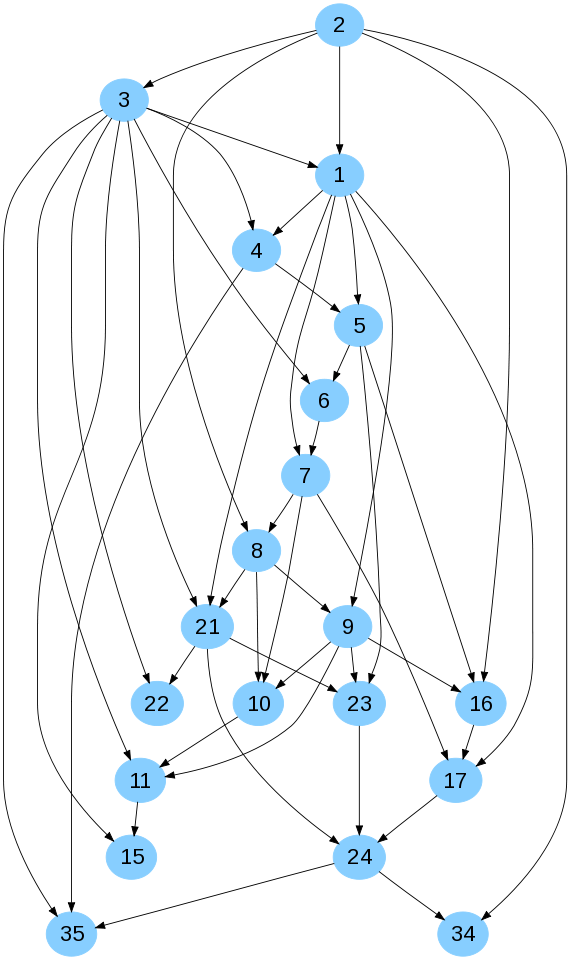

Supplement: Additional file 1 — Mini-website showing all learned network graphs for examples presented. Mini-website showing all learned network graphs at each iteration for the examples presented in the main body of the paper, and a table of the genes involved. [file 1752-0509-3-85-S1.zip › S/net4/grn_20.png]

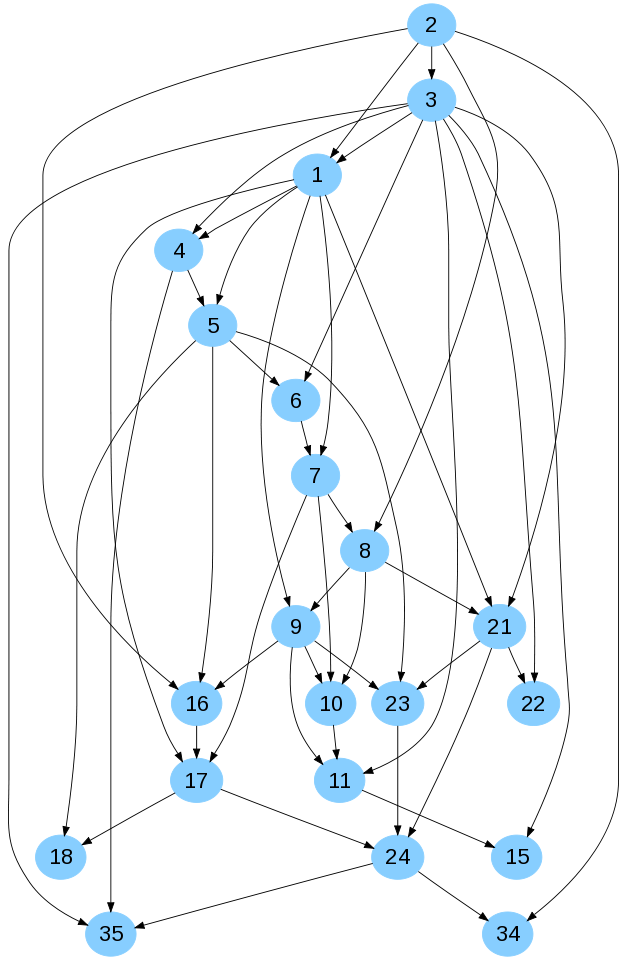

Supplement: Additional file 1 — Mini-website showing all learned network graphs for examples presented. Mini-website showing all learned network graphs at each iteration for the examples presented in the main body of the paper, and a table of the genes involved. [file 1752-0509-3-85-S1.zip › S/net4/grn_21.png]

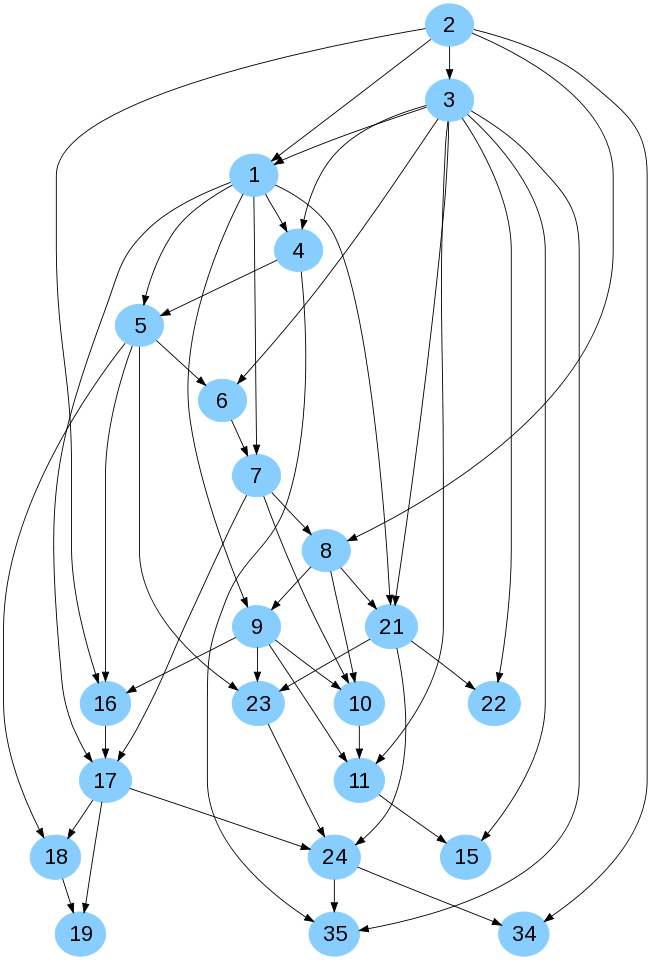

Supplement: Additional file 1 — Mini-website showing all learned network graphs for examples presented. Mini-website showing all learned network graphs at each iteration for the examples presented in the main body of the paper, and a table of the genes involved. [file 1752-0509-3-85-S1.zip › S/net4/grn_22.png]

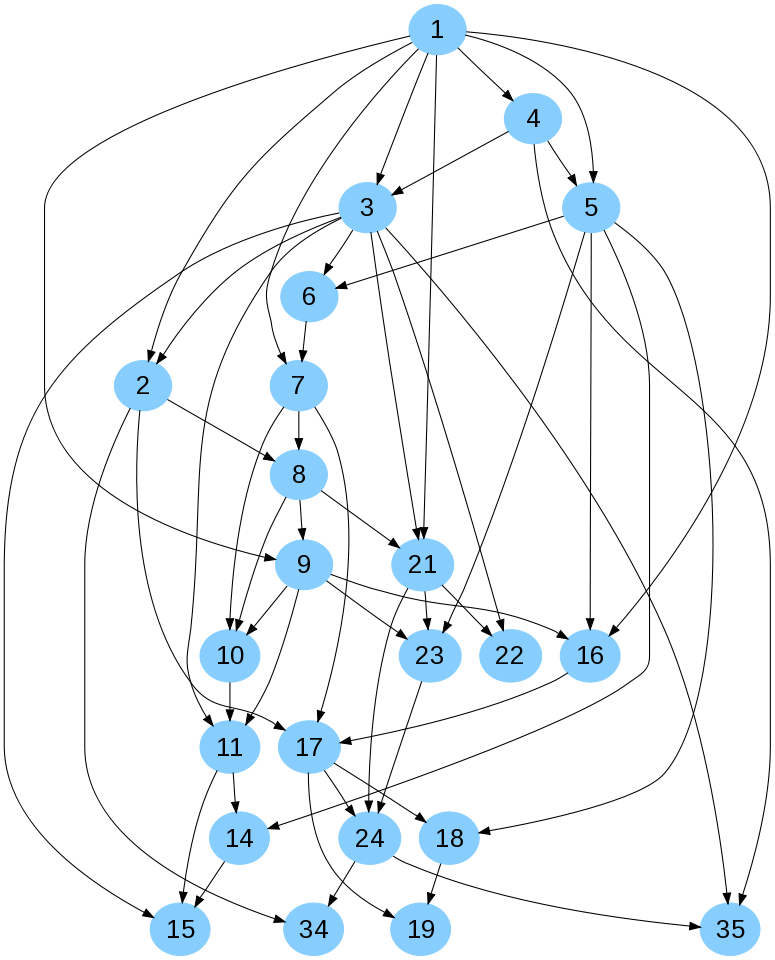

Supplement: Additional file 1 — Mini-website showing all learned network graphs for examples presented. Mini-website showing all learned network graphs at each iteration for the examples presented in the main body of the paper, and a table of the genes involved. [file 1752-0509-3-85-S1.zip › S/net4/grn_23.png]

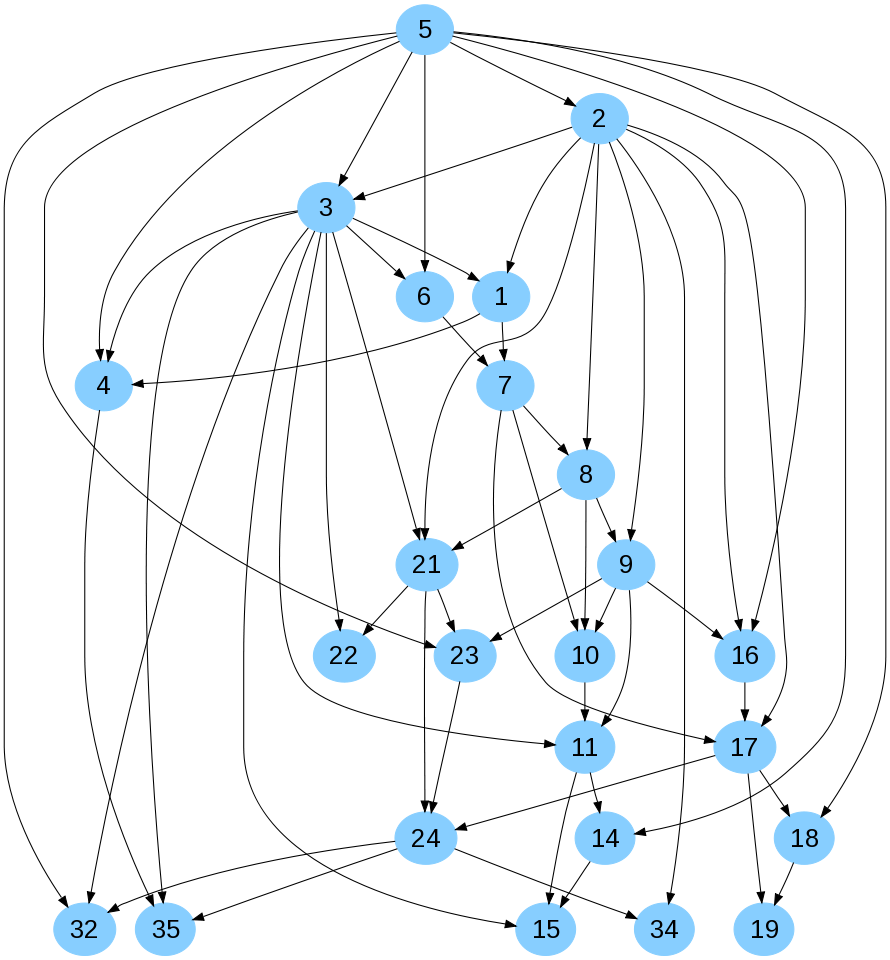

Supplement: Additional file 1 — Mini-website showing all learned network graphs for examples presented. Mini-website showing all learned network graphs at each iteration for the examples presented in the main body of the paper, and a table of the genes involved. [file 1752-0509-3-85-S1.zip › S/net4/grn_24.png]

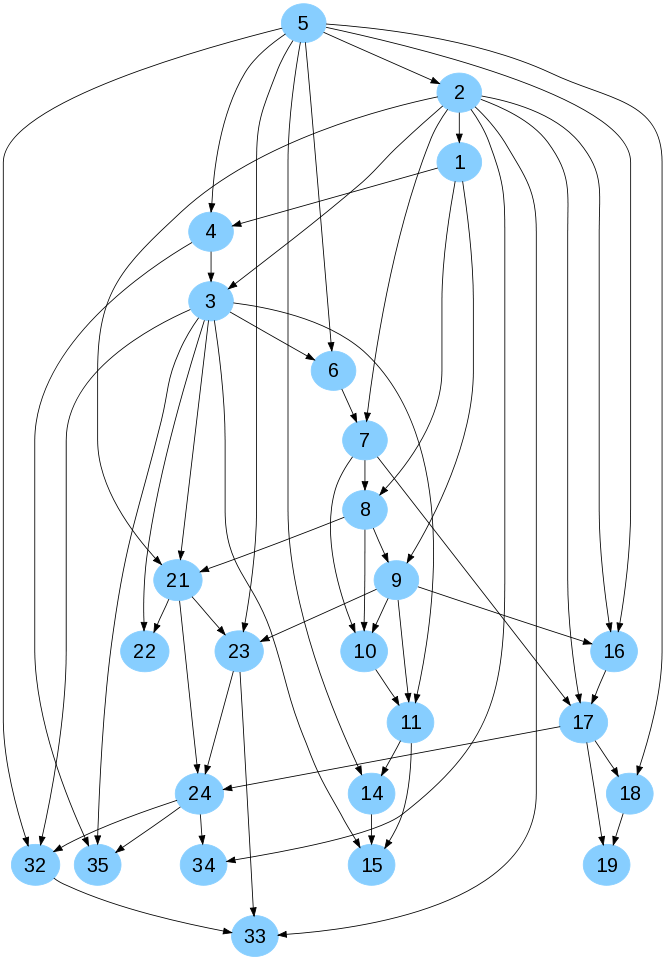

Supplement: Additional file 1 — Mini-website showing all learned network graphs for examples presented. Mini-website showing all learned network graphs at each iteration for the examples presented in the main body of the paper, and a table of the genes involved. [file 1752-0509-3-85-S1.zip › S/net4/grn_25.png]

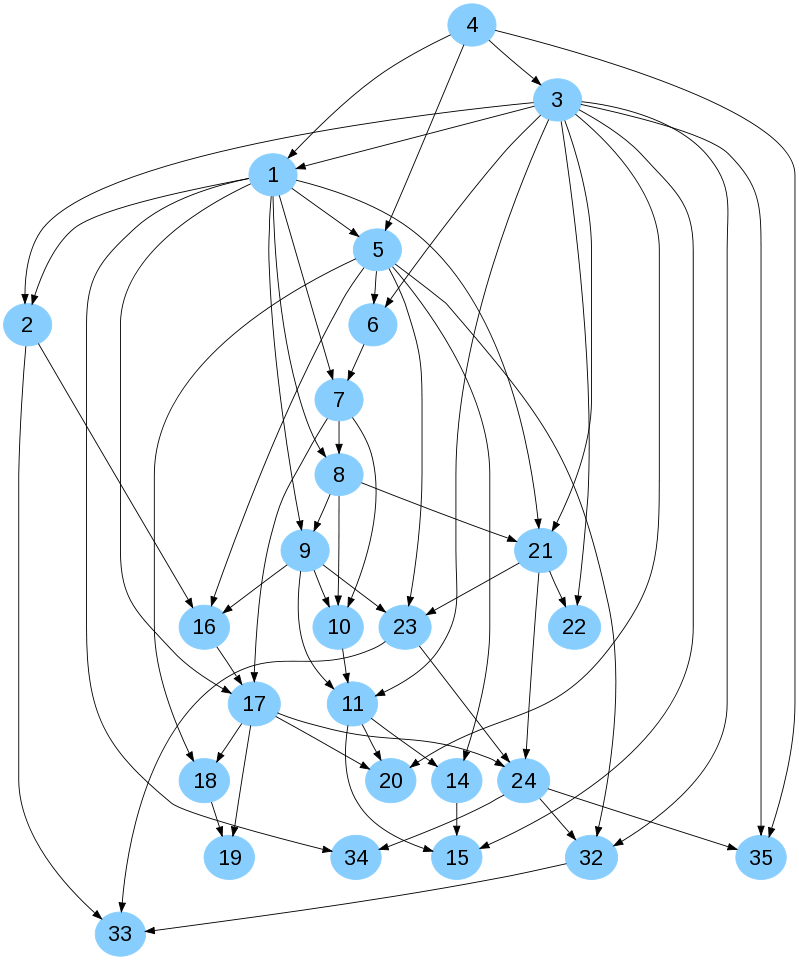

Supplement: Additional file 1 — Mini-website showing all learned network graphs for examples presented. Mini-website showing all learned network graphs at each iteration for the examples presented in the main body of the paper, and a table of the genes involved. [file 1752-0509-3-85-S1.zip › S/net4/grn_26.png]

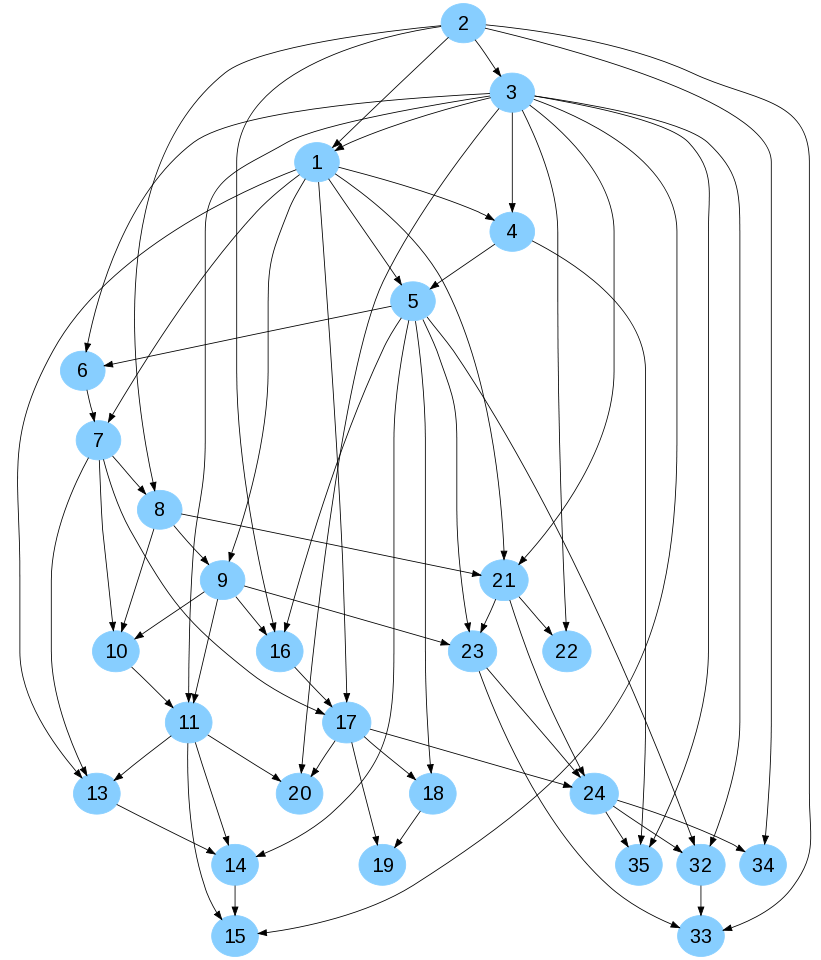

Supplement: Additional file 1 — Mini-website showing all learned network graphs for examples presented. Mini-website showing all learned network graphs at each iteration for the examples presented in the main body of the paper, and a table of the genes involved. [file 1752-0509-3-85-S1.zip › S/net4/grn_27.png]

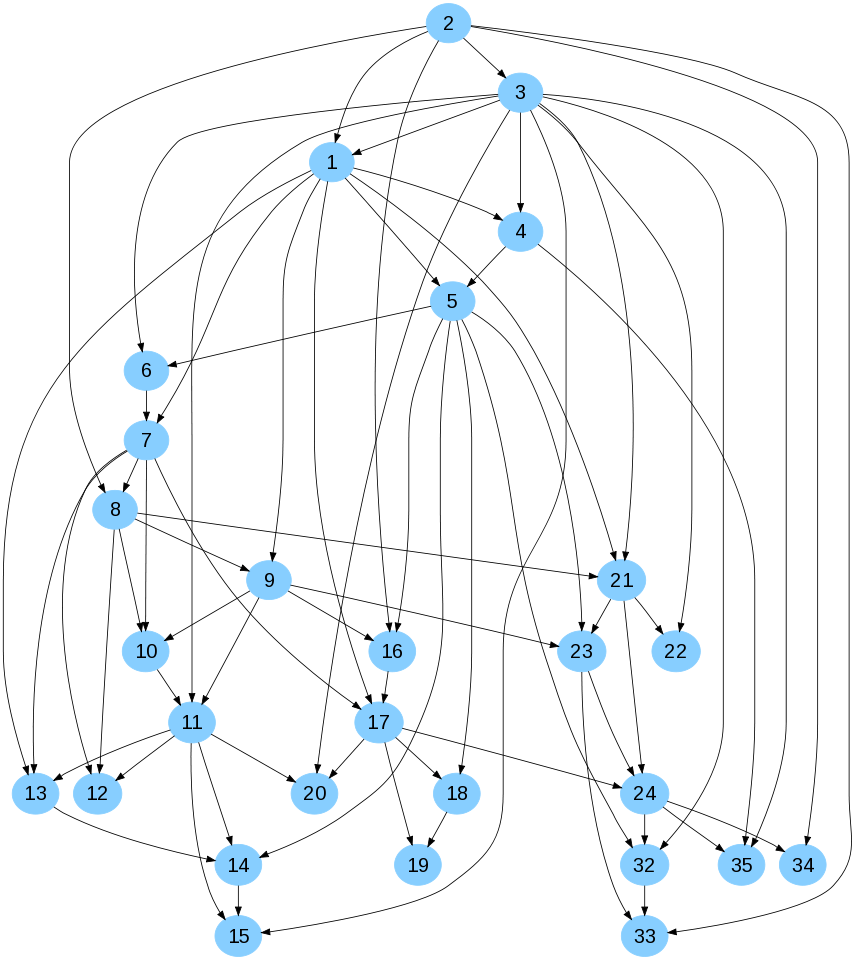

Supplement: Additional file 1 — Mini-website showing all learned network graphs for examples presented. Mini-website showing all learned network graphs at each iteration for the examples presented in the main body of the paper, and a table of the genes involved. [file 1752-0509-3-85-S1.zip › S/net4/grn_28.png]

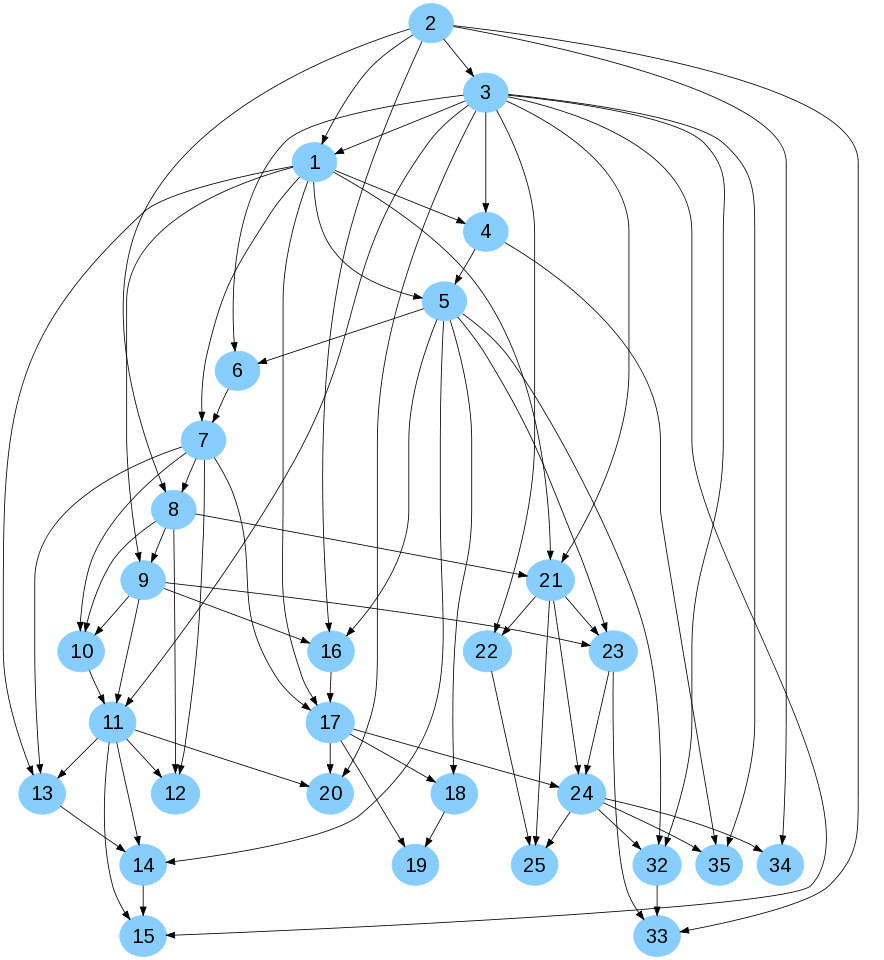

Supplement: Additional file 1 — Mini-website showing all learned network graphs for examples presented. Mini-website showing all learned network graphs at each iteration for the examples presented in the main body of the paper, and a table of the genes involved. [file 1752-0509-3-85-S1.zip › S/net4/grn_29.png]

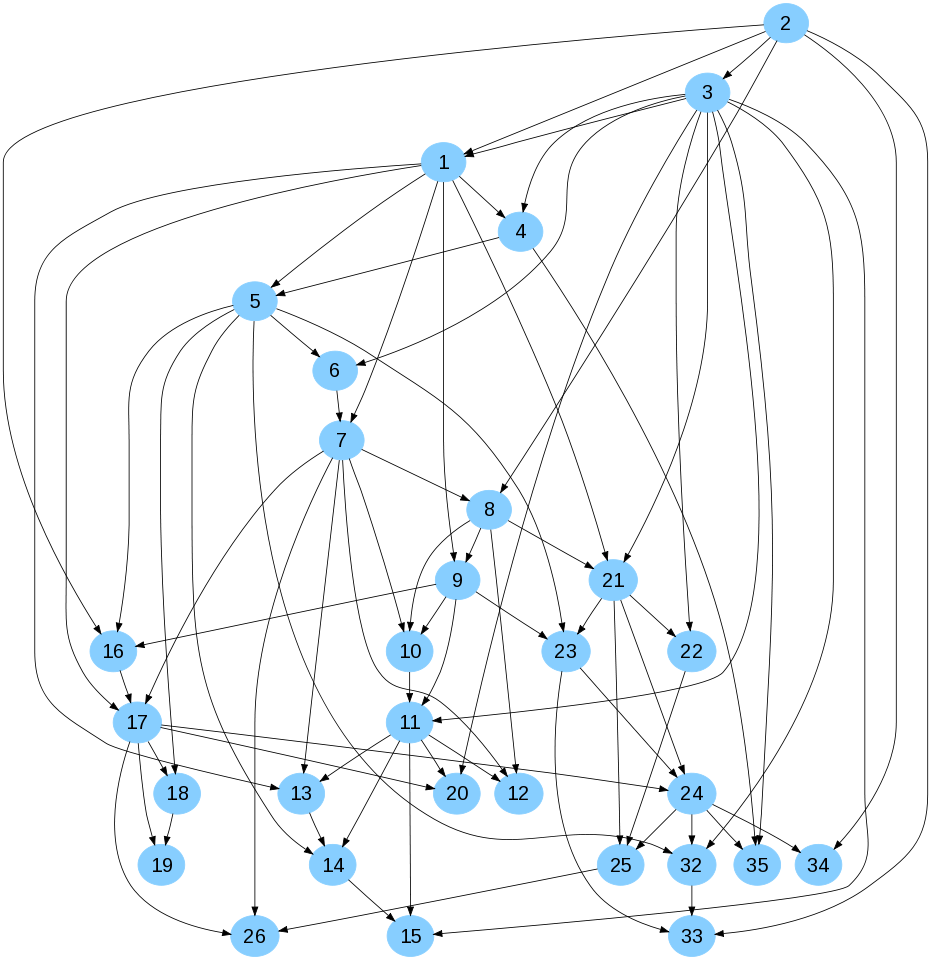

Supplement: Additional file 1 — Mini-website showing all learned network graphs for examples presented. Mini-website showing all learned network graphs at each iteration for the examples presented in the main body of the paper, and a table of the genes involved. [file 1752-0509-3-85-S1.zip › S/net4/grn_30.png]

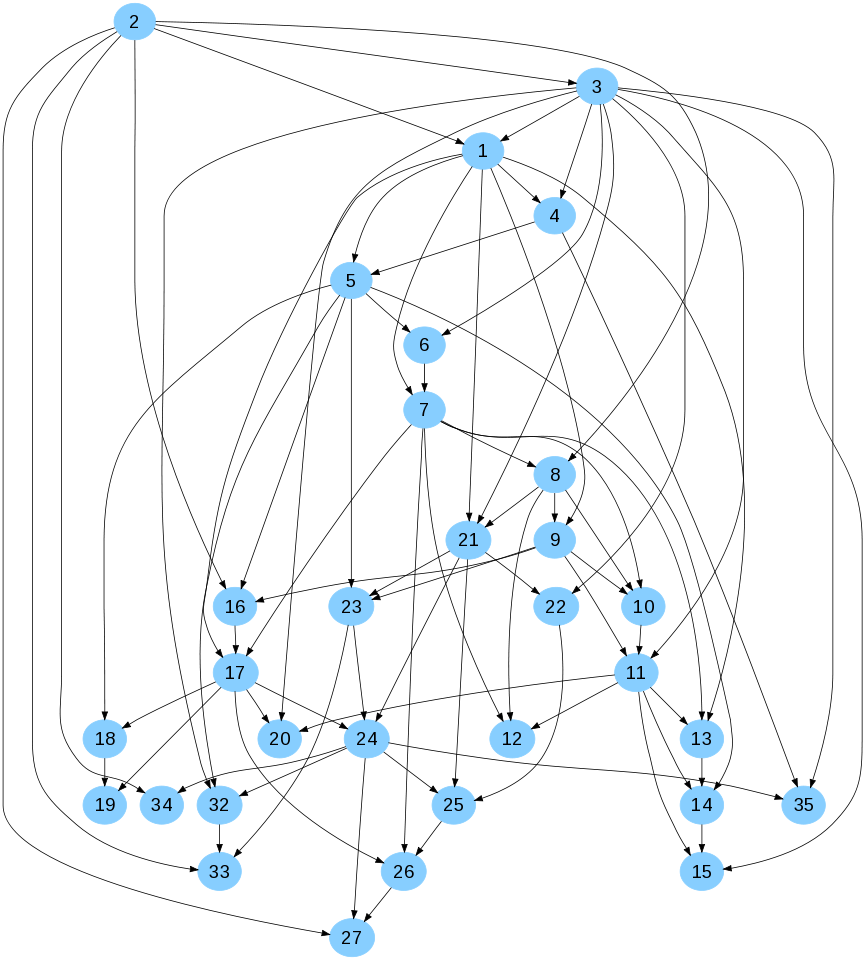

Supplement: Additional file 1 — Mini-website showing all learned network graphs for examples presented. Mini-website showing all learned network graphs at each iteration for the examples presented in the main body of the paper, and a table of the genes involved. [file 1752-0509-3-85-S1.zip › S/net4/grn_31.png]

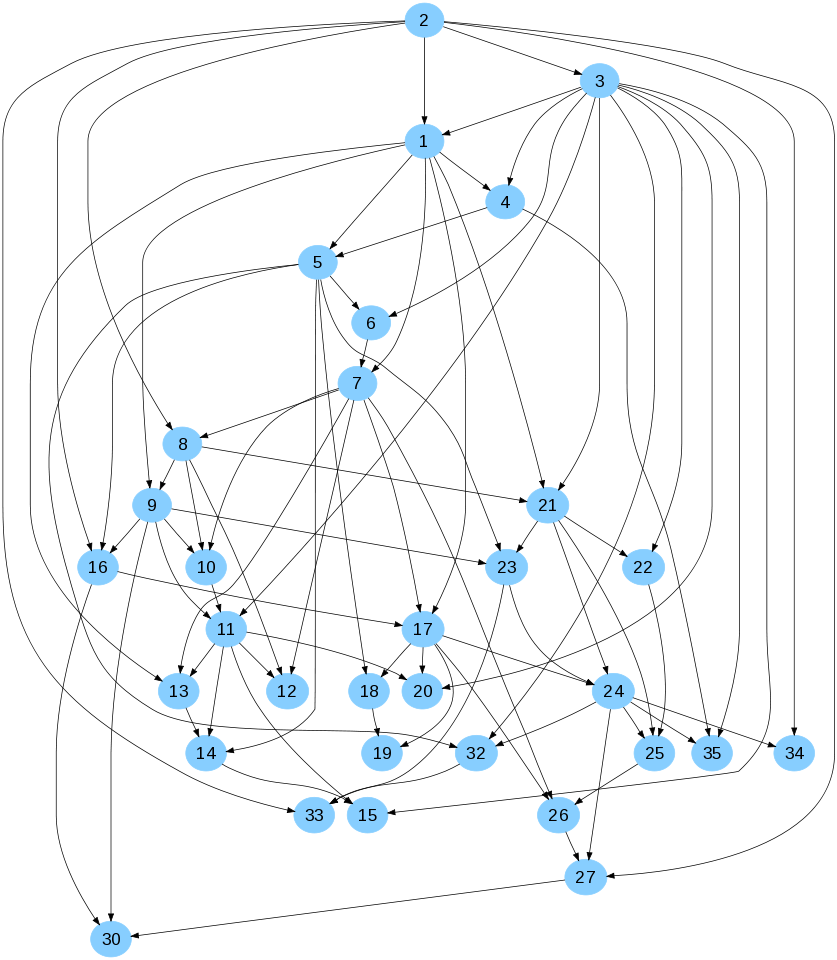

Supplement: Additional file 1 — Mini-website showing all learned network graphs for examples presented. Mini-website showing all learned network graphs at each iteration for the examples presented in the main body of the paper, and a table of the genes involved. [file 1752-0509-3-85-S1.zip › S/net4/grn_32.png]

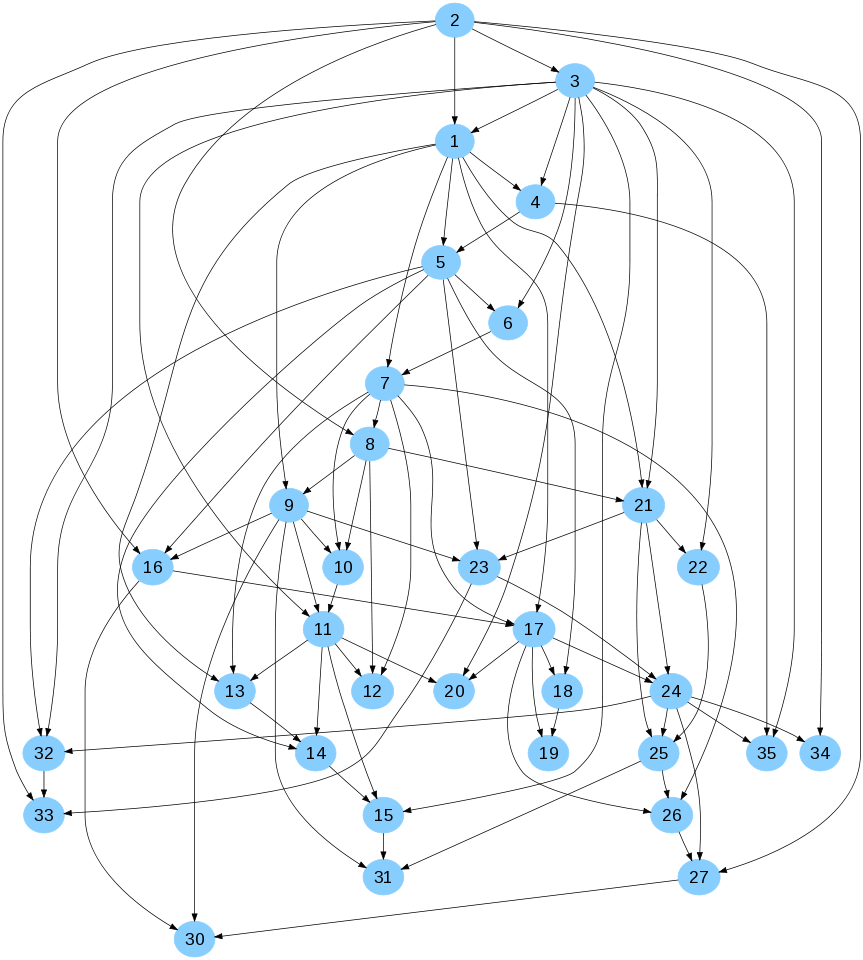

Supplement: Additional file 1 — Mini-website showing all learned network graphs for examples presented. Mini-website showing all learned network graphs at each iteration for the examples presented in the main body of the paper, and a table of the genes involved. [file 1752-0509-3-85-S1.zip › S/net4/grn_33.png]

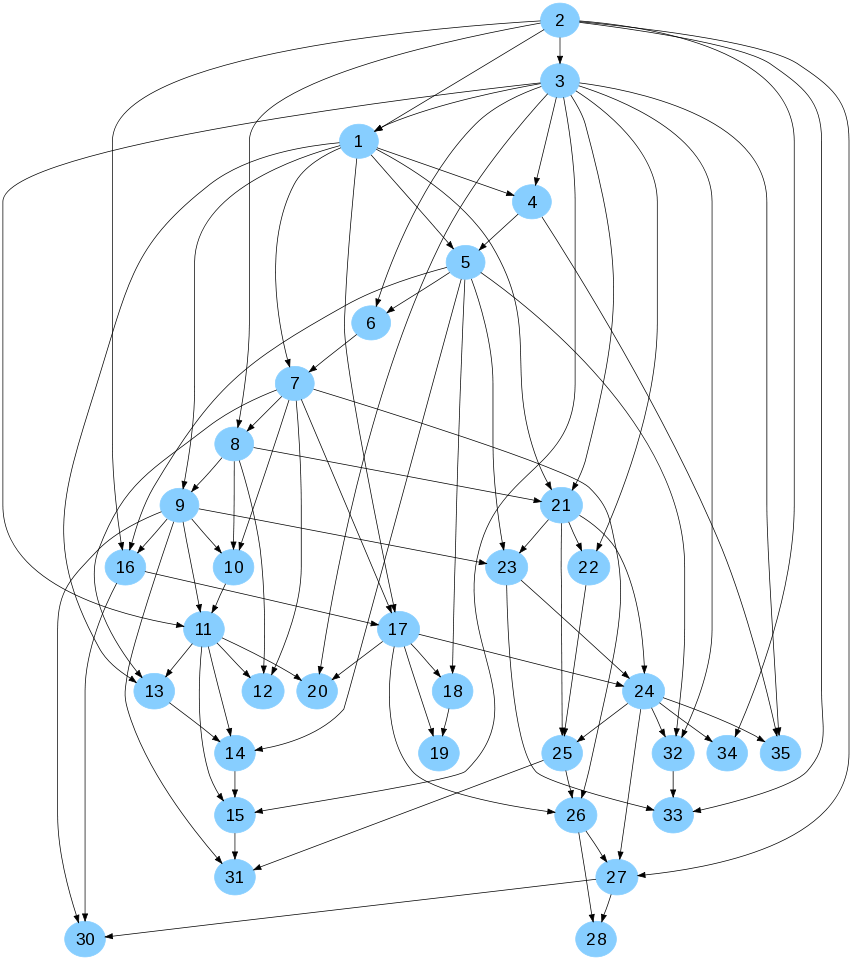

Supplement: Additional file 1 — Mini-website showing all learned network graphs for examples presented. Mini-website showing all learned network graphs at each iteration for the examples presented in the main body of the paper, and a table of the genes involved. [file 1752-0509-3-85-S1.zip › S/net4/grn_34.png]

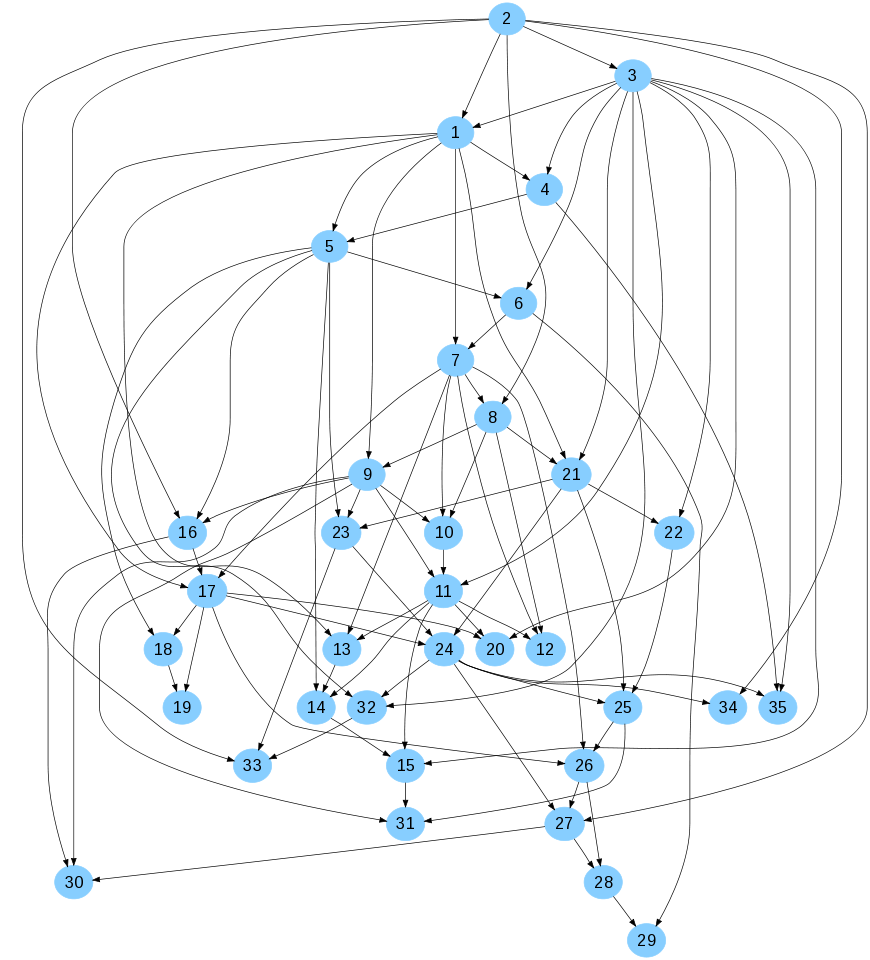

Supplement: Additional file 1 — Mini-website showing all learned network graphs for examples presented. Mini-website showing all learned network graphs at each iteration for the examples presented in the main body of the paper, and a table of the genes involved. [file 1752-0509-3-85-S1.zip › S/net4/grn_35.png]

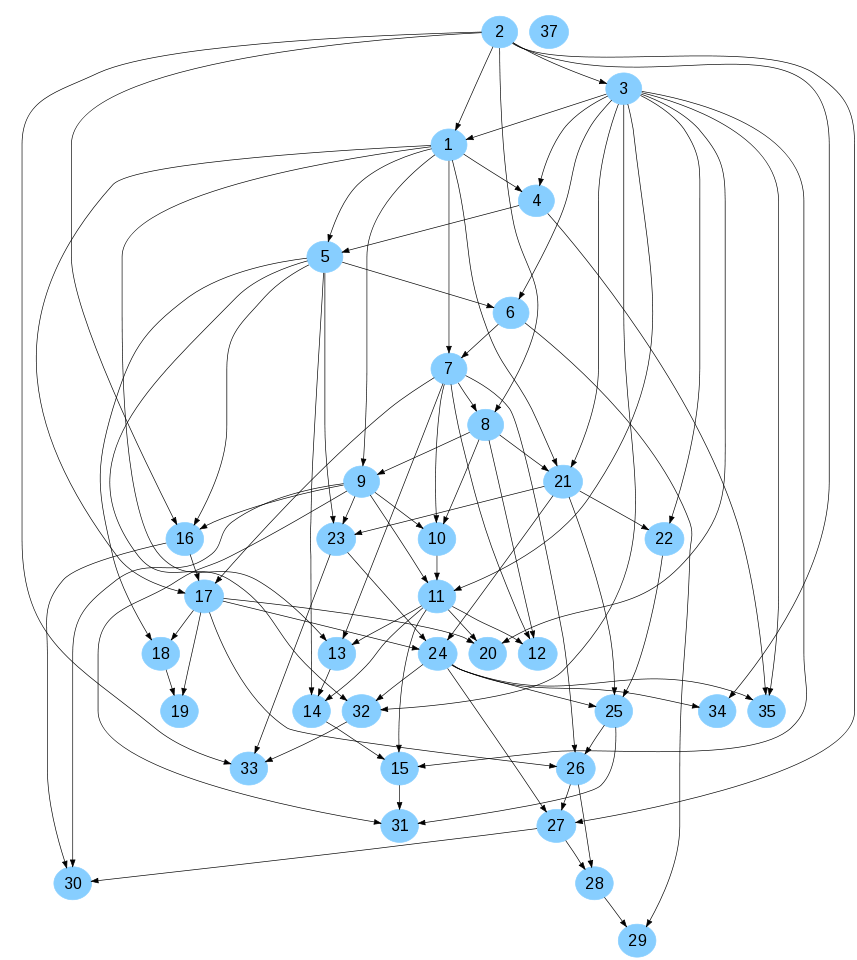

Supplement: Additional file 1 — Mini-website showing all learned network graphs for examples presented. Mini-website showing all learned network graphs at each iteration for the examples presented in the main body of the paper, and a table of the genes involved. [file 1752-0509-3-85-S1.zip › S/net4/grn_36.png]

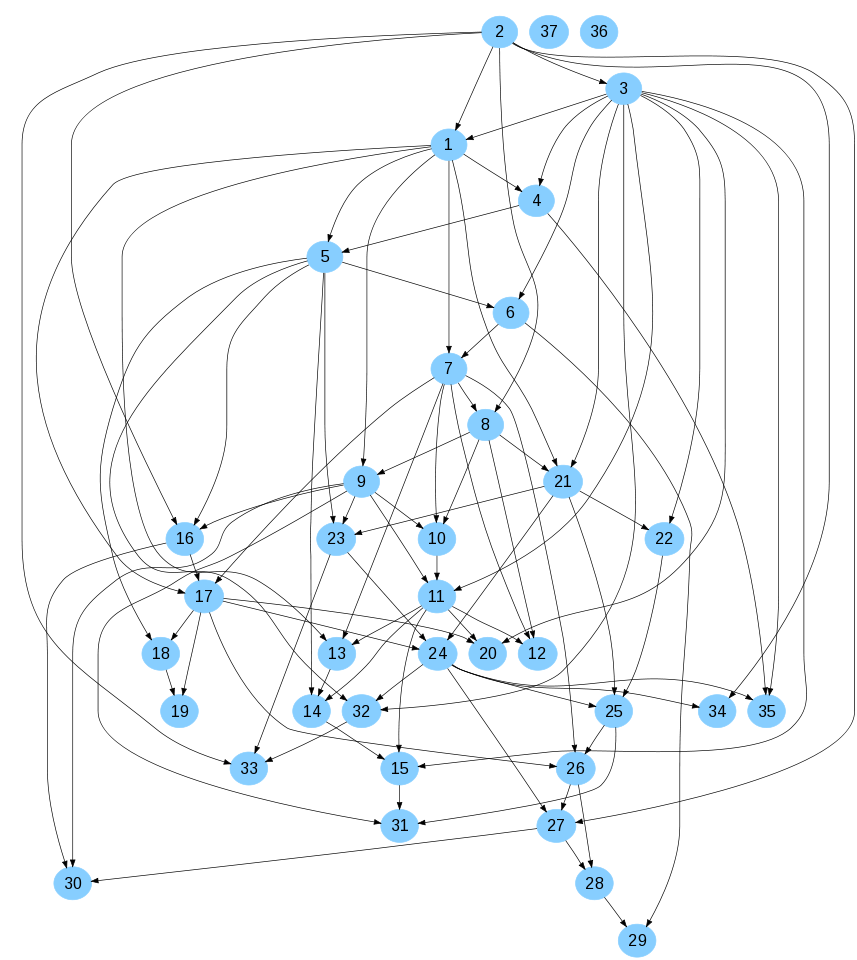

Supplement: Additional file 1 — Mini-website showing all learned network graphs for examples presented. Mini-website showing all learned network graphs at each iteration for the examples presented in the main body of the paper, and a table of the genes involved. [file 1752-0509-3-85-S1.zip › S/net4/grn_37.png]

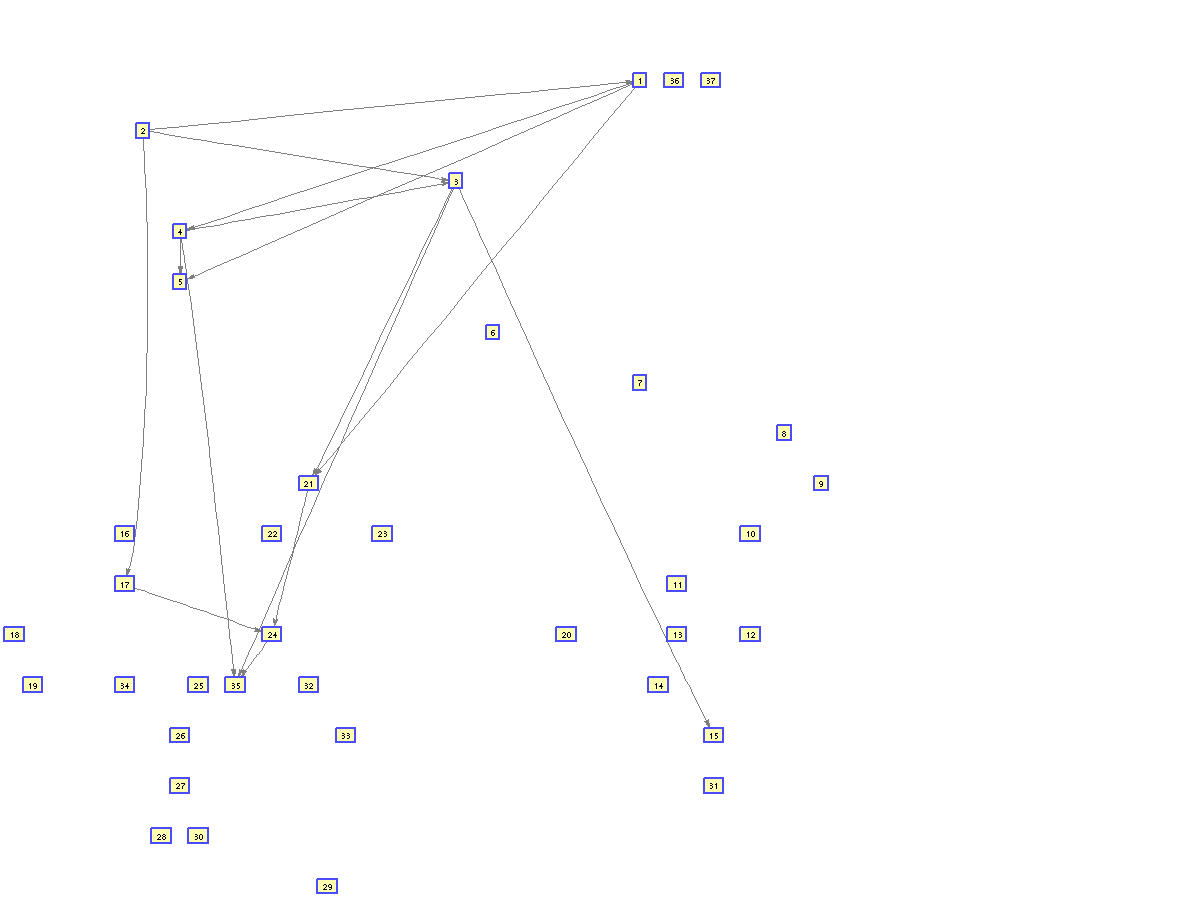

Supplement: Additional file 1 — Mini-website showing all learned network graphs for examples presented. Mini-website showing all learned network graphs at each iteration for the examples presented in the main body of the paper, and a table of the genes involved. [file 1752-0509-3-85-S1.zip › S/net4/grn_fixed_layout_10.png]

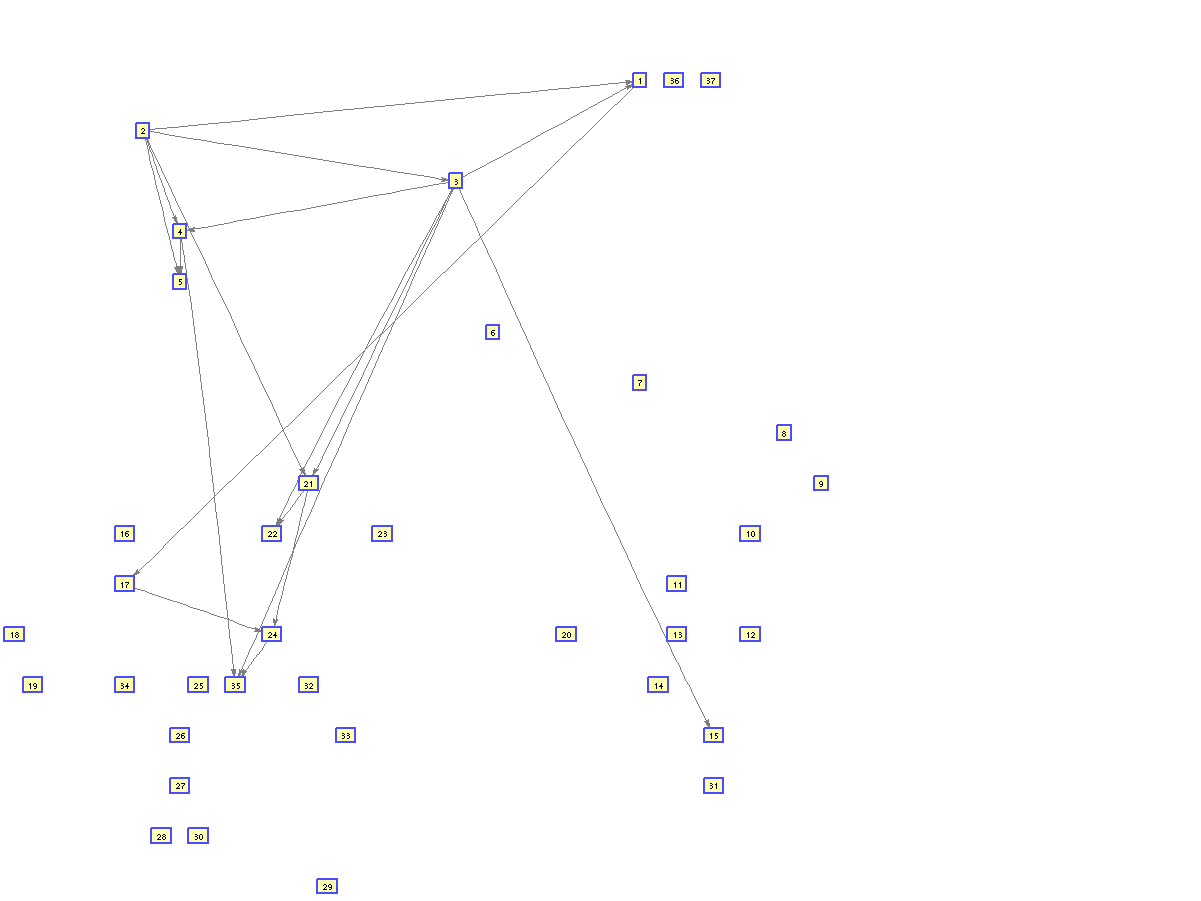

Supplement: Additional file 1 — Mini-website showing all learned network graphs for examples presented. Mini-website showing all learned network graphs at each iteration for the examples presented in the main body of the paper, and a table of the genes involved. [file 1752-0509-3-85-S1.zip › S/net4/grn_fixed_layout_11.png]

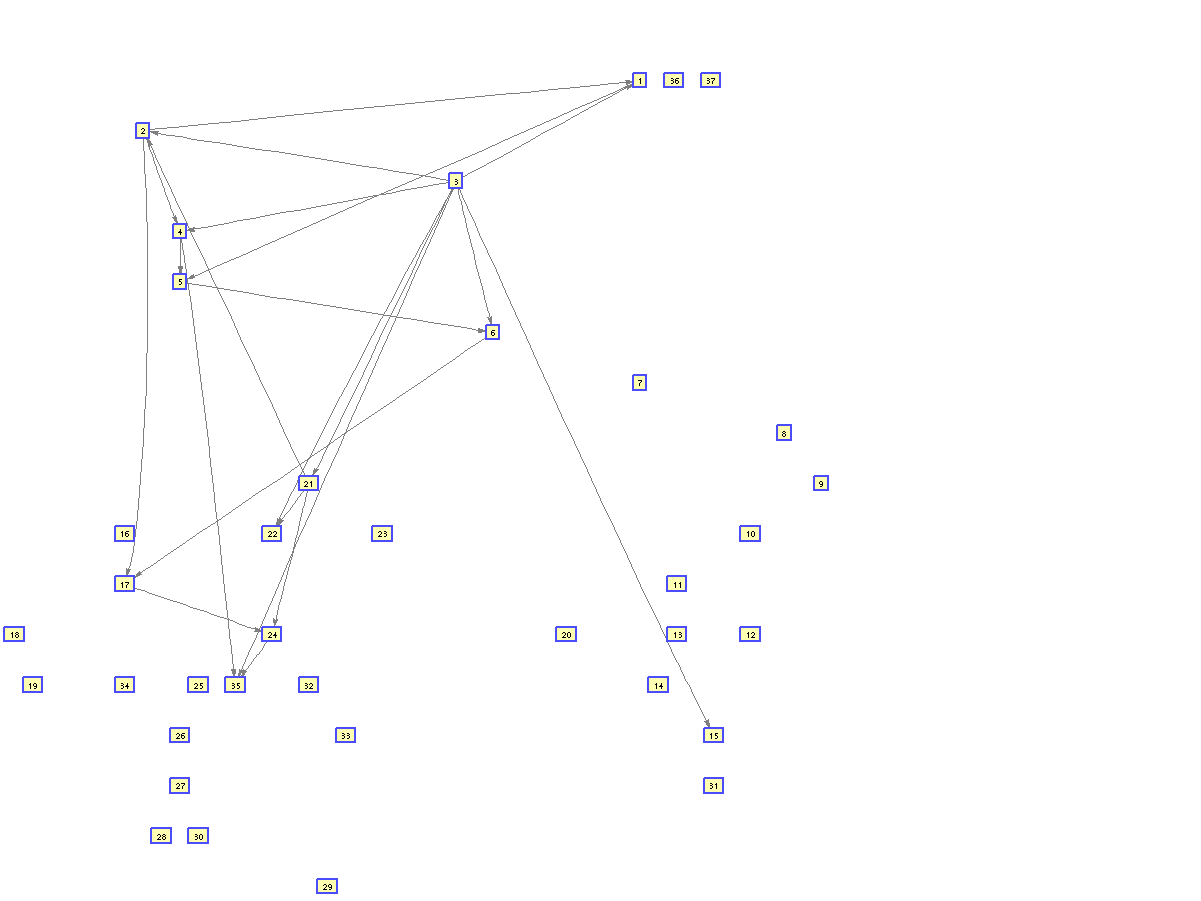

Supplement: Additional file 1 — Mini-website showing all learned network graphs for examples presented. Mini-website showing all learned network graphs at each iteration for the examples presented in the main body of the paper, and a table of the genes involved. [file 1752-0509-3-85-S1.zip › S/net4/grn_fixed_layout_12.png]

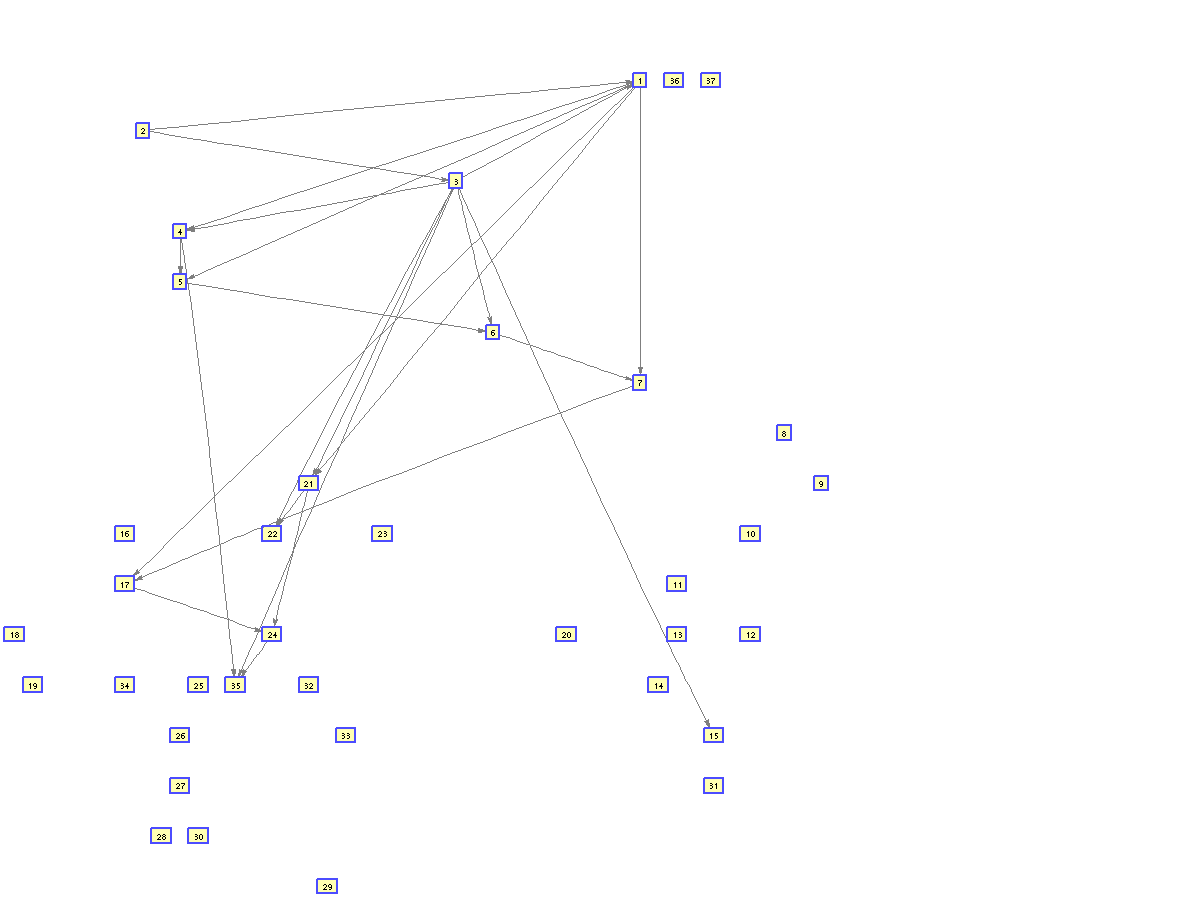

Supplement: Additional file 1 — Mini-website showing all learned network graphs for examples presented. Mini-website showing all learned network graphs at each iteration for the examples presented in the main body of the paper, and a table of the genes involved. [file 1752-0509-3-85-S1.zip › S/net4/grn_fixed_layout_13.png]

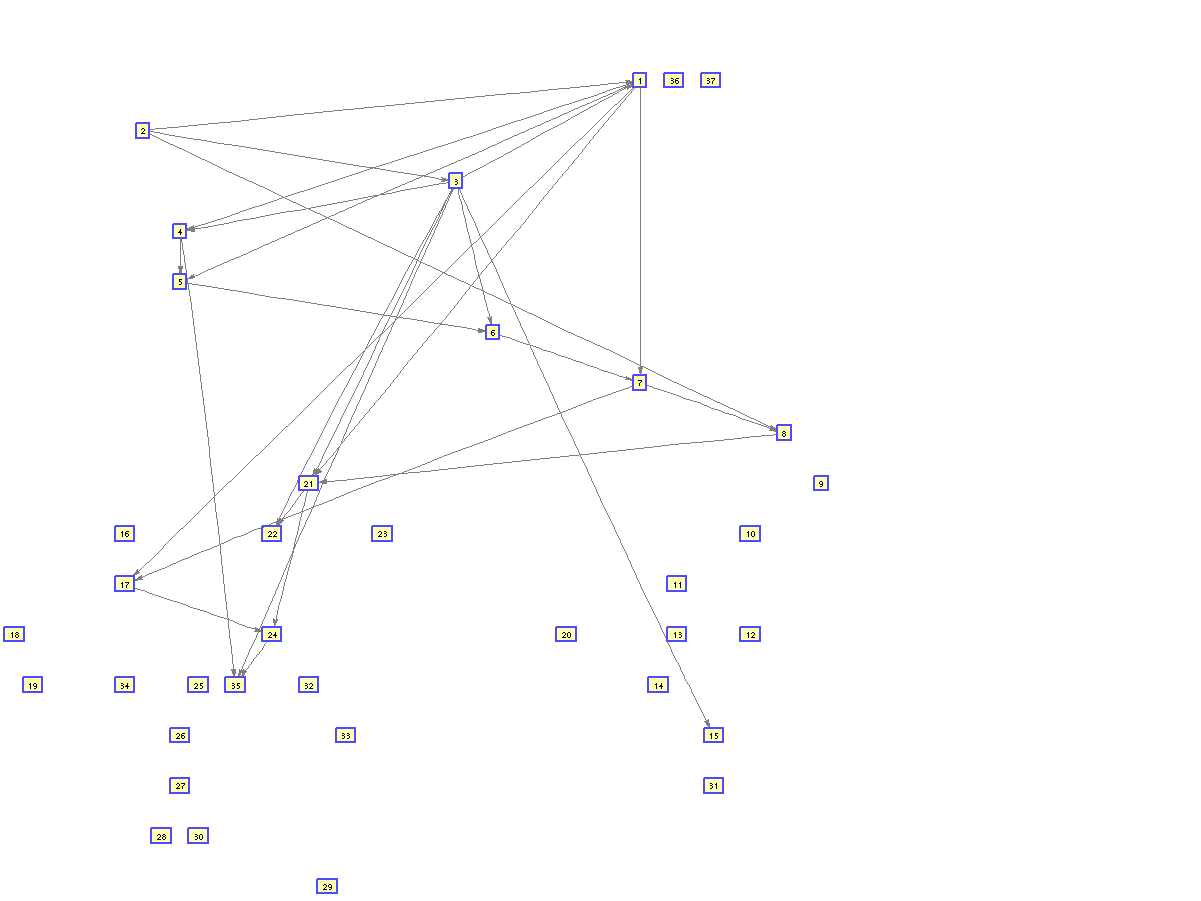

Supplement: Additional file 1 — Mini-website showing all learned network graphs for examples presented. Mini-website showing all learned network graphs at each iteration for the examples presented in the main body of the paper, and a table of the genes involved. [file 1752-0509-3-85-S1.zip › S/net4/grn_fixed_layout_14.png]

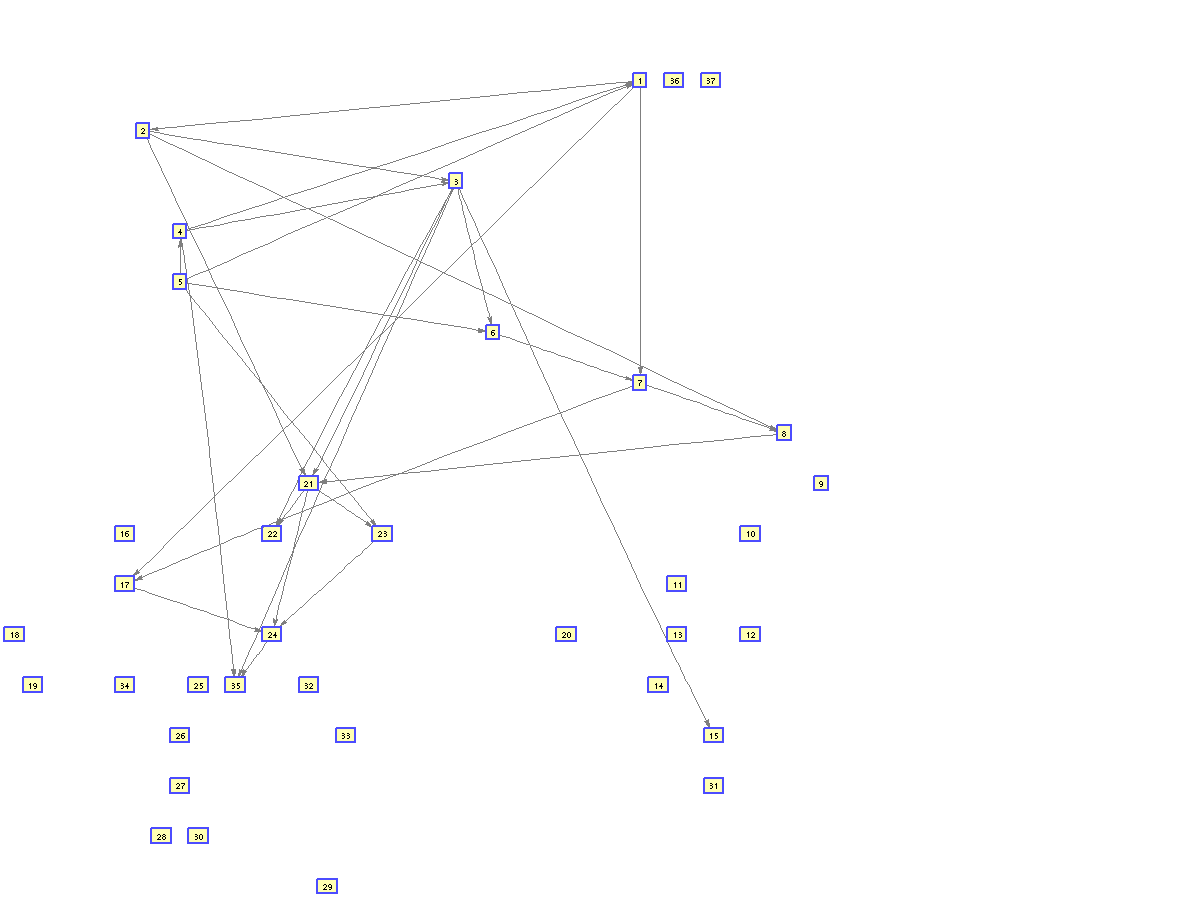

Supplement: Additional file 1 — Mini-website showing all learned network graphs for examples presented. Mini-website showing all learned network graphs at each iteration for the examples presented in the main body of the paper, and a table of the genes involved. [file 1752-0509-3-85-S1.zip › S/net4/grn_fixed_layout_15.png]

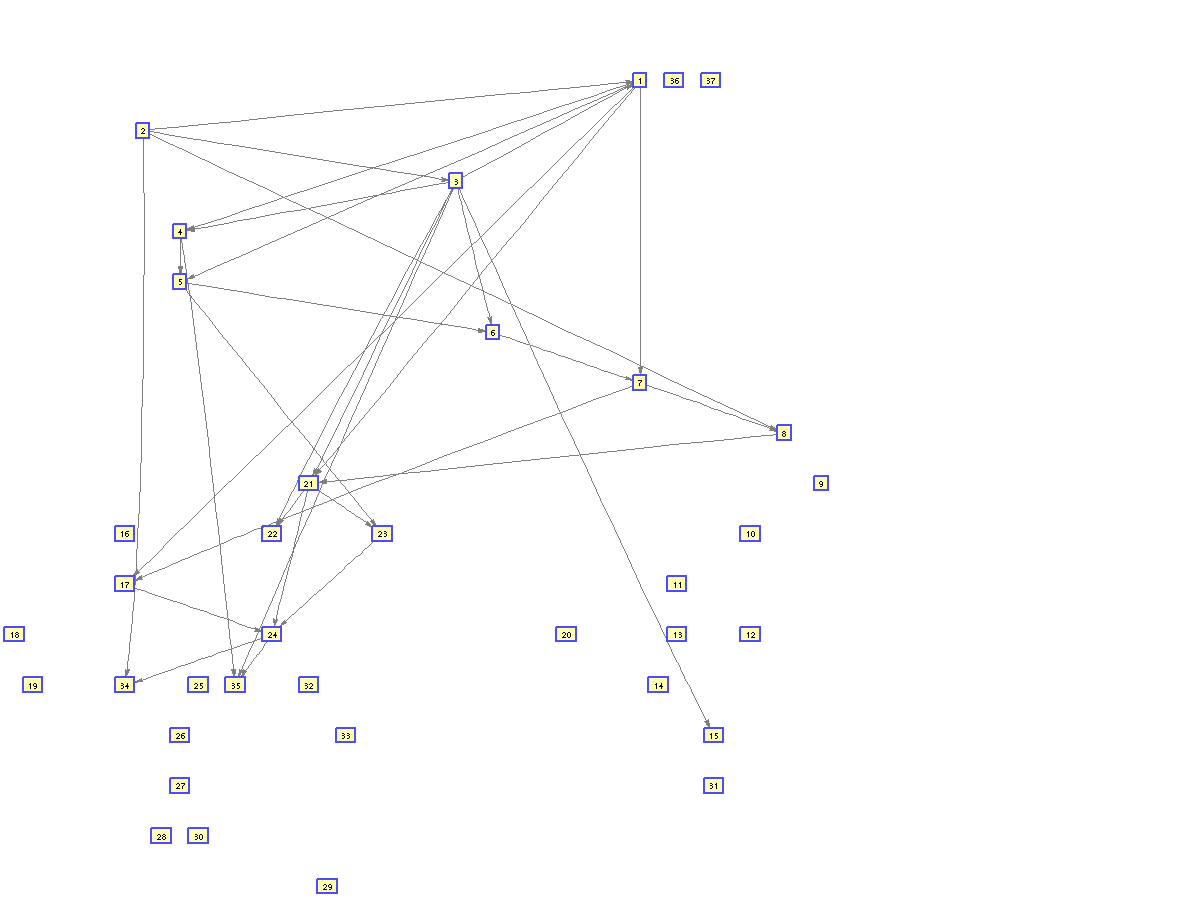

Supplement: Additional file 1 — Mini-website showing all learned network graphs for examples presented. Mini-website showing all learned network graphs at each iteration for the examples presented in the main body of the paper, and a table of the genes involved. [file 1752-0509-3-85-S1.zip › S/net4/grn_fixed_layout_16.png]

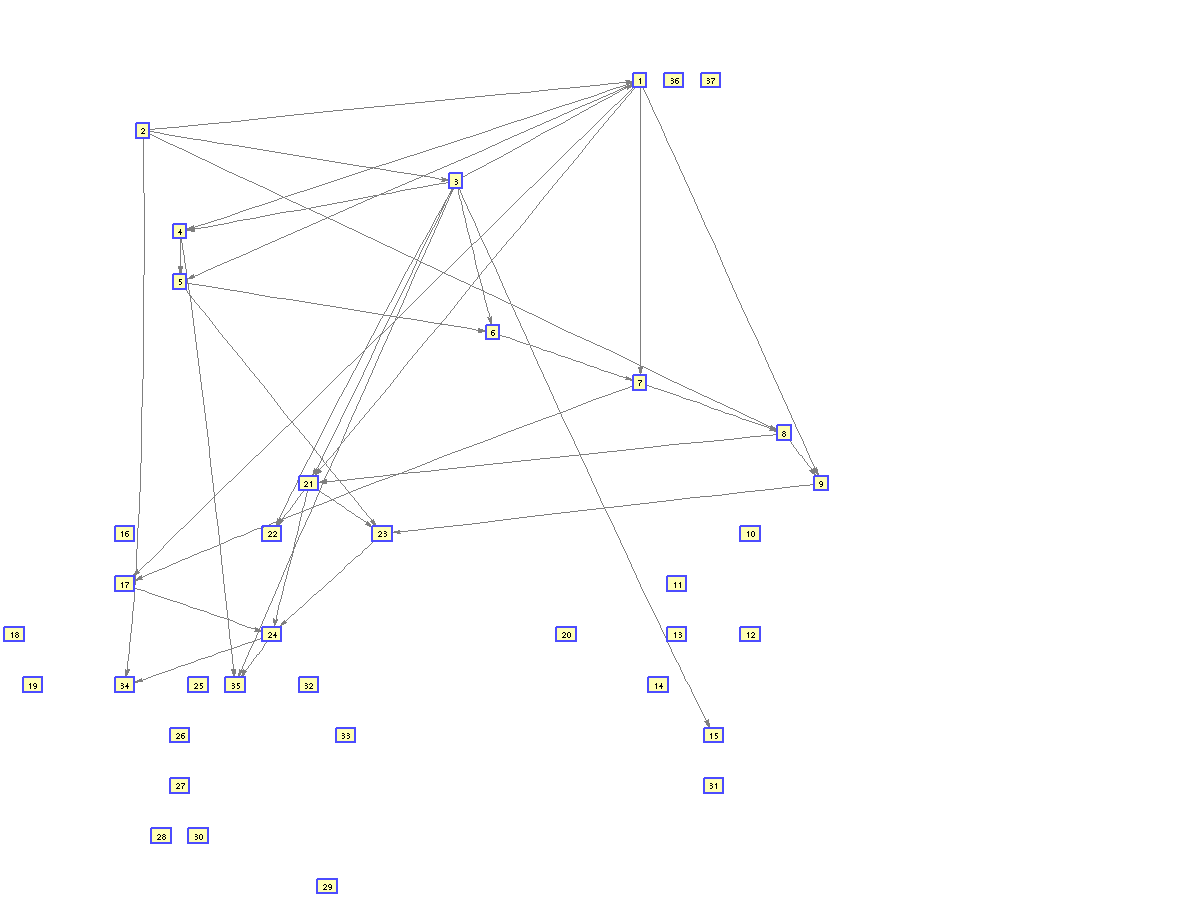

Supplement: Additional file 1 — Mini-website showing all learned network graphs for examples presented. Mini-website showing all learned network graphs at each iteration for the examples presented in the main body of the paper, and a table of the genes involved. [file 1752-0509-3-85-S1.zip › S/net4/grn_fixed_layout_17.png]

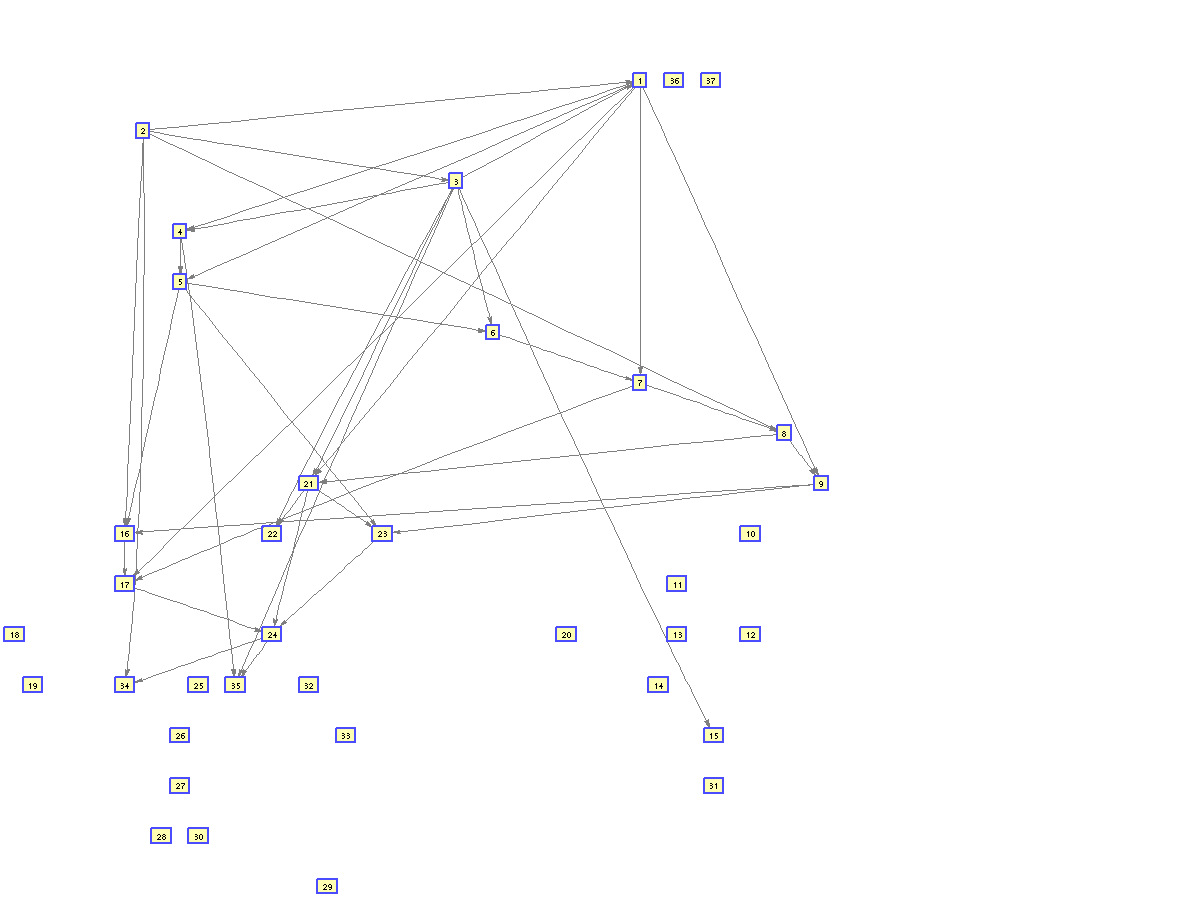

Supplement: Additional file 1 — Mini-website showing all learned network graphs for examples presented. Mini-website showing all learned network graphs at each iteration for the examples presented in the main body of the paper, and a table of the genes involved. [file 1752-0509-3-85-S1.zip › S/net4/grn_fixed_layout_18.png]

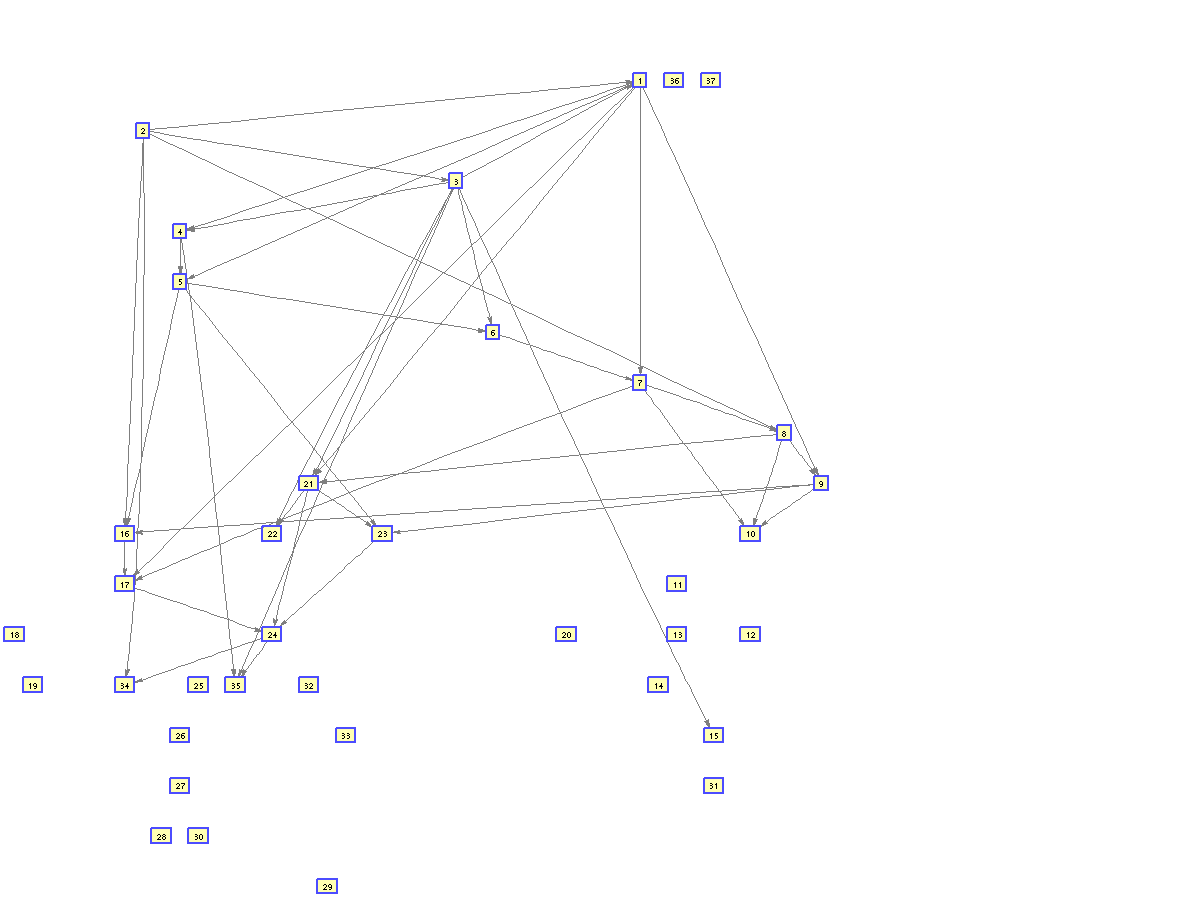

Supplement: Additional file 1 — Mini-website showing all learned network graphs for examples presented. Mini-website showing all learned network graphs at each iteration for the examples presented in the main body of the paper, and a table of the genes involved. [file 1752-0509-3-85-S1.zip › S/net4/grn_fixed_layout_19.png]

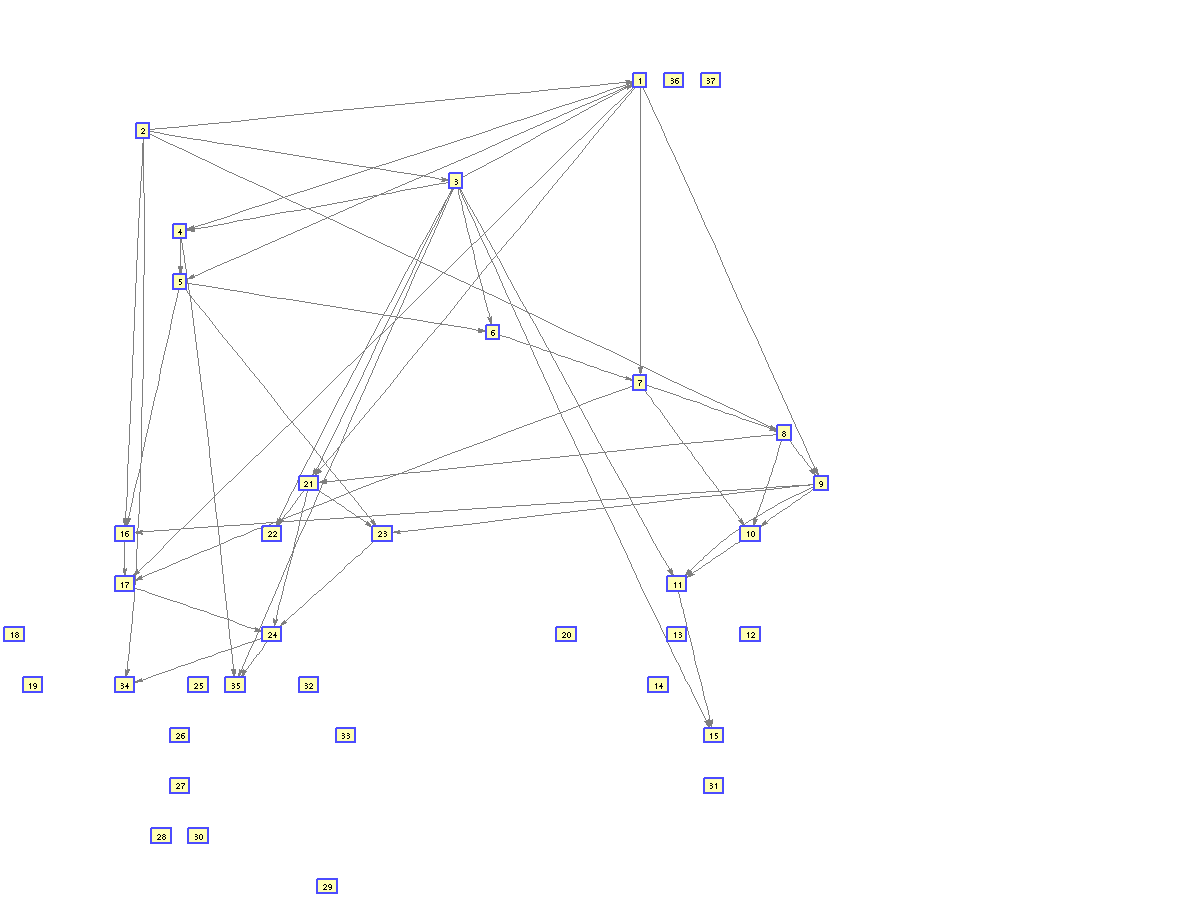

Supplement: Additional file 1 — Mini-website showing all learned network graphs for examples presented. Mini-website showing all learned network graphs at each iteration for the examples presented in the main body of the paper, and a table of the genes involved. [file 1752-0509-3-85-S1.zip › S/net4/grn_fixed_layout_20.png]

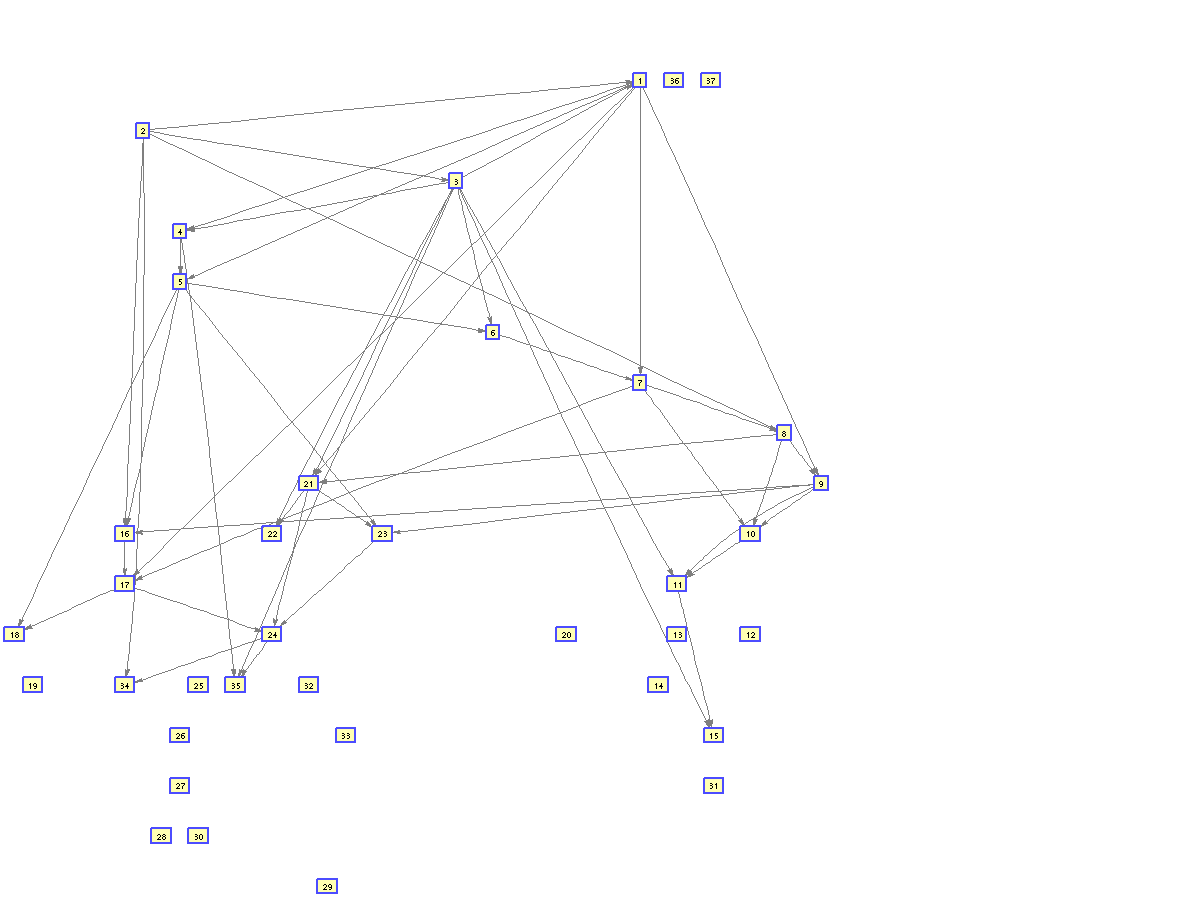

Supplement: Additional file 1 — Mini-website showing all learned network graphs for examples presented. Mini-website showing all learned network graphs at each iteration for the examples presented in the main body of the paper, and a table of the genes involved. [file 1752-0509-3-85-S1.zip › S/net4/grn_fixed_layout_21.png]

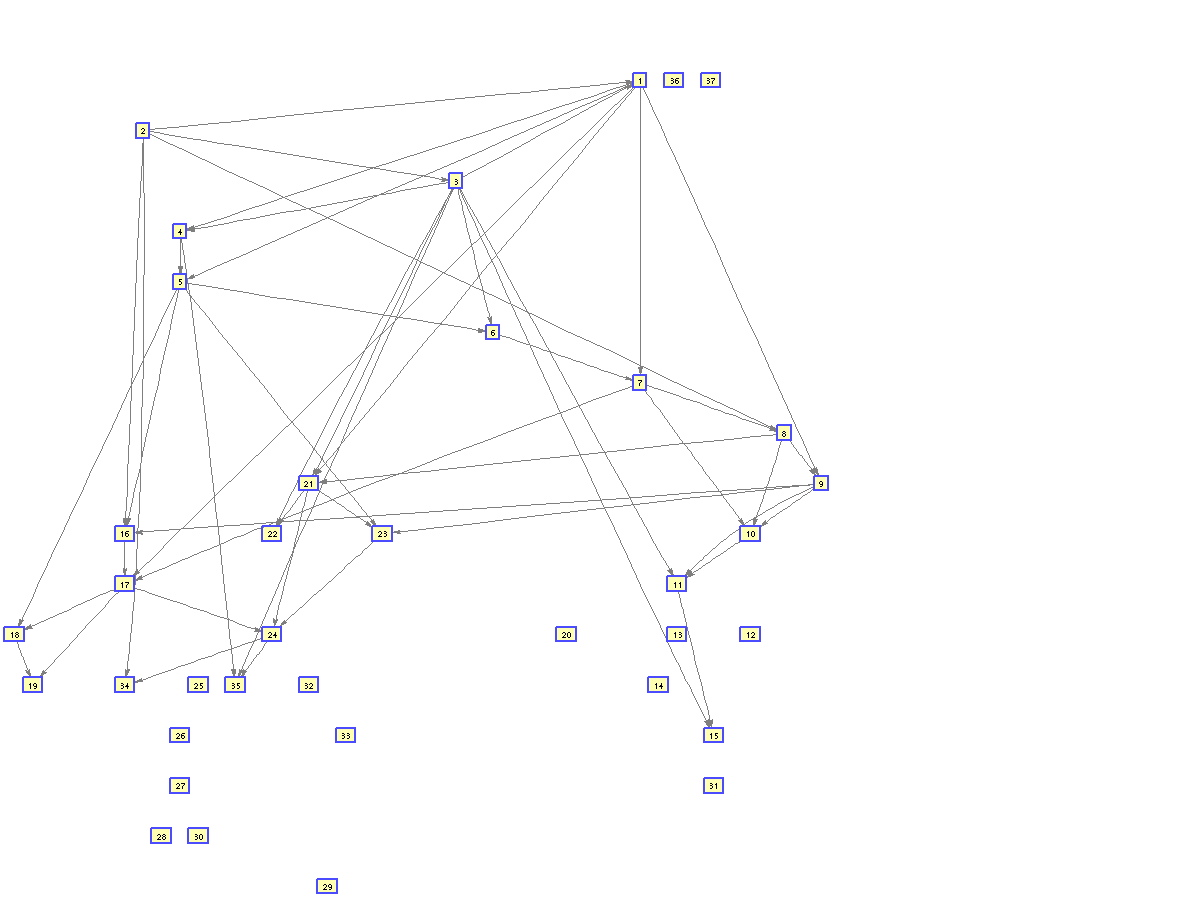

Supplement: Additional file 1 — Mini-website showing all learned network graphs for examples presented. Mini-website showing all learned network graphs at each iteration for the examples presented in the main body of the paper, and a table of the genes involved. [file 1752-0509-3-85-S1.zip › S/net4/grn_fixed_layout_22.png]

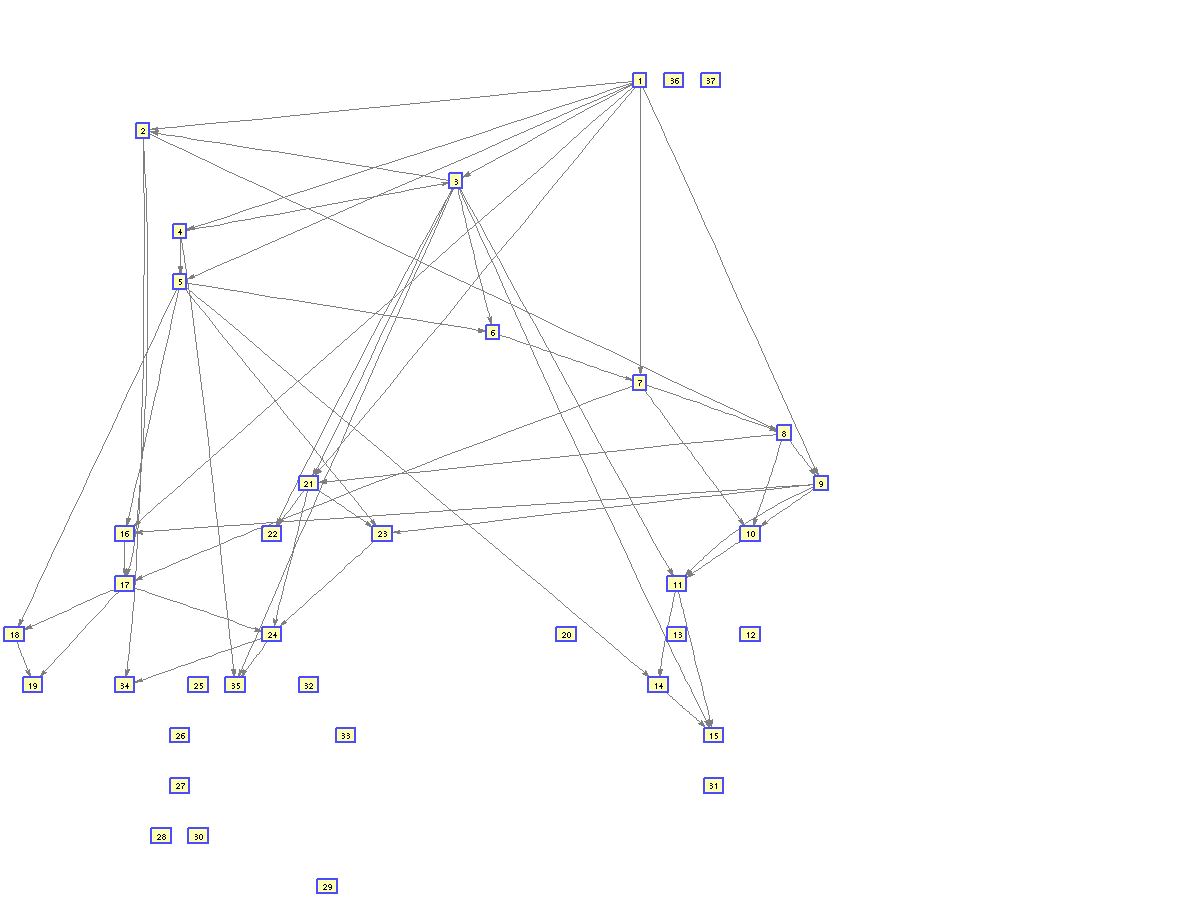

Supplement: Additional file 1 — Mini-website showing all learned network graphs for examples presented. Mini-website showing all learned network graphs at each iteration for the examples presented in the main body of the paper, and a table of the genes involved. [file 1752-0509-3-85-S1.zip › S/net4/grn_fixed_layout_23.png]

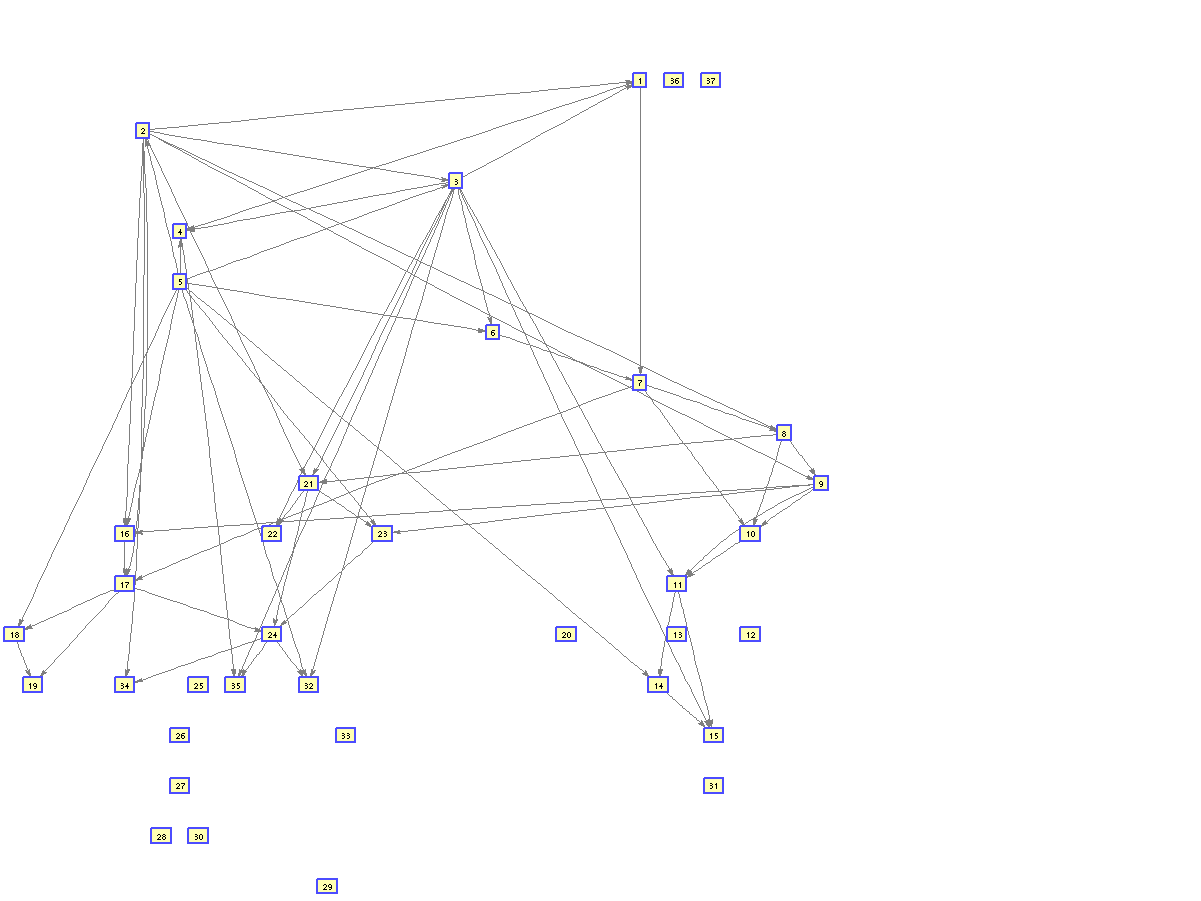

Supplement: Additional file 1 — Mini-website showing all learned network graphs for examples presented. Mini-website showing all learned network graphs at each iteration for the examples presented in the main body of the paper, and a table of the genes involved. [file 1752-0509-3-85-S1.zip › S/net4/grn_fixed_layout_24.png]

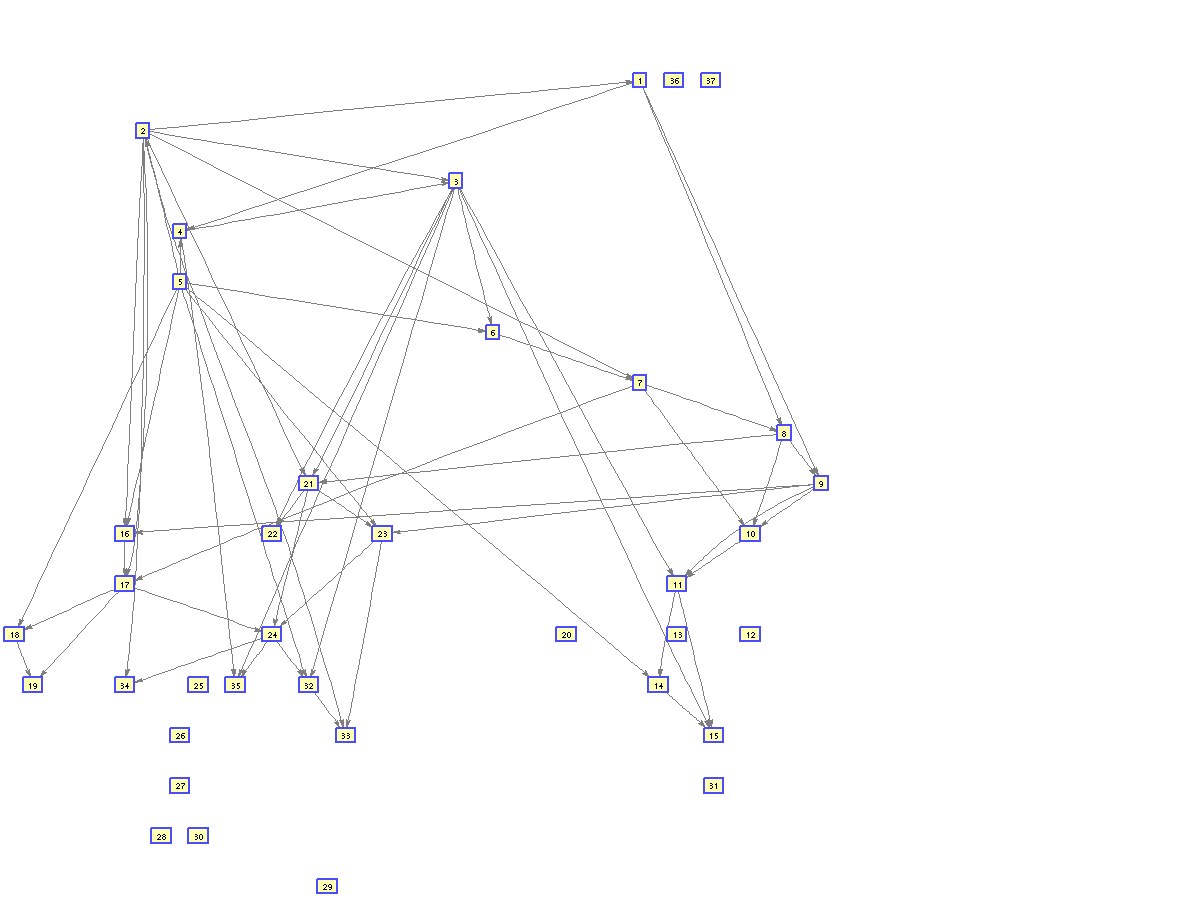

Supplement: Additional file 1 — Mini-website showing all learned network graphs for examples presented. Mini-website showing all learned network graphs at each iteration for the examples presented in the main body of the paper, and a table of the genes involved. [file 1752-0509-3-85-S1.zip › S/net4/grn_fixed_layout_25.png]

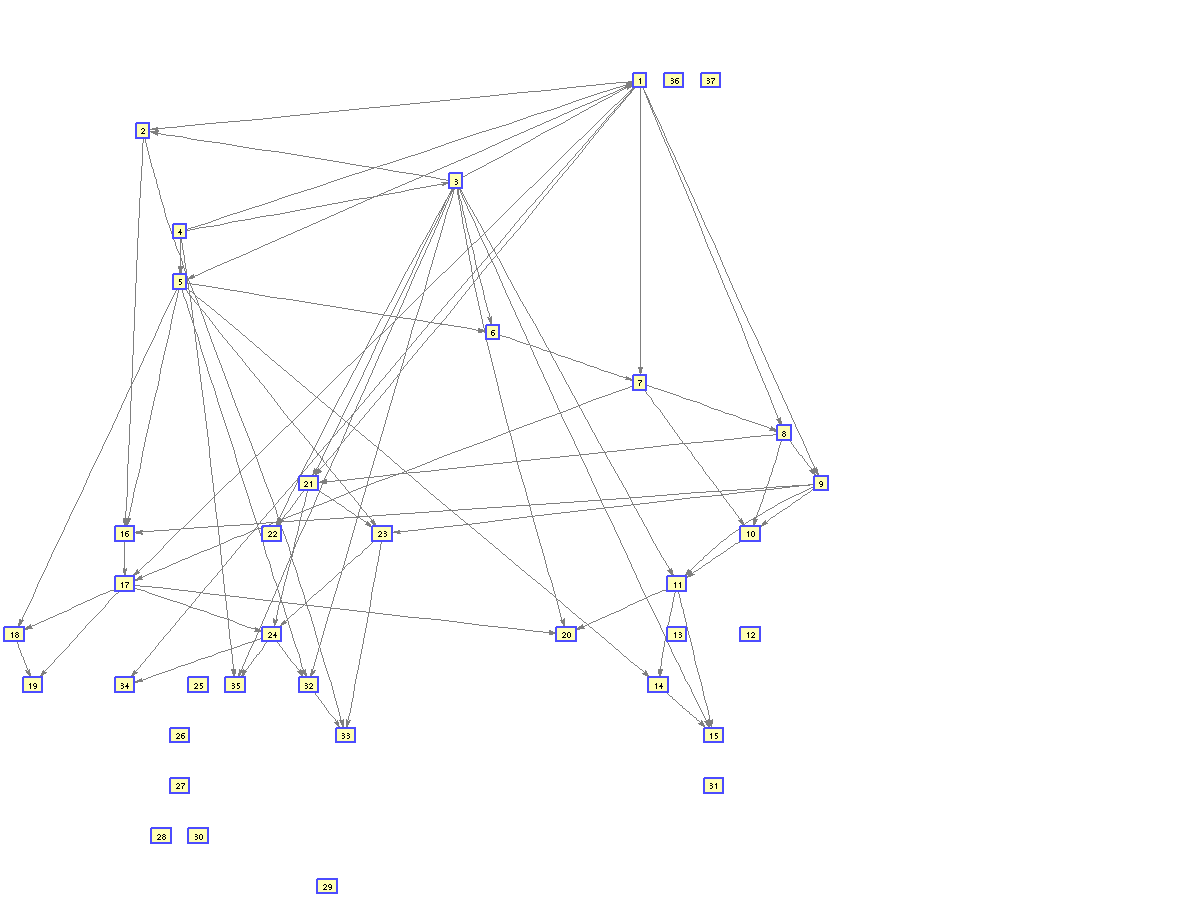

Supplement: Additional file 1 — Mini-website showing all learned network graphs for examples presented. Mini-website showing all learned network graphs at each iteration for the examples presented in the main body of the paper, and a table of the genes involved. [file 1752-0509-3-85-S1.zip › S/net4/grn_fixed_layout_26.png]

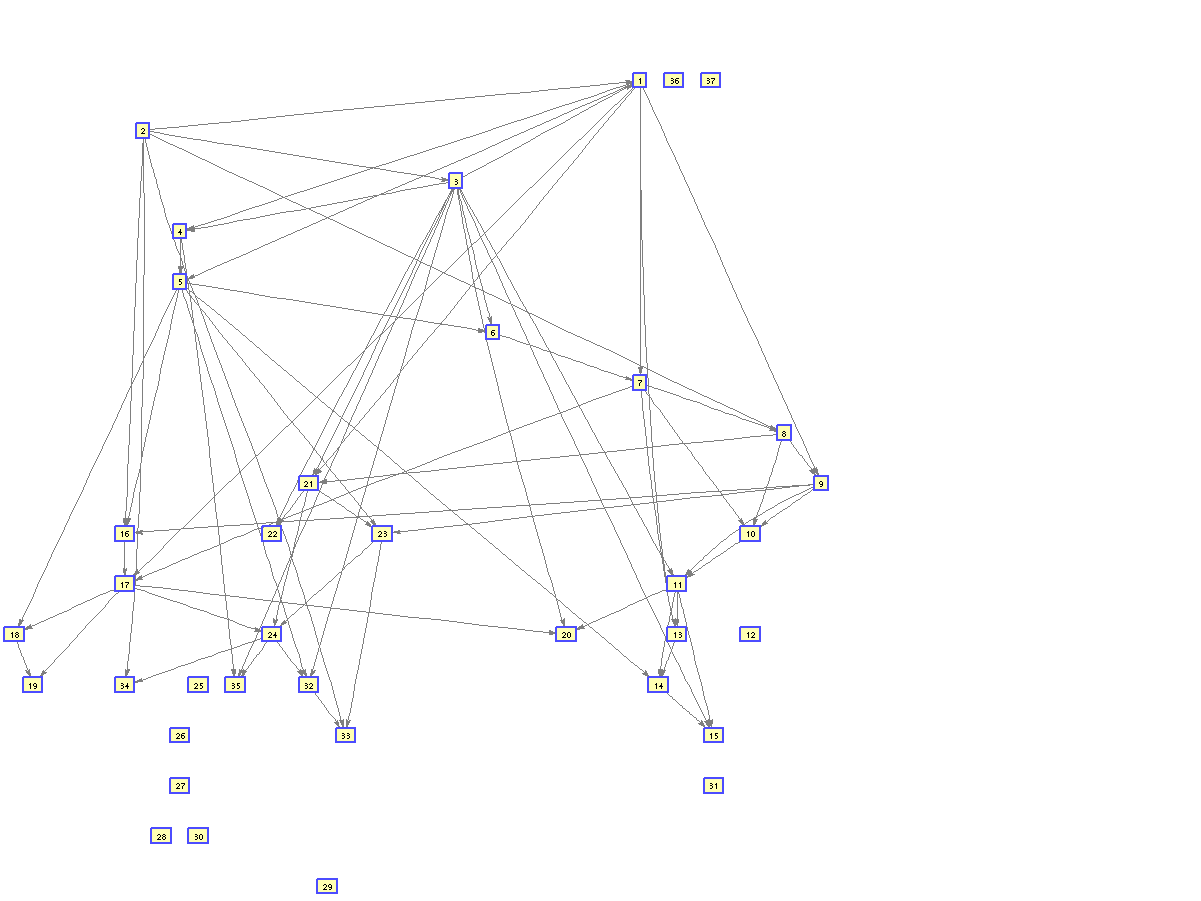

Supplement: Additional file 1 — Mini-website showing all learned network graphs for examples presented. Mini-website showing all learned network graphs at each iteration for the examples presented in the main body of the paper, and a table of the genes involved. [file 1752-0509-3-85-S1.zip › S/net4/grn_fixed_layout_27.png]
